# Discovery and validation of islet regenerative proteins secreted by human multipotent stromal cells

**DOI:** 10.1093/stcltm/szag022

**Published:** 2026-04-29

**Authors:** Xin Y Xie, Nouran N Al-Banaa, Yina Tian, Gillian I Bell, Miljan Kuljanin, Tyler T Cooper, Caleb J Podgers, Ajaya Sharma, Anargyros Xenocostas, Gilles A Lajoie, David A Hess

**Affiliations:** Department of Physiology and Pharmacology, Schulich School of Medicine and Dentistry, Western University, London, ON N6A 5C1, Canada; Molecular Medicine Research Laboratories, Robarts Research Institute, London, ON N6A 5B7, Canada; Department of Physiology and Pharmacology, Schulich School of Medicine and Dentistry, Western University, London, ON N6A 5C1, Canada; Molecular Medicine Research Laboratories, Robarts Research Institute, London, ON N6A 5B7, Canada; Department of Physiology and Pharmacology, Schulich School of Medicine and Dentistry, Western University, London, ON N6A 5C1, Canada; Molecular Medicine Research Laboratories, Robarts Research Institute, London, ON N6A 5B7, Canada; Department of Physiology and Pharmacology, Schulich School of Medicine and Dentistry, Western University, London, ON N6A 5C1, Canada; Molecular Medicine Research Laboratories, Robarts Research Institute, London, ON N6A 5B7, Canada; Department of Biochemistry, Western University, London, ON N6A 5C1, Canada; Department of Biochemistry, Western University, London, ON N6A 5C1, Canada; Molecular Medicine Research Laboratories, Robarts Research Institute, London, ON N6A 5B7, Canada; Department of Physiology and Pharmacology, Schulich School of Medicine and Dentistry, Western University, London, ON N6A 5C1, Canada; Molecular Medicine Research Laboratories, Robarts Research Institute, London, ON N6A 5B7, Canada; Department of Haematology, London Health Sciences Centre, London, ON N6A 5W9, Canada; Department of Biochemistry, Western University, London, ON N6A 5C1, Canada; Department of Physiology and Pharmacology, Schulich School of Medicine and Dentistry, Western University, London, ON N6A 5C1, Canada; Molecular Medicine Research Laboratories, Robarts Research Institute, London, ON N6A 5B7, Canada

**Keywords:** diabetes mellitus, multipotent stromal cells, stem cells, islet regeneration, proteomics

## Abstract

**Introduction:**

Diabetes affects >500 million people worldwide. Despite insulin therapy, most patients develop devastating complications, emphasizing the need for curative strategies. Human multipotent stromal/stem cells (MSC) secrete factors that promote islet regeneration. We previously showed that intrapancreatic (iPan) delivery of Wnt-activated MSC-conditioned media (Wnt+ CM) stimulates islet regeneration without cell transfer. This study aimed to identify and functionally validate specific MSC-secreted proteins with islet regenerative potential.

**Methods:**

Comprehensive mass spectrometry-based, quantitative proteomic analyses comparing Wnt-pathway activated versus untreated MSC secretomes were cross-referenced with a prior dataset distinguishing regenerative from nonregenerative MSC CM. Proteins enriched in both conditions were iPan-injected individually or in combination into streptozotocin-treated NOD/SCID mice. Nonfasting glucose, glucose tolerance, beta cell mass, islet morphology, and islet cell proliferation were assessed at 4- and 32-days post-treatment.

**Results:**

Cross-referenced secretome analyses identified eight proteins implicated in islet regeneration: CALU, CTSB, FAM3C, GAL1, PPIA, PSAP, SOD1, and TGM2. A single iPan-injection of the 8-protein combination significantly lowered hyperglycemia, improved glucose tolerance, and increased beta cell mass, comparable to Wnt+ CM. Regenerative effects such as increased beta cell proliferation appeared as early as day 4. Single-protein testing identified CALU and SOD1 as leading candidates, improving glucose tolerance and reducing nonfasting glucose.

**Conclusion:**

This study defines a set of MSC-secreted proteins that promote islet regeneration *in vivo*, supporting the development of protein-based biologics to preserve or restore beta cell function during diabetes, with potential applications alongside islet replacement therapies to enhance graft survival and function.

## Graphical abstract

**Graphical Abstract:**
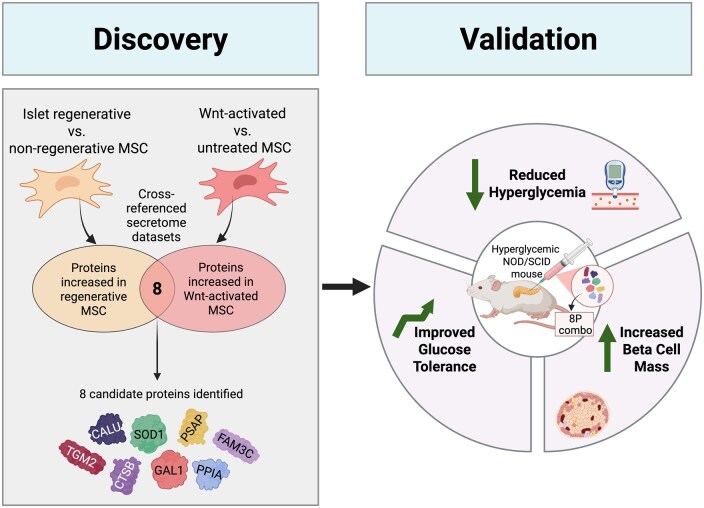

## Introduction

According to the International Diabetes Federation, >500 million adults are currently living with diabetes, and this number is expected to surpass one billion by 2050, making diabetes a global health care crisis.^1^ While life-saving insulin therapy remains the cornerstone of glycemic management, it is not curative, and lifelong dependence on exogenous insulin places a substantial emotional and clinical burden on individuals with type 1 diabetes (T1D). Moreover, life expectancy in people with diabetes is reduced by 10-15 years, largely due to premature cardiovascular and renal complications.^2^ Therefore, strategies aimed at restoring endogenous beta cell function, whether through islet regeneration or replacement, represent curative approaches to improve long-term diabetes outcomes.^3^

The Edmonton protocol provided proof-of-concept that islet transplantation^4^^,^^5^ can restore insulin production in recipients with T1D, with high rates of insulin independence achieved at 1 year.^6^ However, long-term efficacy remains limited, as only ∼10% of recipients maintain insulin independence at 5 years, largely due to progressive graft loss despite immunosuppressive therapy.^7^ Furthermore, the severe shortage of deceased donor islets restricts this therapy to a small subset of individuals with hypoglycemia unawareness.^8^ To address these limitations, efforts have focused on developing renewable sources of islets. Directed differentiation of human pluripotent stem cells (PSC) potentially enables the generation of an unlimited supply of ‘islet-like’ cells for transplantation.^9^ In the ongoing VX-880 FORWARD trial using PSC-derived islets,^10^ 12 of 14 participants with T1D showed engrafted islet function evidenced by C-peptide detection, and 10 of these achieved insulin independence at 1 year. However, challenges remain in the path towards widespread application of this approach. One participant died from cryptococcal meningitis exacerbated by immunosuppression, and PSC-derived islets are anticipated to undergo deletion within 3-5 years, similar to the Edmonton protocol, due to ongoing autoimmunity.^10^ Thus, alternate strategies to extend graft survival while minimizing life-long immunosuppression are warranted.

In recent years, the concept of regenerating functional beta cell mass *in situ*, with or without co-administration of replacement islets, has emerged as a compelling therapeutic approach for T1D. Regenerative medicine alternatives seeking to stimulate the innate capacity for islet renewal through the induction of beta cell proliferation,^11^ differentiation from islet cell types such as alpha cells,^12^ or new islet formation from pancreas-resident endocrine progenitors^13^ have been reported. However, identification of the complex array of molecular effectors that modulate these multicellular islet regenerative pathways remains a critical challenge.

Human bone marrow is an accessible source of multipotent stromal cells also known as mesenchymal stem cells (MSC), which hold considerable promise for diabetes therapy. Owing to their ease of isolation, robust proliferative capacity in culture, low immunogenicity, and established safety profile,^14–17^ MSCs are widely used in clinical trials.^18^ Distributed with blood vessels during development,^19^ MSC release regenerative, pro-angiogenic, and immunosuppressive factors through extracellular vesicles,^20^^,^^21^ earning them the description ‘sentinels of tissue injury’.^22^^,^^23^ Human MSC transplantation into Streptozotocin (STZ)-treated immunodeficient mice^24^ has shown donor-dependent heterogeneity in the ability to induce islet regeneration via paracrine mechanisms.^25^ Using mass-spectrometry-based proteomic discovery, we have shown that Wnt-signalling was consistently activated in islet regenerative MSC via autocrine production of Wnt5A, whereas nonregenerative MSC repressed Wnt-signals via Dickkopf-related protein 1 and 3 inhibition.^24^^,^^25^ Remarkably, activating Wnt-signaling during MSC expansion through GSK3-inhibition produced Wnt-activated conditioned media (Wnt+ CM), which enhanced beta cell survival and proliferation when co-cultured with human islets.^25^ Furthermore, intrapancreatic (iPan)-injection^26^ of Wnt+ CM into hyperglycemic NOD/SCID mice reduced hyperglycemia, recovered glucose tolerance, and increased beta cell mass without the need for MSC transfer.^27^ However, specific proteins responsible for islet regeneration within the CM remain undefined. Therefore, identification of islet regenerative proteins is required to move towards targeted intervention. Unlike live cell therapy, protein-based therapeutics offer distinct advantages in scalability, manufacturing consistency, and safety.^28^ Thus, discovery and validation of islet regenerative effectors secreted by MSC may lay the groundwork for the development of novel biologic agents to target islet regeneration *in situ* or improve beta cell survival and function in replacement trials.

Herein, we compared the secretome of Wnt+ MSC CM to that of Untreated MSC CM and cross-referenced the novel dataset with a previously published screen comparing the secretome of islet regenerative versus nonregenerative MSC.^25^ We discovered eight MSC-secreted protein candidates selectively enriched in both screens and functionally validated the islet regenerative potential of these proteins *in vivo*, both individually and in combination.

## Methods

### Generation of MSC CM

Human bone marrow (BM) aspirates were obtained from healthy donors with informed consent at the London Health Science Center. This protocol was approved by the Human Research Ethics Board at Western University (REB#12934). Bone marrow samples from a total of six donors (two females age 41 and 49, and four males aged 53, 55, 59, and 61) were used to establish MSC preparations by adherence to plastic in AmnioMax C-100 Complete Media (Thermo Fisher Scientific). MSC at passage 4 were exposed to 10 mM CHIR99021 (AbMole) or DMSO vehicle to generate Wnt+ CM and Untreated CM as previously described.^27^ After 24 h of treatment, CM was collected and concentrated in 3 kDa centrifugal filter units (Millipore) at 2800 × *g* for 75 min. Concentrated CM contained MSC-secreted proteins larger than 3 kDa, including both soluble proteins and extracellular vesicles.^20^^,^^21^^,^^29^ Total protein concentration was quantified using the Pierce 660 nm Assay (Thermo Fisher Scientific). Flow cytometry (BD LSR II) on co-harvested MSC was used to confirm the elevation of intracellular beta-catenin in Wnt+ CM compared to Untreated CM, as previously shown.^13^ See Supplementary Methods for further details.

### Proteomic workflow

Label-free, mass-spectrometry-based total secretome analyses were performed to compare Wnt+ CM with Untreated CM (*N* = 3). CM samples were precipitated and digested with LysC and trypsin before acidification. Peptide fragments (1 µg) were injected into a nanoAcquity HPLC (Waters Corporation) coupled to a Q Exactive Plus Orbitrap mass spectrometer (Thermo Fisher Scientific). Data was processed using MaxQuant v1.5.8.30 software (Max Planck Institute) and the Human Uniprot database (20,264 entries). Bioinformatics was performed using Perseus (v1.5.8.5), filtering for unique peptides in at least 2 of 3 replicates.^30^ See Supplementary Methods for further details.

### Cross-referenced secretome datasets

Proteins upregulated in Wnt+ CM relative to Untreated CM were cross-referenced with a previously published screen comparing the secretomes of regenerative versus nonregenerative MSC.^25^ By combining both datasets, we identified eight shared proteins uniquely upregulated in both Wnt+ and regenerative CM. Full-length, recombinant human versions of the identified proteins were acquired from commercially available sources as detailed in Table S1.

### Intrapancreatic injection of regenerative proteins and glucose monitoring *in vivo*

All experimental procedures were approved by the Animal Care Committee at Western University (AUP#2023-014). Beta cell ablation was induced in adult male and female NOD/SCID mice (Jackson Laboratory) at 8-10 weeks of age via intraperitoneal injection of STZ (Sigma-Aldrich) at 35 mg/kg/day for five consecutive days (day 0-4) as previously described.^31–33^ Approximately equal numbers of males and females were randomized into each treatment group. Mice with nonfasting plasma glucose (NFPG) levels of 13-25 mmol/L on day 10 were included in the experiment. Animals with NFPG outside the range were excluded due to incomplete beta cell ablation or irreversible hyperglycemia.^26^^,^^34^ On day 10, mice were anesthetized and iPan-injected (20 μL) with either unconditioned basal culture media (vehicle control), Wnt+ CM (positive control, 4-8 µg total protein), a combination of the eight identified MSC-secreted proteins (100 ng each), or individual proteins (100 ng). Multiple independent BM-MSC donor samples were used to generate the Wnt+ CM in our study, as indicated by the *N* values. Each recombinant protein was administered at 100 ng per mouse, a dose selected from pilot titrations and informed by published *in vivo* precedents showing bioactivity of recombinant proteins at nanogram-microgram levels in mice.^35^^,^^36^ The NFPG levels were monitored weekly via tail vein puncture using a FreeStyle Lite glucometer (Abbott Diabetes Care), as previously described.^27^ Experiment duration was divided into short-term (day 14) and long-term (day 42) euthanasia timepoints. All mice received an intraperitoneal injection of 100 μL (2.5 mg/mL) EdU 24 h before sacrifice. For glucose tolerance tests in the long-term groups, mice were fasted for 4 h before intraperitoneal injection of sterile glucose solution (2.0 g/kg). Plasma glucose was measured via tail vein puncture immediately before glucose injection (0 min), and at 5-, 10-, 15-, 30-, 45-, 60-, 90-, and 120-min post-injection. A group of healthy control mice was included, which received CAB vehicle instead of STZ from day 0 to 4 and did not undergo iPan-injection on day 10. These mice were not subdivided into short- or long-term timepoints, as no differences were expected in these variables for adult NOD/SCID mice. Body weight of all mice was monitored throughout the experiment, and % weight change was calculated as an indicator of overall health status.

### Immunohistochemistry and immunofluorescent analyses

The pancreas of each mouse was retrieved and cryo-sectioned (10 μm) such that each slide contained three sections >200μm (>2 islet diameters) apart.^27^ Product information and antibody concentrations used for staining are detailed in Table S1. Pancreas sections were stained for insulin using a DAB detection system (Vector Laboratories) to quantify beta cell mass, islet size, and islet number using 20X Aperio AT2 Digital Slide Scanner and Aperio ImageScope (v12.4.6, Leica Biosystems), counting all islets within three sections per mouse, >200μm apart. Beta cell mass was calculated by beta cell area ÷ total area × pancreas weight. Pancreas sections were also stained with immunofluorescent antibodies to detect insulin and glucagon, with cell nuclei visualized using DAPI. Photomicrographs were captured using the Axio Imager Z2 fluorescent microscope and ZEN Blue software (Carl Zeiss), and images were analyzed using ImageJ (US National Institutes of Health). Pancreas sections were also co-stained for EdU-incorporation using the Click-It system (Thermo Fisher Scientific) to assess cell proliferation. For each islet, both the total islet area and the insulin+ region were manually delineated, and the number of EdU+ nuclei within these areas was quantified. See Supplementary Methods for further details.

### Statistical analyses

Statistical analyses were performed using GraphPad Prism v10.5.0 (San Diego). To ensure methodological consistency and minimize bias, all samples were processed and analyzed using standardized protocols, and all analyses were performed in a blinded manner. All data were presented as mean ± SEM unless otherwise specified. Proteomic data were compared using paired Student’s *t*-tests. Glucose data were analyzed using two-way ANOVA with Dunnett’s multiple comparisons test, while all other endpoints were analyzed using one-way ANOVA with Dunnett’s test.

## Results

### Cross-referenced secretome analyses revealed eight MSC-secreted proteins implicated in islet regeneration

Human MSC transplanted into STZ-treated mice demonstrate donor-dependent variability to reduce hyperglycemia.^24^^,^^31^^,^^32^ Wnt-signalling was consistently activated in MSC with regenerative capacity alongside increased secretion of proteins associated with matrix remodeling, immunosuppressive, and proangiogenic properties.^25^ In contrast, nonregenerative MSC uniquely secreted proteins known to promote inflammation and negatively regulate Wnt-signalling. After stimulating the Wnt-pathway using GSK3-inhibition with CHIR99021, the conditioned media generated (Wnt+ CM) augmented beta cell survival and proliferation when exposed to human islets *in vitro.*^25^ Therefore, we performed label-free, mass-spectrometry-based secretome analyses to identify putative islet regenerative proteins contained in the Wnt+ MSC CM.

**Figure 1:**
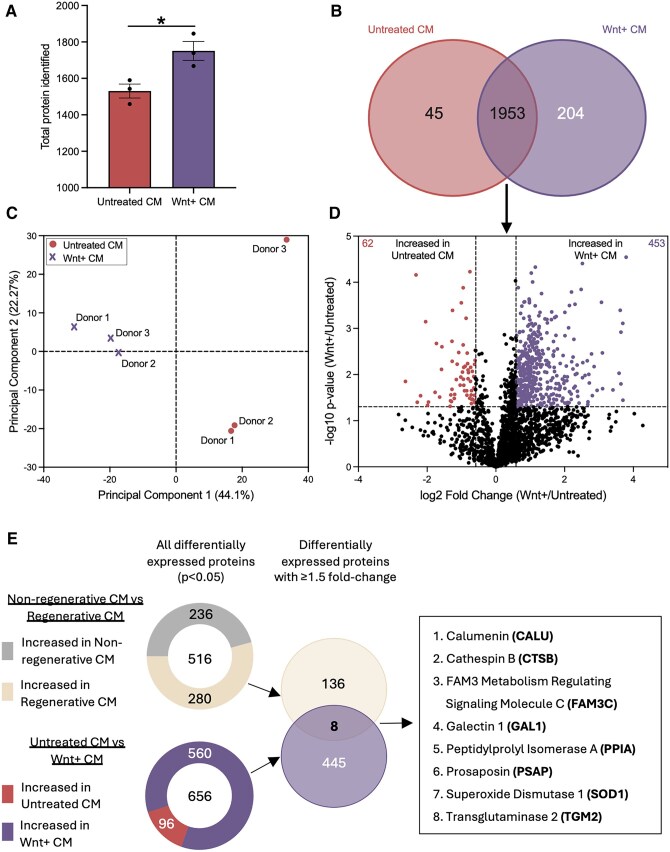
Cross-referenced secretome analyses revealed eight proteins upregulated in Wnt+ MSC CM and in regenerative MSC CM. (A) Total number of unique proteins identified in CM from Untreated MSC versus Wnt+ MSC (*N* = 3). Wnt+ CM showed a greater number of secreted proteins. (B) Venn diagram showing the number of exclusive and overlapping proteins identified in Untreated CM and Wnt+ CM. (C) Principal component analysis showed reduced heterogeneity in proteins secreted from Wnt+ MSC compared to Untreated MSC in three matched donors. (D) Representative volcano plot of differentially expressed secreted proteins. Proteins significantly increased in Wnt+ CM (purple) and Untreated CM (red) are highlighted [cutoff: ≥1.5-fold change (Log2FC ≥ 0.6 or ≤-0.6) and *P < .05]. (E) Mass spectrometry-based total secretome content were cross-referenced between regenerative versus nonregenerative MSC CM and Wnt+ versus Untreated CM. Overlap between the two screens revealed eight proteins with increased expression in both regenerative MSC CM and Wnt+ CM. Data was compared using Paired Student’s *t*-test, *P < .05).

Treatment of MSC with CHIR99021 (*N* = 7) consistently increased intracellular beta-catenin levels by >50%, confirming the activation of canonical Wnt-signalling (Figure S1A-C).^13^ Next, we compared total protein content in donor-matched Untreated CM (*N* = 3) versus Wnt+ CM (*N* = 3) as previously described.^24^^,^^25^ The mean number of proteins identified in Wnt+ CM (1751 ± 52) was increased compared to Untreated CM (1530 ± 38), suggesting Wnt-pathway activation augmented protein secretion (Figure 1A). Among the 1953 proteins commonly detected in at least 2 of 3 replicates (*N* = 3) from both Wnt+ CM and Untreated CM (Figure 1B), principal component analysis revealed greater donor heterogeneity in Untreated CM, which was reduced following Wnt-activation with CHIR99021 (Figure 1C). Using differential expression analyses (*P < .05, ≥1.5 fold-change), we identified 453 proteins with increased expression in Wnt+ CM (Figure 1D and Table S2). In contrast, only 62 proteins were increased in Untreated CM (Figure 1D and Table S3). Notably, Untreated CM included increased expression of Wnt-pathway inhibitors such as DKK1/3.

To narrow the search for islet regenerative effectors, the 453 proteins upregulated in Wnt+ CM were cross-referenced with 144 proteins with increased expression in regenerative versus nonregenerative MSC CM previously reported in Kuljanin et al.^25^ Cross-referencing revealed only eight proteins with increased expression in both screens (Figure 1E). The known biological functions of these eight proteins are briefly summarized in Table 1. These proteins exhibited diverse functions, including induction of epithelial-to-mesenchymal transition (CALU, FAM3C), regulation of autophagy (CTSB) and cell proliferation (GAL1), immune cell recruitment (PPIA), support of biological development and maintenance (PSAP), modulation of metabolism and redox signalling (SOD1), and promotion of angiogenesis and wound healing (TGM2). These eight protein candidates were predicted to act independently of each other and selected for functional validation *in vivo*.

**Table 1:** Overview of biological functions and supplier details for the eight proteins identified by cross-referencing 2 proteomic screens: Wnt+ CM versus Untreated CM, and regenerative MSC CM versus nonregenerative MSC CM. Information is sourced from UniProt.

Gene symbol	Protein name	Biological functions	Relevance to regeneration	Company name	Catalog #	Size (kDa)
***CALU***	Calumenin (CALU)	Protein folding/sorting	Tumour metastasis; EMT; wound healing; immune response	Novus Biologicals	NBC1-26372	37
***CTSB***	Cathepsin B (CTSB)	Lysosomal proteolytic degradation	Tumour metastasis; apoptosis; autophagy; cell proliferation; remodelling	Abcam	ab283434	36
***FAM3C***	FAM3 metabolism regulating signalling molecule C (FAM3C)	Hepatic glucose and lipid metabolism	Tumour metastasis; epithelial to mesenchymal transition (EMT)	Abcam	ab151637	23
***LGALS1***	Galectin 1 (GAL1)	Cell-cell and cell-matrix interactions	Cell proliferation; cell differentiation; cell migration; apoptosis; inflammation; angiogenesis	Bio-Techne	1152-GA	15
***PPIA***	Peptidyl-prolyl cis-trans isomerase A (PPIA)	Protein folding	Cell signalling and recruitment; transcriptional regulation	Ray Biotech	230-00764	21
***PSAP***	Prosaposin (PSAP)	Lysosomal catabolism of glycosphingolipids	Development and protection of neurons; maintenance of biological systems	Novus Biologicals	H00005660-P01	81

### iPan-injection of the 8-protein combination improved glycemic control

**Figure 2:**
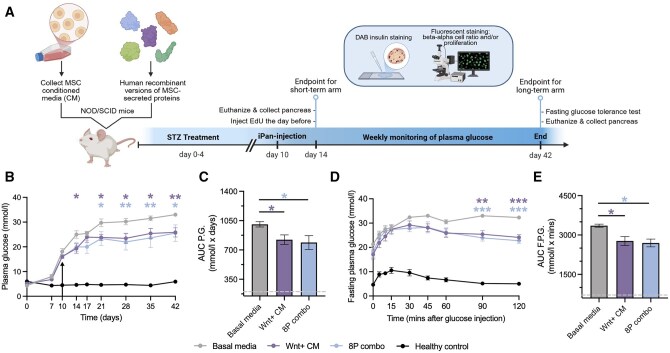
iPan-injection of the 8-protein combination reduced hyperglycemia and improved glucose tolerance. (A) Schematic of *in vivo* experimental design (created using BioRender). Compared to basal media-injected (vehicle) control mice (*n* = 7), (B) mice iPan-injected with Wnt+ CM (*n* = 10) or 8-protein combination (*n* = 7) demonstrated decreased nonfasting plasma glucose levels from day 21 to 42. (C) Area under the curve for plasma glucose was reduced for both treatment groups. (D) Mice that received Wnt+ CM or 8-protein combination demonstrated lower plasma glucose levels at 90- and 120-min following injection of a glucose bolus, and (E) reduced AUC during glucose tolerance tests. Dashed line represents data obtained from healthy controls (*n* = 6). Glucose data was presented as mean ± SEM compared by two-way ANOVA with Dunnett’s multiple comparisons test. AUC was compared using one-way ANOVA with Dunnett’s comparisons test (*P < .05, **P < .01, ***P < .001 vs basal media).

We previously showed that iPan-injection of 4-8μg total protein of Wnt+ CM into STZ-treated NOD/SCID mice reduced hyperglycemia and improved glucose tolerance.^27^ Notably, systemic delivery of up to 80 μg (10-fold higher dose) of protein from Wnt+ CM through IV injection showed no regenerative effect on the pancreas. We therefore used this unique intrapancreatic delivery modality to validate the islet regenerative capacity of the 8-protein combination over 42 days with iPan-injection on day 10 (Figure 2A). There was no difference in NFPG levels among treatment groups prior to iPan-injection on day 10, indicating successful randomization and comparable hyperglycemic progression across groups before treatment (Figure 2B). Compared to basal media-injected (vehicle) controls that showed a continual rise in NFPG levels over 42 days, mice iPan-injected with Wnt+ CM or the 8-protein combination showed a plateau in NFPG levels between days 21 and 42 (Figure 2B). Total NFPG AUCs were equivalent for Wnt+ CM and 8-protein treated mice, with both demonstrating significant improvement relative to basal media controls (Figure 2C). Importantly, analyses of weight change revealed no significant differences between any treatment groups throughout the experimental timeline. Glucose tolerance tests performed on day 42 also demonstrated enhanced glucose clearance in Wnt+ CM and 8-protein combination treatment groups compared to basal media controls (Figure 2D and E). Therefore, iPan-injection of the 8-protein combination improved glucose control to a similar extent as Wnt+ CM, warranting further *in situ* characterization of islet regenerative characteristics. Nonetheless, mice injected with the 8-protein combination remained hyperglycemic compared to healthy controls that did not undergo beta cell ablation.

### iPan-injection of the 8-protein combination increased beta cell mass

**Figure 3:**
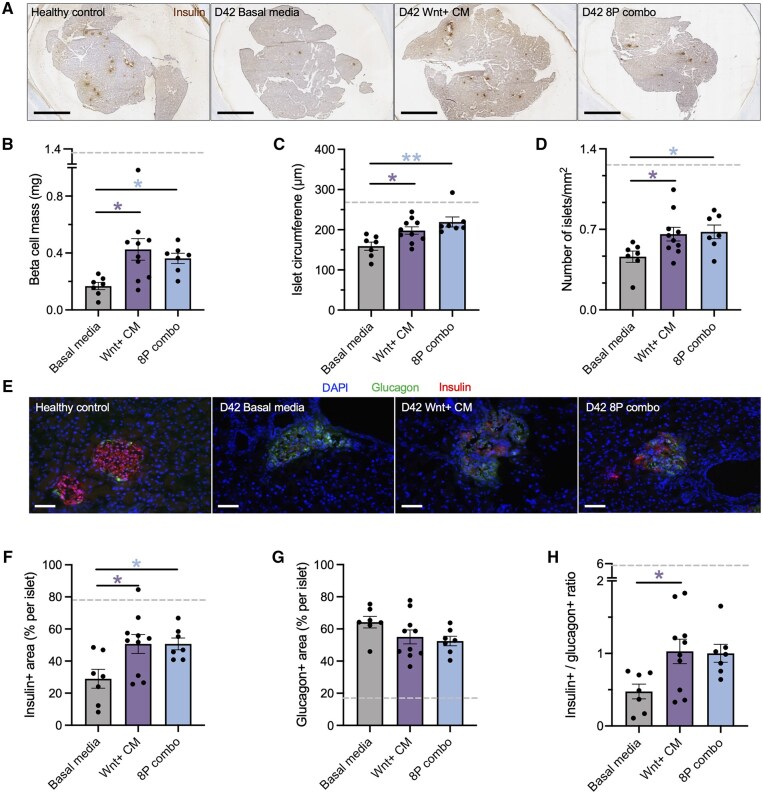
iPan-injection of the 8-protein combination increased beta cell mass and beta cell area per islet. (A) Representative photomicrographs of insulin+ islets on day 42 (scale bar: 2 mm). Compared to basal media-injected controls (*n* = 7), mice iPan-injected with Wnt+ CM (*N* = 4, *n* = 10) or 8-protein combination (*n* = 7) demonstrated (B) increased beta cell mass, (C) increased islet size, and (D) increased islet number. (E) Representative photomicrographs of immunofluorescent detection of insulin+ and glucagon+ islets (scale bar: 50 μm). Compared to basal media-injected controls, (F) mice that received Wnt+ CM or 8-protein combination demonstrated increased beta cell area per islet, (G) with no difference in alpha cell area per islet between groups. (H) Only the Wnt+ CM injected group showed increased beta/alpha cell ratio. Dashed line represents data from healthy controls (*n* = 6). All islets within three sections per mouse, >200 μm apart were quantified. Data presented as mean ± SEM compared by one-way ANOVA followed by Dunnett’s multiple comparisons test (*P < .05, **P < .01 vs basal media).

We characterized islet regeneration *in situ* using immunohistochemical and immunofluorescent staining for insulin+ and glucagon+ cells in mice euthanized at day 42. Representative pancreas sections stained for insulin are shown in Figure 3A. Quantification of all islets found on three sections per mouse demonstrated that iPan-injection of Wnt+ CM or the 8-protein combination significantly increased beta cell mass, islet size, and islet number compared to basal media-injected controls (Figure 3B-D). Similarly, immunofluorescent staining revealed an increased percentage of insulin+ area per islet, with no change in glucagon+ area, in the Wnt+ CM or 8-protein groups (Figure 3E-H). Taken together, a single iPan-injection of the 8-protein combination produced sustained effects and enable improved glucose control with increased beta cell mass and improved islet cell composition evident one-month post-treatment.

### iPan-injection of the 8-protein combination induced beta cell regeneration within 4 days

**Figure 4:**
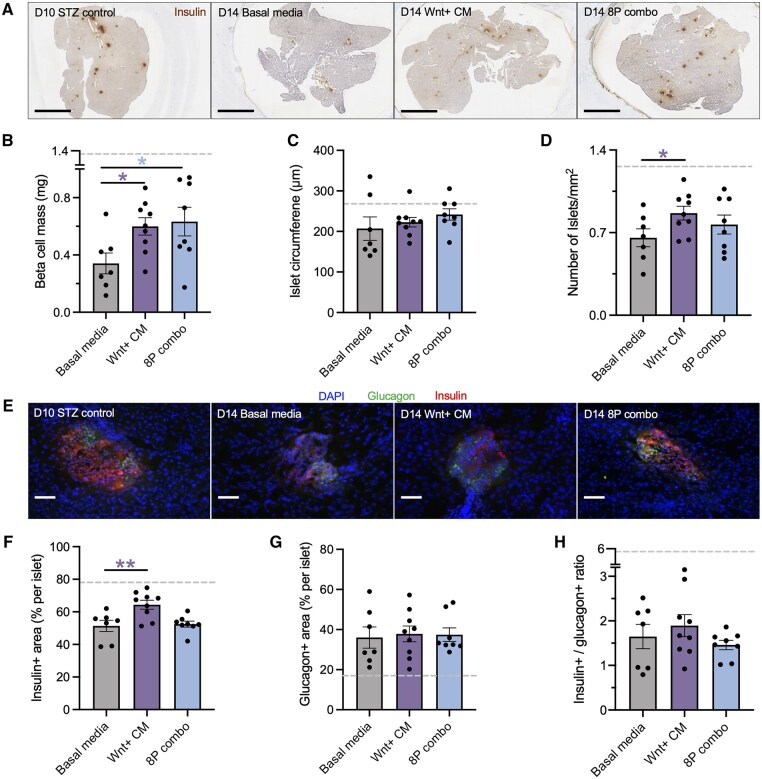
iPan injection of the 8-protein combination increased beta cell mass within 4 days. (A) Representative photomicrographs of insulin+ islets on day 14 (scale bar: 2 mm). Compared to basal media-injected controls at 4 days post-injection (*n* = 6), mice iPan-injected with Wnt+ CM (*N* = 3, *n* = 9) or 8-protein combination (*n* = 8) demonstrated increased (B) beta cell mass, (C) no change in islet size, and (D) increased islet number in the Wnt+ CM group only. (E) Representative fluorescent photomicrographs of insulin+ and glucagon+ islets (scale bar: 50 μm). Compared to basal media-injected controls, mice that received Wnt+ CM demonstrated (F) increased percentage of beta cell area per islet, (G) while there was no difference in the percentage of alpha cell area per islet, and (H) beta/alpha cell ratios were unchanged on day 14. Dashed line represents data obtained from healthy control mice (*n* = 6). All islets within three sections per mouse, >200 μm apart were quantified. Data represent mean ± SEM compared by one-way ANOVA followed by Dunnett’s multiple comparisons test (*P < .05, **P < .01 vs basal media).

We previously demonstrated that iPan-injection of MSC CM rapidly triggered a cascade of regenerative events, including the appearance of proliferating insulin+ clusters adjacent to ducts within 1-4 days post-injection.^27^ More recently, lineage tracing of CK19-expressing cells revealed that injection of Wnt+ CM induced ductal epithelial to beta cell transition followed by beta cell proliferation and maturation.^13^ To capture the early effects of the eight candidate proteins on beta cell regenerative mechanisms, we performed *in situ* analysis of mice euthanized on day 14, 4 days post-injection. Representative insulin-stained and immunofluorescent pancreas sections are shown in Figure 4A and E. Remarkably, as early as 4 days post-injection, mice receiving iPan-injection of the 8-protein combination showed a significant increase in beta cell mass compared to basal media controls, matching the effect of Wnt+ CM (Figure 4B). However, significant increases in islet number and insulin+ area per islet were only observed in mice treated with Wnt+ CM (Figure 4D and F). At 4 days post-injection, no significant differences were detected among groups in islet circumference (Figure 4C), glucagon+ area per islet (Figure 4G) or insulin+/glucagon+ cell ratio (Figure 4H). Collectively, iPan-delivery of Wnt+ CM or the 8-protein combination induced meaningful recovery of beta cell mass within 4 days, suggesting that islet regenerative events were initiated soon after injection.

**Figure 5:**
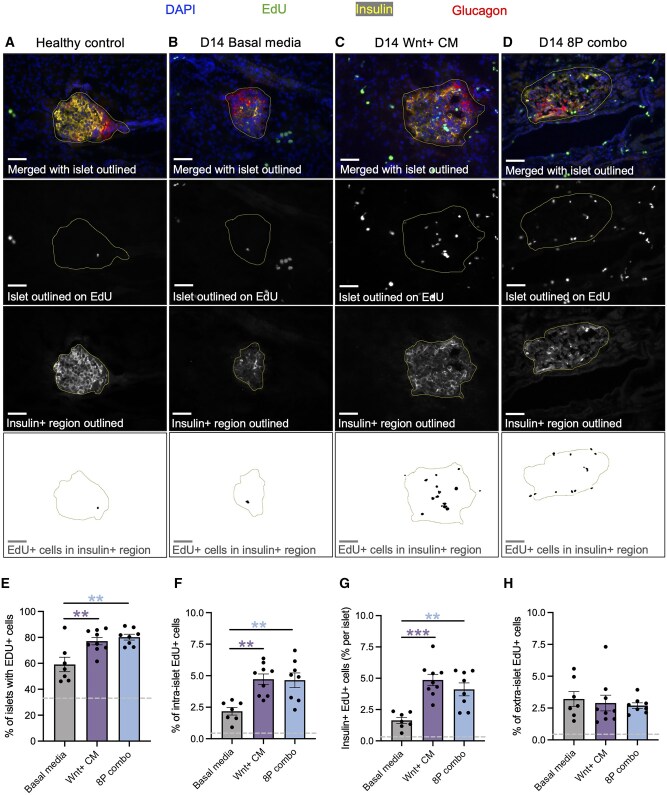
iPan injection of Wnt+ CM or 8-protein combination increased beta cell proliferation. Representative fluorescent photomicrographs (scale bar: 50 μm) showing islets stained for DAPI, EdU, insulin, and glucagon for (A) healthy controls (*n* = 6), mice treated with (B) basal media vehicle control (*n* = 7), (C) Wnt+ CM (*N* = 3, *n* = 9), or (D) 8-protein combination (*n* = 8). Compared to basal media-injected controls, mice iPan-injected with Wnt+ CM or 8-protein combination demonstrated (E) greater proportion of islets containing EdU+ cells, (F) increased percentage of intra-islet EdU+ cells, and (G) increased percentage of insulin+ EdU+ cells per islet at 4 days post injection. There was no difference in the percentage of (H) extra-islet EdU+ cells. Dash lines represent values obtained from healthy controls (*n* = 6). Data represent mean ± SEM. Analyses of significance were performed by ordinary one-way ANOVA followed by Dunnett’s multiple comparisons test (*P < .05, **P < .01, ***P < .001 vs basal media).

Next, we investigated whether increased beta cell mass was accompanied by enhanced beta cell proliferation. In adult islets subjected to STZ, the basal rate of beta cell proliferation is typically minimal.^37^^,^^38^ However, our previous work showed that iPan-injection of Wnt+ CM can enhance beta cell proliferation up to 4-fold between 1 and 4 days.^27^ Building on this, we evaluated whether injection of the 8-protein combination could similarly stimulate endocrine cell proliferation using immunofluorescent staining with EdU. At day 14, beta cell turnover in islets from healthy controls and basal media vehicle control mice was minimal (2%), whereas more robust beta cell proliferation was observed in mice treated with Wnt+ CM or the 8-protein combination (Figure 5A-D). Compared with basal media-injected controls, both treatment groups showed a greater proportion of islets containing proliferating cells (Figure 5E), a higher percentage of intra-islet EdU+ cells (Figure 5F) and increased percentage of insulin+ EdU+ cells per islet (Figure 5G). Interestingly, the proportion of extra-islet EdU+ cells was unchanged among groups (Figure 5H). Similar to Wnt+ CM, iPan-injection of the 8-protein combination enhanced beta cell proliferation within 4 days.

### iPan-injection of single proteins identified CALU and SOD1 as candidates for islet recovery

**Figure 6:**
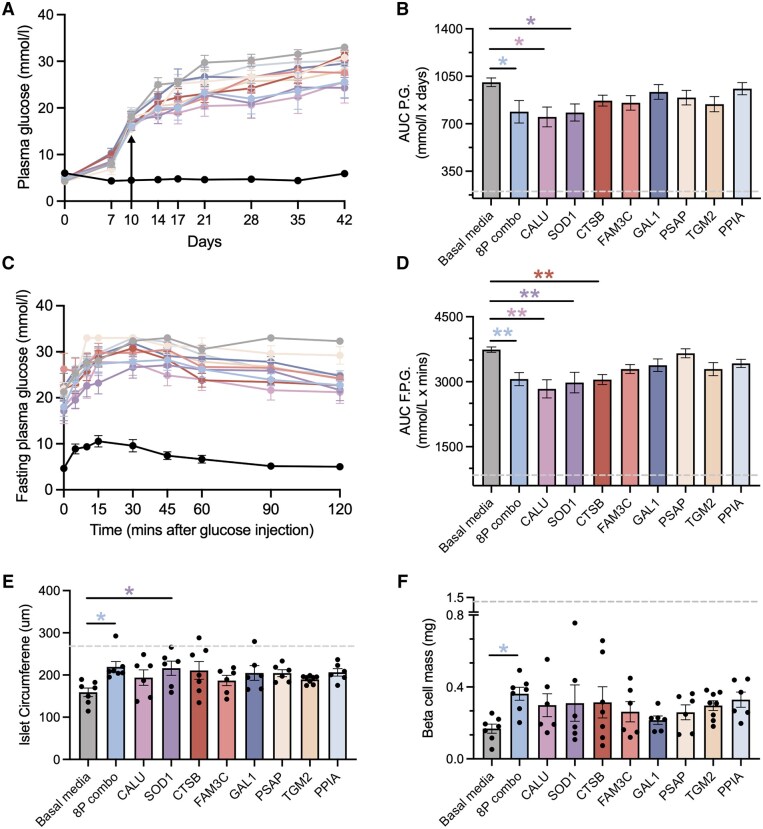
Single protein-injection of CALU or SOD1 improved glucose control. (A) Nonfasting plasma glucose levels measured from days 0-42 in mice iPan-injected with basal media (vehicle control, *n* = 7), 8-protein combination (*n* = 7), or each of the eight proteins individually (CALU, *n* = 6; SOD1, *n* = 6; CTSB, *n* = 7; FAM3C, *n* = 6; GAL1, *n* = 7; PSAP, *n* = 6; TGM2, *n* = 8; and PPIA, *n* = 6). (**B**) Area under the curve for plasma glucose (AUC P.G.) was reduced in mice iPan-injected with 8-protein combination, CALU, and SOD1. (**C**) Fasting plasma glucose was measured for 120 min following intraperitoneal injection of a glucose bolus and (**D**) area under the curve for fasting plasma glucose during the glucose tolerance test was lower in mice that were iPan-injected with 8-protein combination, CALU, SOD1, or CTSB, compared to basal media-injected controls. (**E**) Islet size was increased in mice iPan-injected with 8-protien combination and SOD1, while (**F**) beta cell mass was increased in mice iPan-injected with 8-protein combination, compared to basal media-injected controls. Dashed line represents data obtained from healthy controls (*n* = 6). Data represent mean ± SEM. Glucose data was compared using two-way ANOVA followed by Dunnett’s multiple comparisons test. AUC was compared using one-way ANOVA followed by Dunnett’s multiple comparison test (*P < 0.05, **P < .01 vs basal media).

Given the ability of the 8-protein combination to initiate islet regeneration, we next evaluated each protein individually to identify candidates with the most pronounced potential for functional recovery. Six to eight mice/group were iPan-injected with each candidate protein at 100 ng total dose versus vehicle control. As demonstrated in the schematic (Figure 2A), NFPG levels were monitored over 42 days (Figure 6A and B), glucose tolerance tests (Figure 6C and D) were performed before euthanasia. Islet circumference (Figure 6E) and beta cell mass (Figure 6F) were quantified from pancreas sections post-mortem. Remarkably, single-protein injection of CALU or SOD1 significantly lowered NFPG levels and improved glucose tolerance compared to basal media controls. While iPan-injection of CTSB improved glucose tolerance, it did not alter NFPG levels. However, single-protein injection of FAM3C, GAL1, PSAP, TGM2, and PPIA had no significant effect on NFPG levels or glucose tolerance. Although SOD1 increased islet circumference compared to basal media controls, none of the individually injected proteins significantly increased beta cell mass, suggesting partial regenerative potential. In preliminary studies, injection of 200 ng protein did not show benefit over 100 ng protein. Collectively, these results identified CALU, SOD1, and CTSB as our top protein candidates for functional islet recovery, however, dose optimization is required.

## Discussion

Our study identified and functionally validated the islet regenerative potential of eight MSC-secreted proteins—CALU, CTSB, FAM3C, GAL1, PPIA, PSAP, SOD1, and TGM2—that were uniquely enriched in both Wnt-activated MSC and islet regenerative MSC secretomes.^25^ In alignment with Kuljanin et al.,^27^ iPan-injection of Wnt+ CM into STZ-treated hyperglycemic mice consistently improved glucose control and promoted islet regeneration *in situ*. Notably, our study demonstrated that a single iPan-injection of the defined 8-protein combination stimulated endogenous islet regeneration without the need for cell transfer, as evidenced by sustained reduction of hyperglycemia, improved glucose tolerance, increased beta cell mass, and partial restoration of islet cellular composition. With efficacy comparable to mice iPan-injected with Wnt+ CM, we propose that these eight proteins represented key effectors driving the islet regenerative activity of Wnt+ CM. Notably, the islet regenerative effects of the 8-protein combination were evident as early as 4 days post-injection, demonstrating increased beta cell mass and islet cell proliferation relative to basal media-injected vehicle controls.

In this study, we integrated two independent proteomic datasets for the first time and identified eight candidate proteins with increased expression in both screens. This cross-referenced approach reduced the likelihood of false positives arising from donor-specific variability in MSC samples. Importantly, functional testing was conducted in the standardized STZ-treated NOD/SCID mouse model using the intrapancreatic delivery approach established in our prior studies,^26^^,^^27^ thereby ensuring methodological consistency and enabling direct comparison with earlier work. Furthermore, iPan-injection provided localized exposure of candidate proteins within pancreatic tissue and permitted detection of rapid regenerative responses *in vivo.*^27^^,^^39^ Functional outcomes were evaluated through complementary approaches, including longitudinal glycemic monitoring, glucose tolerance testing, and morphometric analyses of beta cell mass, islet composition, and cell proliferation. The inclusion of both early and late timepoints enabled evaluation of the kinetics and durability of the islet regenerative response.

The present study and Kuljanin et al.^27^ both reported early proliferative responses after Wnt+ CM injection. The magnitude of beta cell proliferation achieved with the 8-protein combination matched that of Wnt+ CM, suggesting that these secreted effectors sufficiently reproduced the proliferative stimulus of the broader MSC secretome. Thus, stimulation of beta cell proliferation represents a contributing mechanism to islet regeneration in this model. Under normal physiological conditions, beta cell turnover in adult murine islets is minimal, and proliferation rates decline markedly after early postnatal life.^38^ Detection of increased beta cell proliferation as early as 4 days post-injection suggests that the administered proteins can overcome this quiescence. However, it remains unclear whether beta cell proliferation represents the driving mechanism of action or whether transient beta cell proliferation occurs as an intermediate step in a broader regenerative process. Lower beta cell mass measured at day 42 compared with day 14 and continued elevation of nonfasted blood glucose levels in basal media injected mice likely reflects ongoing beta cell destruction and remodeling following STZ treatment, as residual cytotoxic and inflammatory effects persist for up to two weeks after administration.^40^^,^^41^ Although progressive beta cell loss is expected in this model, our finding that beta cell mass was increased in both Wnt+ CM and 8-protein combination treatment supports the initiation and persistence of a regenerative effect. In addition, the contribution of CK19-expressing ductal epithelial precursor cells in MSC CM-induced islet regeneration has recently been established using lineage tracing.^13^ While the precise mechanisms underlying MSC CM-induced islet regeneration remains multifactorial, our findings lay the groundwork for lineage-tracing studies aimed at defining the regenerative pathways mediated by the eight proteins identified in this study.

Comparative assessment of individual proteins identified CALU, SOD1, and CTSB as the most effective individual candidates for promoting functional islet recovery or protection from further damage. A single injection of CALU or SOD1 was sufficient to lower nonfasting glucose levels and improve glucose tolerance but not increase beta cell mass, indicating the proteins may require synergistic activity of other proteins in the cocktail to maximize beta cell mass recovery. These data suggest that SOD1 and CALU may enhance beta cell secretory efficiency, nominating them as candidates for therapeutic development. Future preclinical studies of these effectors may also yield novel biologics to support transplanted beta cell survival, engraftment, or functional maturation in islet replacement strategies via the Edmonton protocol,^42^^,^^43^ or pluripotent stem cell-derived islet grafts.^10^

SOD1 is an antioxidant enzyme that catalyzes the dismutation of superoxide radicals, thereby protecting beta cells from oxidative stress.^44^ In animal models, SOD1 overexpression lowered glucose levels and supported hypertrophied beta cell survival.^45^ In contrast, SOD1 deficiency resulted in hyperglycemia and impaired beta cell function.^46^ Clinical studies have similarly reported downregulation of SOD1 gene expression in individuals with T2D.^47^ Based on current evidence, SOD1 secreted by MSC may also influence islet regenerative pathways via modulation of PDX1 expression required for insulin production.^48^ Alternatively, the role of CALU in diabetes models is less well defined. CALU is widely expressed across many tissues and is involved in calcium homeostasis, protein folding, and secretory cargo sorting. It has also been implicated in epithelial-mesenchymal transition (EMT), a process relevant to islet regeneration given its involvement in islet cell plasticity and ductal epithelial hyperplasia.^13^^,^^49^^,^^50^ Finally, CTSB, a lysosomal cysteine protease involved in autophagy and protein turnover, was a third candidate showing functional benefit in glucose homeostasis. CTSB plays a physiological role in the conversion of proinsulin to insulin, and overexpression has been associated with improved insulin sensitivity and enhanced beta cell function.^51^ Together, these findings highlight mechanisms by which SOD1, CALU, and CTSB may contribute to islet protection or enhance insulin secretion, nominating them as promising targets for further investigation and translational drug development.

Several important limitations should be acknowledged, and additional questions remain to be addressed in future studies. First, the intrapancreatic pharmacokinetics of these proteins remain unknown, including their persistence post-injection. Recombinant proteins have a short half-life *in vivo*, where they are expected to be metabolized within hours. Therefore, we hypothesize that a transient signal altered islet phenotype by stimulating a cascade of protection, regenerative or maturation events, without the proteins being retained in the pancreatic niche long-term. Herein, only a single intrapancreatic dose of Wnt+ CM or the defined 8-protein combination was used to evaluate intrinsic regenerative capacity. Given that ongoing beta cell ablation can persist for up to 10-14 days following STZ administration,^40^^,^^41^ along with the continuous remodeling and survival demands of regenerating islets, sustained delivery technologies such as implantable osmotic pumps^52^^,^^53^ or encapsulation^54^ may enable controlled protein delivery. Thus, technology development to optimize dosing frequency and duration for maximal therapeutic efficacy with minimal procedural burden is now required. Second, due to the breadth of *in vivo* experiments, protein dosing was restricted to a single concentration (100 ng/protein), which may not represent the optimal therapeutic dose for individual proteins or the 8-protein combination. Dose optimization of individual proteins, as well as combinations of proteins, is necessary. While the intrapancreatic route is effective in a preclinical context, and showed benefit over 10-fold higher dose IV administration of Wnt+ CM, it is not easily translatable to clinical application due to its invasive nature, risk of procedure-related complications such as bleeding or pancreatitis and need for specialized surgical or endoscopic expertise.^55^ However, clinical methods for injecting into the pancreas are becoming more accessible. Endoscopic Ultrasound-guided fine-needle injection is now used clinically by guiding through the stomach or duodenal wall directly into pancreatic tissue or tumor to locally deliver xenobiotic or cellular agents.^56^ Intra-arterial infusion, where a catheter is threaded through the arterial system to the vessels supplying the pancreas, is also used to administer chemotherapy locally and has been shown as an effective delivery method for autologous stem cells in Type 1 diabetics.^57^ Endoscopic retrograde cholangiopancreatography is also a potential administration route into the pancreas. While our findings suggest that SOD1 and CALU may enhance beta cell secretory efficiency, the precise mechanisms underlying the noted effects remain to be elucidated. We have two lineage tracing mouse models that can be used to assess pancreas cell lineage contribution to regenerating islets. CK19-CreERT Rosa26-mTomato mice,^13^ in which CK19+ ductal cells express TdTomato, can be used to screen for islet neogenic regenerative mechanisms following injection of CM or candidate proteins. In addition, Gluagon-iCre Rosa26-mTomato mice,^58^ in which alpha cells express TdTomato, can be used to assess alpha-to-beta cell transition as another potential mechanism of regeneration following iPan injection of candidate proteins. Additionally, pancreas digestion for single cell flow cytometry analysis, or single-cell transcriptomic profiling may be used in future studies to identify mechanisms contributing to observed improvement in beta cell function. Furthermore, therapeutic potential of the identified proteins has yet to be determined in autoimmune microenvironments. Studies in NOD mice, which demonstrate insulitis similar to human T1D, are underway to assess protein-mediated islet regenerative efficacy in the presence of ongoing autoimmunity. Finally, while this work focused on proteins upregulated under islet regenerative conditions, proteins that were downregulated may also play a role by alleviating inhibitory signals. Future proteomic and functional studies should address this possibility.

Overall, this study provides proof-of-concept that MSC-secreted proteins can be formulated into defined, off-the-shelf biotherapeutics capable of stimulating endogenous islet regeneration. The implications are significant for both beta cell preservation in early T1D and for enhancing the efficacy of islet replacement therapies. These identified effectors act through multifactorial mechanisms, supporting the potential to design biologic combinations that target distinct stages of islet regeneration. From a clinical and policy standpoint, protein-based therapies offer logistical and economic advantages over cell-based treatments. Recombinant protein production is standardized, scalable, and compatible with existing GMP infrastructure.^28^ These therapies could be administered adjunctively with beta cell replacement, immunotherapies, or metabolic modulators to enhance outcomes in the future. Collectively, these findings establish a strong rationale for advancing protein-based regenerative strategies for diabetes and underscore the need for further mechanistic and preclinical evaluation to facilitate clinical translation.

## Supplementary Material

szag022_Supplementary_Data

## Data Availability

The data underlying this article will be shared on reasonable request to the corresponding author.
