# Supplementary material for: Discovery and validation of islet regenerative proteins secreted by human multipotent stromal cells
Source: Stem Cells Transl Med. 2026 Apr 29;15(5):szag022. doi: 10.1093/stcltm/szag022 (PMC13124281; doi:10.1093/stcltm/szag022)
Supplement: szag022_Supplementary_Data [file szag022_supplementary_data.zip › Xie et al_SCTM_SupTables.docx]

**Supplemental Table 1.** Summary of antibodies, reagents, suppliers, and experimental concentrations for immunohistochemical and immunofluorescent staining.

| **Antibody/Reagent** | **Company** | **Catalog #** | **Concentration** |
| --- | --- | --- | --- |
| Monoclonal mouse anti-insulin | Sigma-Aldrich | I2018 | 1/333 |
| Peroxidase labelled horse anti-mouse IgG | MJS Biolynx | VECTSK4105 | 1/250 |
| Monoclonal mouse anti-glucagon | Abcam | AB10988 | 1/500 |
| Monoclonal rabbit anti-insulin | Abcam | AB181547 | 1/1000 |
| Fluorescein labelled horse anti-mouse IgG | MJS Biolynx | VECTFI2000 | 1/200 |
| Texas Red labelled goat anti-rabbit IgG | MJS Biolynx | VECTTI1000 | 1/200 |
| Texas Red labelled horse anti-mouse IgG | MJS Biolynx | VECTTI2000 | 1/200 |
| Cy5 labelled goat anti-rabbit IgG | Thermo Fisher | A10523 | 1/200 |
| DAPI solution | Thermo Fisher | 62248 | 1/1000 |
| Click-iT™ EdU Cell Proliferation Kit for Imaging, Alexa Fluor™ 488 dye | Thermo Fisher | C10337 | Per manufacturer's instructions |

**Supplemental Table 2. 453 proteins were increased in Wnt+ CM by ≥1.5-fold (Log2Fold change≥0.6, *p<0.05).**

| **Gene symbol** | **Protein name** | **Log2Fold change** | **Fold change** | **P-value** |
| --- | --- | --- | --- | --- |
| AP3B1 | AP-3 complex subunit beta-1 | 3.78788 | 13.81228402 | 0.000029 |
| RAB10 | Ras-related protein Rab-10 | 3.69632 | 12.96293053 | 0.035906 |
| SLC3A2 | 4F2 cell-surface antigen heavy chain | 3.693 | 12.9331339 | 0.000777 |
| RPL32 | 60S ribosomal protein L32 | 3.63945 | 12.46188151 | 0.001207 |
| CCT6B | T-complex protein 1 subunit zeta-2 | 3.63683 | 12.43927071 | 0.015915 |
| SELM | Selenoprotein M | 3.61605 | 12.26138462 | 0.000404 |
| PUF60 | Poly(U)-binding-splicing factor PUF60 | 3.58252 | 11.97970104 | 0.009332 |
| COX5B | Cytochrome c oxidase subunit 5B, mitochondrial | 3.40497 | 10.59249102 | 0.021540 |
| ATP5J | ATP synthase-coupling factor 6, mitochondrial | 3.10458 | 8.601450689 | 0.020230 |
| KHSRP | Far upstream element-binding protein 2 | 3.09913 | 8.569018698 | 0.003395 |
| GCSH | Glycine cleavage system H protein, mitochondrial | 3.07149 | 8.406411039 | 0.000272 |
| TNKS1BP1 | 182 kDa tankyrase-1-binding protein | 2.94399 | 7.695366297 | 0.021971 |
| ISG15 | Ubiquitin-like protein ISG15 | 2.8576 | 7.248085631 | 0.006718 |
| PDLIM1 | PDZ and LIM domain protein 1 | 2.80492 | 6.988195649 | 0.028582 |
| CLSTN3 | Calsyntenin-3 | 2.78087 | 6.872666722 | 0.003571 |
| CSTF2 | Cleavage stimulation factor subunit 2 | 2.71822 | 6.580603965 | 0.015229 |
| SH3GL1 | Endophilin-A2 | 2.70348 | 6.513712302 | 0.009920 |
| PDCD6 | Programmed cell death protein 6 | 2.68022 | 6.409536352 | 0.039013 |
| AGFG1 | Arf-GAP domain and FG repeat-containing protein 1 | 2.67425 | 6.383067924 | 0.044576 |
| COX5A | Cytochrome c oxidase subunit 5A, mitochondrial | 2.65721 | 6.308119541 | 0.005013 |
| ABRACL | Costars family protein ABRACL | 2.59428 | 6.038875793 | 0.010424 |
| CNPY3 | Protein canopy homolog 3 | 2.52874 | 5.770674673 | 0.005517 |
| LIF | Leukemia inhibitory factor | 2.5191 | 5.732243917 | 0.011716 |
| SMAP | Small acidic protein | 2.51533 | 5.717284173 | 0.000039 |
| RPL9 | 60S ribosomal protein L9 | 2.48067 | 5.581566191 | 0.000143 |
| FEN1 | Flap endonuclease 1 | 2.41895 | 5.347816637 | 0.041634 |
| ARPP19 | cAMP-regulated phosphoprotein 19 | 2.37923 | 5.202589937 | 0.029651 |
| CRISPLD2 | Cysteine-rich secretory protein LCCL domain-containing 2 | 2.3723 | 5.17765917 | 0.023031 |
| UBE2M | NEDD8-conjugating enzyme Ubc12 | 2.36119 | 5.137939848 | 0.006222 |
| HNRNPU | Heterogeneous nuclear ribonucleoprotein U | 2.3563 | 5.120554334 | 0.009499 |
| HNRNPF | Heterogeneous nuclear ribonucleoprotein F;Heterogeneous nuclear ribonucleoprotein F, N-terminally processed | 2.31207 | 4.96595091 | 0.009942 |
| H2AFV;H2AFZ | Histone H2A.V;Histone H2A.Z | 2.30376 | 4.937428992 | 0.017978 |
| PSMD5 | 26S proteasome non-ATPase regulatory subunit 5 | 2.30117 | 4.928573018 | 0.000299 |
| NDUFS6 | NADH dehydrogenase [ubiquinone] iron-sulfur protein 6, mitochondrial | 2.26767 | 4.81544792 | 0.001718 |
| VDAC2 | Voltage-dependent anion-selective channel protein 2 | 2.26505 | 4.806710783 | 0.019554 |
| RPL3 | 60S ribosomal protein L3 | 2.24427 | 4.737973075 | 0.004180 |
| RBM12 | RNA-binding protein 12 | 2.24169 | 4.729507636 | 0.007581 |
| CCDC6 | Coiled-coil domain-containing protein 6 | 2.20595 | 4.613782516 | 0.005274 |
| TSKU | Tsukushin | 2.19927 | 4.592469054 | 0.000162 |
| CMBL | Carboxymethylenebutenolidase homolog | 2.19162 | 4.568181588 | 0.034417 |
| NNMT | Nicotinamide N-methyltransferase | 2.19017 | 4.563592583 | 0.026315 |
| EIF4E | Eukaryotic translation initiation factor 4E | 2.14626 | 4.426787128 | 0.023627 |
| PPID | Peptidyl-prolyl cis-trans isomerase D | 2.14249 | 4.415234305 | 0.023534 |
| NIT2 | Omega-amidase NIT2 | 2.14017 | 4.408139866 | 0.000319 |
| IFI16 | Gamma-interferon-inducible protein 16 | 2.12814 | 4.371535152 | 0.040593 |
| TOM1 | Target of Myb protein 1 | 2.11394 | 4.328718548 | 0.000969 |
| TCEA1 | Transcription elongation factor A protein 1 | 2.08998 | 4.257421709 | 0.049961 |
| ACOX1 | Peroxisomal acyl-coenzyme A oxidase 1 | 2.08927 | 4.255327 | 0.040285 |
| RPL11 | 60S ribosomal protein L11 | 2.08099 | 4.230974527 | 0.040636 |
| YBX3 | Y-box-binding protein 3 | 2.07895 | 4.224996072 | 0.011228 |
| CANX | Calnexin | 2.07749 | 4.22072256 | 0.044809 |
| LAMP2 | Lysosome-associated membrane glycoprotein 2 | 2.06276 | 4.177847986 | 0.013563 |
| PXN | Paxillin | 2.04772 | 4.134520434 | 0.006555 |
| GRHPR | Glyoxylate reductase/hydroxypyruvate reductase | 2.00997 | 4.027738445 | 0.001433 |
| GMPR2;  GMPR | GMP reductase 2;GMP reductase 1 | 2.00673 | 4.018703112 | 0.020021 |
| PFKP | ATP-dependent 6-phosphofructokinase, platelet type | 2.00597 | 4.01658665 | 0.000448 |
| FKBP3 | Peptidyl-prolyl cis-trans isomerase FKBP3 | 2.00503 | 4.013970461 | 0.041278 |
| STXBP1 | Syntaxin-binding protein 1 | 1.99621 | 3.989505679 | 0.011030 |
| C12orf57 | Protein C10 | 1.9944 | 3.984503598 | 0.013766 |
| SRRT | Serrate RNA effector molecule homolog | 1.97518 | 3.931772917 | 0.015424 |
| HDLBP | Vigilin | 1.95593 | 3.879659395 | 0.003855 |
| CSTB | Cystatin-B | 1.94957 | 3.862593885 | 0.032403 |
| ABCF1 | ATP-binding cassette sub-family F member 1 | 1.9353 | 3.824576486 | 0.012555 |
| FAM195B | Protein FAM195B | 1.92639 | 3.801028915 | 0.037247 |
| RPL23A | 60S ribosomal protein L23a | 1.87073 | 3.657175854 | 0.005763 |
| NOP56 | Nucleolar protein 56 | 1.86761 | 3.649275322 | 0.019250 |
| OXSR1 | Serine/threonine-protein kinase OSR1 | 1.86657 | 3.646645605 | 0.017831 |
| RAB11B;  RAB11A | Ras-related protein Rab-11B;Ras-related protein Rab-11A | 1.85157 | 3.608927097 | 0.028827 |
| UFD1L | Ubiquitin fusion degradation protein 1 homolog | 1.84926 | 3.603153215 | 0.021055 |
| MYOF | Myoferlin | 1.82236 | 3.536592511 | 0.015733 |
| SH3KBP1 | SH3 domain-containing kinase-binding protein 1 | 1.81787 | 3.525602928 | 0.020660 |
| LYPLA1 | Acyl-protein thioesterase 1 | 1.7921 | 3.463186304 | 0.046809 |
| HIST1H2AJ;  HIST1H2AH;  H2AFJ;  HIST2H2AC;  HIST1H2AC;  HIST3H2A;  HIST2H2AA3;  HIST1H2AD;  HIST1H2AG;  HIST1H2AB;  HIST1H2AA;  H2AFX | Histone H2A type 1-J;Histone H2A type 1-H;Histone H2A.J;Histone H2A type 2-C;Histone H2A type 1-C;Histone H2A type 3;Histone H2A type 2-A;Histone H2A type 1-D;Histone H2A type 1;Histone H2A type 1-B/E;Histone H2A type 1-A;Histone H2AX | 1.78863 | 3.454866585 | 0.002783 |
| RANGAP1 | Ran GTPase-activating protein 1 | 1.77566 | 3.423946122 | 0.028219 |
| LPP | Lipoma-preferred partner | 1.77207 | 3.415436572 | 0.010607 |
| TXLNA | Alpha-taxilin | 1.76159 | 3.390716113 | 0.035147 |
| HIST2H3A;  HIST3H3;  H3F3A;  HIST1H3A;  H3F3C | Histone H3.2;Histone H3.1t;Histone H3.3;Histone H3.1;Histone H3.3C | 1.75307 | 3.370750864 | 0.000551 |
| HIST1H4A | Histone H4 | 1.75303 | 3.370657408 | 0.015687 |
| HMGB3 | High mobility group protein B3 | 1.74817 | 3.359321794 | 0.001411 |
| BRK1 | Protein BRICK1 | 1.72717 | 3.310777367 | 0.020078 |
| LRRC59 | Leucine-rich repeat-containing protein 59 | 1.71123 | 3.274398702 | 0.000580 |
| EWSR1 | RNA-binding protein EWS | 1.70367 | 3.2572851 | 0.008547 |
| PSMC3 | 26S protease regulatory subunit 6A | 1.69665 | 3.241473998 | 0.006998 |
| HIST2H2BE;  HIST1H2BB;  HIST1H2BO;  HIST1H2BJ;  HIST3H2BB | Histone H2B type 2-E;Histone H2B type 1-B;Histone H2B type 1-O;Histone H2B type 1-J;Histone H2B type 3-B | 1.69057 | 3.227842086 | 0.003103 |
| CHCHD2 | Coiled-coil-helix-coiled-coil-helix domain-containing protein 2 | 1.68442 | 3.214111549 | 0.034290 |
| DYNC1H1 | Cytoplasmic dynein 1 heavy chain 1 | 1.67497 | 3.193127146 | 0.001914 |
| VTA1 | Vacuolar protein sorting-associated protein VTA1 homolog | 1.67455 | 3.192197692 | 0.041291 |
| LGMN | Legumain | 1.67313 | 3.189057256 | 0.003961 |
| AARS | Alanine--tRNA ligase, cytoplasmic | 1.67313 | 3.189057256 | 0.026355 |
| KHDRBS1 | KH domain-containing, RNA-binding, signal transduction-associated protein 1 | 1.66637 | 3.174149325 | 0.003741 |
| EIF5 | Eukaryotic translation initiation factor 5 | 1.65503 | 3.149297393 | 0.011786 |
| REXO2 | Oligoribonuclease, mitochondrial | 1.62427 | 3.082861336 | 0.000440 |
| GORASP2 | Golgi reassembly-stacking protein 2 | 1.61367 | 3.060293452 | 0.049902 |
| NQO1 | NAD(P)H dehydrogenase [quinone] 1 | 1.61333 | 3.059572318 | 0.000645 |
| ENY2 | Transcription and mRNA export factor ENY2 | 1.61045 | 3.053470696 | 0.027438 |
| GCLM | Glutamate--cysteine ligase regulatory subunit | 1.60437 | 3.040629426 | 0.042992 |
| STAU1 | Double-stranded RNA-binding protein Staufen homolog 1 | 1.60424 | 3.04035545 | 0.013407 |
| WDR44 | WD repeat-containing protein 44 | 1.60046 | 3.032399853 | 0.019388 |
| DR1 | Protein Dr1 | 1.5619 | 2.952424158 | 0.006780 |
| DTYMK | Thymidylate kinase | 1.55791 | 2.944270045 | 0.033981 |
| RPL7A | 60S ribosomal protein L7a | 1.54013 | 2.908207079 | 0.005547 |
| NDUFA8 | NADH dehydrogenase [ubiquinone] 1 alpha subcomplex subunit 8 | 1.53851 | 2.90494329 | 0.030632 |
| SCP2 | Non-specific lipid-transfer protein | 1.53467 | 2.897221527 | 0.038036 |
| ARF4 | ADP-ribosylation factor 4 | 1.53213 | 2.892125184 | 0.006406 |
| TCEB2 | Transcription elongation factor B polypeptide 2 | 1.52353 | 2.874936319 | 0.002757 |
| RPS28 | 40S ribosomal protein S28 | 1.51753 | 2.863004623 | 0.018841 |
| CHMP1B | Charged multivesicular body protein 1b | 1.49962 | 2.827682227 | 0.020576 |
| PDCD5 | Programmed cell death protein 5 | 1.49577 | 2.820146287 | 0.014633 |
| RPS25 | 40S ribosomal protein S25 | 1.4956 | 2.819813994 | 0.000758 |
| CLIP1 | CAP-Gly domain-containing linker protein 1 | 1.48587 | 2.800860247 | 0.001244 |
| ACTB | Actin, cytoplasmic 1;Actin, cytoplasmic 1, N-terminally processed | 1.48463 | 2.798453935 | 0.000637 |
| PYCARD | Apoptosis-associated speck-like protein containing a CARD | 1.48037 | 2.790202829 | 0.005002 |
| TGFB2 | Transforming growth factor beta-2;Latency-associated peptide | 1.47817 | 2.785951225 | 0.008966 |
| LRRFIP2 | Leucine-rich repeat flightless-interacting protein 2 | 1.47415 | 2.778199112 | 0.047938 |
| RPL27A | 60S ribosomal protein L27a | 1.4712 | 2.772524098 | 0.006068 |
| S100A11 | Protein S100-A11;Protein S100-A11, N-terminally processed | 1.46263 | 2.756103373 | 0.026840 |
| HSP90AB4P | Putative heat shock protein HSP 90-beta 4 | 1.44973 | 2.731569253 | 0.004987 |
| MATR3 | Matrin-3 | 1.42857 | 2.69179772 | 0.000621 |
| ATP5A1 | ATP synthase subunit alpha, mitochondrial | 1.42093 | 2.677580594 | 0.008745 |
| TMED10 | Transmembrane emp24 domain-containing protein 10 | 1.40113 | 2.641083657 | 0.040524 |
| GLRX5 | Glutaredoxin-related protein 5, mitochondrial | 1.38226 | 2.606764043 | 0.030954 |
| UBQLN2 | Ubiquilin-2 | 1.37573 | 2.594991836 | 0.003545 |
| CCAR2 | Cell cycle and apoptosis regulator protein 2 | 1.36726 | 2.579801387 | 0.032004 |
| SERBP1 | Plasminogen activator inhibitor 1 RNA-binding protein | 1.36593 | 2.577424201 | 0.030810 |
| EIF2S2 | Eukaryotic translation initiation factor 2 subunit 2 | 1.35963 | 2.566193573 | 0.031876 |
| EIF5B | Eukaryotic translation initiation factor 5B | 1.34953 | 2.548290939 | 0.030319 |
| RPS24 | 40S ribosomal protein S24 | 1.34943 | 2.548114311 | 0.003008 |
| BCAT1 | Branched-chain-amino-acid aminotransferase, cytosolic | 1.34773 | 2.545113509 | 0.031125 |
| RPL18 | 60S ribosomal protein L18 | 1.34253 | 2.535956502 | 0.006116 |
| RPL38 | 60S ribosomal protein L38 | 1.33697 | 2.526201992 | 0.033426 |
| CCDC50 | Coiled-coil domain-containing protein 50 | 1.33573 | 2.524031648 | 0.046594 |
| RAN | GTP-binding nuclear protein Ran | 1.32687 | 2.508578353 | 0.000175 |
| AHSA1 | Activator of 90 kDa heat shock protein ATPase homolog 1 | 1.32683 | 2.508508801 | 0.046684 |
| HMGA1 | High mobility group protein HMG-I/HMG-Y | 1.32377 | 2.503193817 | 0.001664 |
| RPS5 | 40S ribosomal protein S5;40S ribosomal protein S5, N-terminally processed | 1.31337 | 2.485213851 | 0.004248 |
| RPL23 | 60S ribosomal protein L23 | 1.308 | 2.475980582 | 0.006854 |
| DNASE2 | Deoxyribonuclease-2-alpha | 1.30653 | 2.473459025 | 0.020291 |
| PSMF1 | Proteasome inhibitor PI31 subunit | 1.30153 | 2.464901507 | 0.027229 |
| S100A6 | Protein S100-A6 | 1.29913 | 2.46080442 | 0.012489 |
| DDX42 | ATP-dependent RNA helicase DDX42 | 1.29784 | 2.458605051 | 0.041691 |
| HNRNPA0 | Heterogeneous nuclear ribonucleoprotein A0 | 1.296 | 2.455471368 | 0.016709 |
| CRIP2 | Cysteine-rich protein 2 | 1.29033 | 2.445839949 | 0.001337 |
| RPS13 | 40S ribosomal protein S13 | 1.28137 | 2.430696891 | 0.000448 |
| SERBP1 | Plasminogen activator inhibitor 1 RNA-binding protein | 1.27873 | 2.426253005 | 0.004973 |
| RPL4 | 60S ribosomal protein L4 | 1.27433 | 2.418864576 | 0.003546 |
| VDAC1 | Voltage-dependent anion-selective channel protein 1 | 1.27135 | 2.413873378 | 0.003782 |
| BUB3 | Mitotic checkpoint protein BUB3 | 1.27081 | 2.412970035 | 0.006060 |
| CRKL | Crk-like protein | 1.25927 | 2.393745873 | 0.016121 |
| CNBP | Cellular nucleic acid-binding protein | 1.2592 | 2.393629731 | 0.000902 |
| RBMX;  RBMXL1 | RNA-binding motif protein, X chromosome;RNA-binding motif protein, X chromosome, N-terminally processed;RNA binding motif protein, X-linked-like-1 | 1.25127 | 2.380508863 | 0.000978 |
| RPS7 | 40S ribosomal protein S7 | 1.24967 | 2.377870257 | 0.000531 |
| RPL18A | 60S ribosomal protein L18a | 1.24513 | 2.370399127 | 0.009109 |
| RPS14 | 40S ribosomal protein S14 | 1.2405 | 2.362804066 | 0.001188 |
| SSB | Lupus La protein | 1.2374 | 2.357732427 | 0.003016 |
| DUT | Deoxyuridine 5-triphosphate nucleotidohydrolase, mitochondrial | 1.22853 | 2.343281051 | 0.001152 |
| RPS15 | 40S ribosomal protein S15 | 1.22607 | 2.339288828 | 0.001476 |
| HMGB1;  HMGB1P1 | High mobility group protein B1;Putative high mobility group protein B1-like 1 | 1.22573 | 2.338737593 | 0.004388 |
| DAZAP1 | DAZ-associated protein 1 | 1.22443 | 2.336631126 | 0.009331 |
| HSBP1 | Heat shock factor-binding protein 1 | 1.2178 | 2.32591762 | 0.001491 |
| RANBP1 | Ran-specific GTPase-activating protein | 1.21747 | 2.325385654 | 0.022061 |
| TES | Testin | 1.21667 | 2.324096544 | 0.045961 |
| CLIC1 | Chloride intracellular channel protein 1 | 1.2166 | 2.323983781 | 0.003586 |
| AP3D1 | AP-3 complex subunit delta-1 | 1.2135 | 2.318995467 | 0.006058 |
| FAM3C | Protein FAM3C | 1.2133 | 2.318674008 | 0.001963 |
| S100A13 | Protein S100-A13 | 1.2113 | 2.315461871 | 0.009307 |
| YKT6 | Synaptobrevin homolog YKT6 | 1.2066 | 2.307930852 | 0.020647 |
| PHF5A | PHD finger-like domain-containing protein 5A | 1.20397 | 2.30372738 | 0.034717 |
| RPS8 | 40S ribosomal protein S8 | 1.2025 | 2.301381246 | 0.002043 |
| RPL6 | 60S ribosomal protein L6 | 1.20153 | 2.299834426 | 0.005993 |
| SRI | Sorcin | 1.19153 | 2.28394831 | 0.005843 |
| GAPDH | Glyceraldehyde-3-phosphate dehydrogenase | 1.18553 | 2.27446936 | 0.002549 |
| RAD23B | UV excision repair protein RAD23 homolog B | 1.18303 | 2.270531418 | 0.003528 |
| FUBP1 | Far upstream element-binding protein 1 | 1.1813 | 2.267810354 | 0.003306 |
| ALYREF | THO complex subunit 4 | 1.1792 | 2.26451171 | 0.022692 |
| U2AF2 | Splicing factor U2AF 65 kDa subunit | 1.1742 | 2.256677095 | 0.002011 |
| ZYX | Zyxin | 1.17123 | 2.252036172 | 0.007179 |
| EDF1 | Endothelial differentiation-related factor 1 | 1.17067 | 2.251162186 | 0.004609 |
| ARF1;  ARF3 | ADP-ribosylation factor 1;ADP-ribosylation factor 3 | 1.1658 | 2.243575914 | 0.013137 |
| MMP14 | Matrix metalloproteinase-14 | 1.16573 | 2.243467057 | 0.011818 |
| RPS16 | 40S ribosomal protein S16 | 1.16363 | 2.240203822 | 0.001389 |
| RPS19 | 40S ribosomal protein S19 | 1.15127 | 2.221093305 | 0.002109 |
| GABARAPL2 | Gamma-aminobutyric acid receptor-associated protein-like 2 | 1.15031 | 2.219615834 | 0.028455 |
| TXN | Thioredoxin | 1.14747 | 2.215250734 | 0.000047 |
| BANF1 | Barrier-to-autointegration factor;Barrier-to-autointegration factor, N-terminally processed | 1.14547 | 2.212181872 | 0.000799 |
| ARL3 | ADP-ribosylation factor-like protein 3 | 1.14477 | 2.211108775 | 0.004571 |
| RPS4X;  RPS4Y2 | 40S ribosomal protein S4, X isoform;40S ribosomal protein S4, Y isoform 2 | 1.14463 | 2.210894218 | 0.000323 |
| CHORDC1 | Cysteine and histidine-rich domain-containing protein 1 | 1.14147 | 2.206056896 | 0.015962 |
| RPS15A | 40S ribosomal protein S15a | 1.1404 | 2.204421342 | 0.012580 |
| RPL8 | 60S ribosomal protein L8 | 1.13947 | 2.203000771 | 0.002005 |
| PCBP2;  PCBP3 | Poly(rC)-binding protein 2;Poly(rC)-binding protein 3 | 1.13773 | 2.200345386 | 0.006965 |
| LDLR | Low-density lipoprotein receptor | 1.13587 | 2.19751041 | 0.001313 |
| TUBA1A;  TUBA3C;  TUBA3E | Tubulin alpha-1A chain;Tubulin alpha-3C/D chain;Tubulin alpha-3E chain | 1.1357 | 2.197251482 | 0.004938 |
| PDAP1 | 28 kDa heat- and acid-stable phosphoprotein | 1.1329 | 2.192991165 | 0.015895 |
| AK2 | Adenylate kinase 2, mitochondrial;Adenylate kinase 2, mitochondrial, N-terminally processed | 1.13033 | 2.189088074 | 0.030065 |
|  |  | 1.1297 | 2.188132346 | 0.015974 |
| SKP1 | S-phase kinase-associated protein 1 | 1.12597 | 2.18248237 | 0.002929 |
| TUBB3 | Tubulin beta-3 chain | 1.12593 | 2.18242186 | 0.002774 |
| RAB7A | Ras-related protein Rab-7a | 1.12347 | 2.178703691 | 0.002774 |
| AHNAK | Neuroblast differentiation-associated protein AHNAK | 1.1164 | 2.168052962 | 0.014236 |
| RPS20 | 40S ribosomal protein S20 | 1.11633 | 2.16794777 | 0.005017 |
| CCDC58 | Coiled-coil domain-containing protein 58 | 1.11437 | 2.165004465 | 0.020213 |
| AK1 | Adenylate kinase isoenzyme 1 | 1.1116 | 2.160851606 | 0.001688 |
| ERH | Enhancer of rudimentary homolog | 1.11007 | 2.158561205 | 0.001219 |
| EIF1AX;  EIF1AY | Eukaryotic translation initiation factor 1A, X-chromosomal;Eukaryotic translation initiation factor 1A, Y-chromosomal | 1.10763 | 2.15491356 | 0.006976 |
| TMOD3 | Tropomodulin-3 | 1.1071 | 2.15412206 | 0.008963 |
| MOB1A;  MOB1B | MOB kinase activator 1A;MOB kinase activator 1B | 1.10237 | 2.14707115 | 0.012715 |
| BPNT1 | 3(2),5-bisphosphate nucleotidase 1 | 1.09267 | 2.132683679 | 0.011129 |
| PFDN5 | Prefoldin subunit 5 | 1.08853 | 2.12657244 | 0.037542 |
| EPS15L1 | Epidermal growth factor receptor substrate 15-like 1 | 1.08759 | 2.125187305 | 0.030899 |
| TMA7 | Translation machinery-associated protein 7 | 1.08503 | 2.121419596 | 0.016510 |
| UFM1 | Ubiquitin-fold modifier 1 | 1.08457 | 2.120743294 | 0.000880 |
| RPS18 | 40S ribosomal protein S18 | 1.07407 | 2.10536446 | 0.002584 |
| RPL14 | 60S ribosomal protein L14 | 1.0712 | 2.101180353 | 0.001857 |
| NCL | Nucleolin | 1.0705 | 2.100161102 | 0.002173 |
| GLRX3 | Glutaredoxin-3 | 1.06963 | 2.098895006 | 0.002325 |
| EIF1;  EIF1B | Eukaryotic translation initiation factor 1;Eukaryotic translation initiation factor 1b | 1.06853 | 2.097295289 | 0.020704 |
| DNAJC9 | DnaJ homolog subfamily C member 9 | 1.06783 | 2.096277922 | 0.000098 |
| PCBP1 | Poly(rC)-binding protein 1 | 1.06697 | 2.095028689 | 0.014176 |
| HSPA9 | Stress-70 protein, mitochondrial | 1.06573 | 2.09322878 | 0.010928 |
| SEC22B | Vesicle-trafficking protein SEC22b | 1.06513 | 2.092358412 | 0.001333 |
| SARS | Serine--tRNA ligase, cytoplasmic | 1.064 | 2.0907202 | 0.023718 |
| RPS21 | 40S ribosomal protein S21 | 1.06067 | 2.085900007 | 0.018810 |
| UBE2V1 | Ubiquitin-conjugating enzyme E2 variant 1 | 1.05827 | 2.082432886 | 0.013733 |
| RPL24 | 60S ribosomal protein L24 | 1.05793 | 2.081942176 | 0.005031 |
| SBDS | Ribosome maturation protein SBDS | 1.05677 | 2.080268862 | 0.005773 |
| IP | Hepatoma-derived growth factor | 1.056 | 2.07915887 | 0.004185 |
| UBE2N | Ubiquitin-conjugating enzyme E2 N | 1.04223 | 2.05940846 | 0.002186 |
| RPS2 | 40S ribosomal protein S2 | 1.04107 | 2.057753256 | 0.000780 |
| HIST1H1B | Histone H1.5 | 1.03887 | 2.054617731 | 0.004704 |
| RPL5 | 60S ribosomal protein L5 | 1.0365 | 2.05124526 | 0.004615 |
| GNPTG | N-acetylglucosamine-1-phosphotransferase subunit gamma | 1.03647 | 2.051202606 | 0.024495 |
| STRAP | Serine-threonine kinase receptor-associated protein | 1.03257 | 2.045665131 | 0.048096 |
| OLFML2B | Olfactomedin-like protein 2B | 1.0316 | 2.044290185 | 0.020496 |
| YBX1 | Nuclease-sensitive element-binding protein 1 | 1.03033 | 2.042491395 | 0.000063 |
| SH3BGRL3 | SH3 domain-binding glutamic acid-rich-like protein 3 | 1.0281 | 2.039336717 | 0.015157 |
| LIMA1 | LIM domain and actin-binding protein 1 | 1.02573 | 2.035989329 | 0.038784 |
| TAGLN | Transgelin | 1.02293 | 2.032041688 | 0.001537 |
| PSAP | Prosaposin;Saposin-A;Saposin-B-Val;Saposin-B;Saposin-C;Saposin-D | 1.0197 | 2.02749731 | 0.013163 |
| CHMP2B | Charged multivesicular body protein 2b | 1.01651 | 2.023019183 | 0.007882 |
| RPS3A | 40S ribosomal protein S3a | 1.01567 | 2.021841636 | 0.001087 |
| HNRNPH1 | Heterogeneous nuclear ribonucleoprotein H;Heterogeneous nuclear ribonucleoprotein H, N-terminally processed | 1.01553 | 2.021645444 | 0.008411 |
| DYNLL1 | Dynein light chain 1, cytoplasmic | 1.00963 | 2.01339467 | 0.010495 |
| GNB2L1 | Guanine nucleotide-binding protein subunit beta-2-like 1;Guanine nucleotide-binding protein subunit beta-2-like 1, N-terminally processed | 1.00953 | 2.013255117 | 0.001925 |
| BTF3L4 | Transcription factor BTF3 homolog 4 | 1.00867 | 2.01205536 | 0.027518 |
| STIP1 | Stress-induced-phosphoprotein 1 | 1.00697 | 2.00968585 | 0.007596 |
| DYNLRB1 | Dynein light chain roadblock-type 1 | 1.00597 | 2.008293325 | 0.013883 |
| EIF3G | Eukaryotic translation initiation factor 3 subunit G | 1.0055 | 2.007639171 | 0.009228 |
| CSRP1 | Cysteine and glycine-rich protein 1 | 1.0034 | 2.004718959 | 0.018185 |
| HMOX1 | Heme oxygenase 1 | 1.0008 | 2.001109343 | 0.003096 |
| SGTA | Small glutamine-rich tetratricopeptide repeat-containing protein alpha | 1.0005 | 2.000693267 | 0.006615 |
| RTN4 | Reticulon-4 | 1.00007 | 2.000097043 | 0.026857 |
| CFL2 | Cofilin-2 | 0.999299 | 1.999028444 | 0.008904 |
| SEC13 | Protein SEC13 homolog | 0.9982 | 1.997506226 | 0.011458 |
| RPS10 | 40S ribosomal protein S10 | 0.995767 | 1.994140416 | 0.030001 |
| HSPE1 | 10 kDa heat shock protein, mitochondrial | 0.994233 | 1.992021198 | 0.007225 |
| PPP1CA | Serine/threonine-protein phosphatase PP1-alpha catalytic subunit | 0.993033 | 1.99036497 | 0.005407 |
| RPS9 | 40S ribosomal protein S9 | 0.991266 | 1.987928681 | 0.000652 |
| PPM1G | Protein phosphatase 1G | 0.9887 | 1.984396063 | 0.002929 |
| CTSC | Dipeptidyl peptidase 1;Dipeptidyl peptidase 1 exclusion domain chain;Dipeptidyl peptidase 1 heavy chain;Dipeptidyl peptidase 1 light chain | 0.988033 | 1.983478831 | 0.014797 |
| MTPN | Myotrophin | 0.9831 | 1.976708313 | 0.009659 |
| FAT1 | Protocadherin Fat 1;Protocadherin Fat 1, nuclear form | 0.979 | 1.971098674 | 0.030361 |
| FKBP4 | Peptidyl-prolyl cis-trans isomerase FKBP4;Peptidyl-prolyl cis-trans isomerase FKBP4, N-terminally processed | 0.977934 | 1.969642777 | 0.002935 |
| SFPQ | Splicing factor, proline- and glutamine-rich | 0.977701 | 1.969324699 | 0.001692 |
| NPM1 | Nucleophosmin | 0.976467 | 1.96764097 | 0.000247 |
| CACYBP | Calcyclin-binding protein | 0.973867 | 1.964098115 | 0.000276 |
| RPS6 | 40S ribosomal protein S6 | 0.972599 | 1.962372606 | 0.012417 |
| CHMP4B | Charged multivesicular body protein 4b | 0.972033 | 1.961602877 | 0.029168 |
| RPSA | 40S ribosomal protein SA | 0.9695 | 1.95816183 | 0.005057 |
| DDX1 | ATP-dependent RNA helicase DDX1 | 0.9689 | 1.957347623 | 0.000825 |
| RPL7 | 60S ribosomal protein L7 | 0.968701 | 1.957077652 | 0.000551 |
| RPS26;RPS26P11 | 40S ribosomal protein S26;Putative 40S ribosomal protein S26-like 1 | 0.9668 | 1.954500563 | 0.000231 |
| UGDH | UDP-glucose 6-dehydrogenase | 0.966101 | 1.953553817 | 0.001459 |
| FAHD1 | Acylpyruvase FAHD1, mitochondrial | 0.965033 | 1.952108173 | 0.004866 |
| TCEAL3;  TCEAL6 | Transcription elongation factor A protein-like 3;Transcription elongation factor A protein-like 6 | 0.964867 | 1.951883572 | 0.031473 |
| SOD1 | Superoxide dismutase [Cu-Zn] | 0.964699 | 1.951656291 | 0.011010 |
| ANP32A | Acidic leucine-rich nuclear phosphoprotein 32 family member A | 0.963633 | 1.950214755 | 0.004524 |
| CDV3 | Protein CDV3 homolog | 0.963367 | 1.949855213 | 0.010019 |
| PFDN1 | Prefoldin subunit 1 | 0.9615 | 1.947333526 | 0.034173 |
| UBXN1 | UBX domain-containing protein 1 | 0.960766 | 1.946343033 | 0.009716 |
| HMGN1 | Non-histone chromosomal protein HMG-14 | 0.9607 | 1.946253994 | 0.011434 |
| FABP5 | Fatty acid-binding protein, epidermal | 0.960433 | 1.945893834 | 0.002433 |
| PDLIM4 | PDZ and LIM domain protein 4 | 0.958766 | 1.943646698 | 0.032536 |
| IST1 | IST1 homolog | 0.9567 | 1.940865306 | 0.008590 |
| PDXK | Pyridoxal kinase | 0.954533 | 1.937952218 | 0.010026 |
| HN1 | Hematological and neurological expressed 1 protein;Hematological and neurological expressed 1 protein, N-terminally processed | 0.950667 | 1.932766025 | 0.027963 |
| CYCS | Cytochrome c | 0.950066 | 1.931961039 | 0.004090 |
| UBE2L3 | Ubiquitin-conjugating enzyme E2 L3 | 0.9496 | 1.931337103 | 0.001681 |
| C14orf166 | UPF0568 protein C14orf166 | 0.9489 | 1.93040024 | 0.031400 |
| NASP | Nuclear autoantigenic sperm protein | 0.9485 | 1.929865094 | 0.003967 |
| PPIH | Peptidyl-prolyl cis-trans isomerase H | 0.944134 | 1.924033609 | 0.017302 |
| CALD1 | Caldesmon | 0.943967 | 1.923810904 | 0.013273 |
| LASP1 | LIM and SH3 domain protein 1 | 0.941267 | 1.920213864 | 0.018566 |
| RPS3 | 40S ribosomal protein S3 | 0.936667 | 1.914101056 | 0.003920 |
| EFHD2 | EF-hand domain-containing protein D2 | 0.935467 | 1.912509614 | 0.004407 |
| RPS12 | 40S ribosomal protein S12 | 0.935433 | 1.912464542 | 0.025289 |
| EIF4B | Eukaryotic translation initiation factor 4B | 0.935401 | 1.912422123 | 0.005600 |
| BASP1 | Brain acid soluble protein 1 | 0.934266 | 1.91091817 | 0.017899 |
| STMN1 | Stathmin | 0.933102 | 1.909377018 | 0.005648 |
| ST13;  ST13P5 | Hsc70-interacting protein;Putative protein FAM10A5 | 0.9305 | 1.905936429 | 0.011703 |
| SUMO4 | Small ubiquitin-related modifier 4 | 0.9289 | 1.903823849 | 0.006610 |
| SEC31A | Protein transport protein Sec31A | 0.928834 | 1.903736756 | 0.007273 |
| CNN2 | Calponin-2 | 0.928367 | 1.903120616 | 0.000889 |
| PSME1 | Proteasome activator complex subunit 1 | 0.9241 | 1.89750015 | 0.017087 |
| PCNP | PEST proteolytic signal-containing nuclear protein | 0.922334 | 1.895178845 | 0.026410 |
| EEF1G | Elongation factor 1-gamma | 0.917 | 1.888184838 | 0.004409 |
| LSM8 | U6 snRNA-associated Sm-like protein LSm8 | 0.916866 | 1.888009469 | 0.021988 |
| CBR1 | Carbonyl reductase [NADPH] 1 | 0.914334 | 1.884698826 | 0.006130 |
| BAG3 | BAG family molecular chaperone regulator 3 | 0.913701 | 1.883872073 | 0.031172 |
| TAGLN2 | Transgelin-2 | 0.912067 | 1.881739603 | 0.003396 |
| HLA-C | HLA class I histocompatibility antigen, Cw-12 alpha chain;HLA class I histocompatibility antigen, Cw-16 alpha chain;HLA class I histocompatibility antigen, Cw-14 alpha chain | 0.910233 | 1.879348995 | 0.005664 |
| PTBP1 | Polypyrimidine tract-binding protein 1 | 0.9102 | 1.879306008 | 0.018043 |
| TBCB | Tubulin-folding cofactor B | 0.905134 | 1.872718428 | 0.033045 |
| ANP32B | Acidic leucine-rich nuclear phosphoprotein 32 family member B | 0.900934 | 1.867274464 | 0.007626 |
| FHL2 | Four and a half LIM domains protein 2 | 0.899833 | 1.865849988 | 0.013225 |
| MARCKS | Myristoylated alanine-rich C-kinase substrate | 0.899133 | 1.864944892 | 0.007466 |
| RPL28 | 60S ribosomal protein L28 | 0.896967 | 1.862147045 | 0.009679 |
| SUGT1 | Suppressor of G2 allele of SKP1 homolog | 0.895667 | 1.860469836 | 0.033618 |
| MAP4 | Microtubule-associated protein 4 | 0.893666 | 1.857891176 | 0.011879 |
| AAMDC | Mth938 domain-containing protein | 0.889766 | 1.85287557 | 0.034810 |
| PSMD1 | 26S proteasome non-ATPase regulatory subunit 1 | 0.888534 | 1.851293968 | 0.046579 |
| TPM4 | Tropomyosin alpha-4 chain | 0.884134 | 1.845656405 | 0.004109 |
| PTMA | Prothymosin alpha;Prothymosin alpha, N-terminally processed;Thymosin alpha-1 | 0.8838 | 1.845229164 | 0.038956 |
| SH3BGRL | SH3 domain-binding glutamic acid-rich-like protein | 0.878532 | 1.838503598 | 0.005438 |
| MYL6 | Myosin light polypeptide 6 | 0.871667 | 1.829775942 | 0.003869 |
| EEF1B2 | Elongation factor 1-beta | 0.870466 | 1.828253343 | 0.018915 |
| ENO1 | Alpha-enolase | 0.868132 | 1.825297976 | 0.002099 |
| COL8A1 | Collagen alpha-1(VIII) chain;Vastatin | 0.862067 | 1.817640644 | 0.002820 |
| ADI1 | 1,2-dihydroxy-3-keto-5-methylthiopentene dioxygenase | 0.8555 | 1.809385732 | 0.034154 |
| LSM3 | U6 snRNA-associated Sm-like protein LSm3 | 0.853367 | 1.806712562 | 0.008965 |
| CRK | Adapter molecule crk | 0.853132 | 1.806418292 | 0.020905 |
| SMPD1 | Sphingomyelin phosphodiesterase | 0.851366 | 1.804208412 | 0.021395 |
| EEF1A1P5;  EEF1A1 | Putative elongation factor 1-alpha-like 3;Elongation factor 1-alpha 1 | 0.847733 | 1.799670763 | 0.021545 |
| HLA-B | HLA class I histocompatibility antigen, B-42 alpha chain;HLA class I histocompatibility antigen, B-8 alpha chain | 0.847532 | 1.799420046 | 0.013403 |
| DUSP3 | Dual specificity protein phosphatase 3 | 0.847527 | 1.79941381 | 0.028592 |
| ARHGDIA | Rho GDP-dissociation inhibitor 1 | 0.847466 | 1.799337728 | 0.012526 |
| TXNDC12 | Thioredoxin domain-containing protein 12 | 0.846668 | 1.798342733 | 0.016866 |
| SET;  SETSIP | Protein SET;Protein SETSIP | 0.841733 | 1.792201685 | 0.003666 |
| DDX17 | Probable ATP-dependent RNA helicase DDX17 | 0.839833 | 1.789842946 | 0.032415 |
| RAB2A | Ras-related protein Rab-2A | 0.8378 | 1.787322532 | 0.000264 |
| PFDN6 | Prefoldin subunit 6 | 0.836967 | 1.786290845 | 0.038136 |
| EIF4H | Eukaryotic translation initiation factor 4H | 0.834233 | 1.782908914 | 0.009906 |
| ENSA | Alpha-endosulfine | 0.833101 | 1.781510517 | 0.024614 |
| PPIA | Peptidyl-prolyl cis-trans isomerase A;Peptidyl-prolyl cis-trans isomerase A, N-terminally processed | 0.828167 | 1.775428178 | 0.003304 |
| LRRFIP1 | Leucine-rich repeat flightless-interacting protein 1 | 0.8277 | 1.774853566 | 0.002367 |
| USO1 | General vesicular transport factor p115 | 0.825801 | 1.772518887 | 0.033180 |
| MAPRE1 | Microtubule-associated protein RP/EB family member 1 | 0.824834 | 1.771331213 | 0.012926 |
| PGLS | 6-phosphogluconolactonase | 0.821367 | 1.767079564 | 0.038456 |
| TPD52L2 | Tumor protein D54 | 0.818 | 1.762960316 | 0.023146 |
| EIF2S3;  EIF2S3L | Eukaryotic translation initiation factor 2 subunit 3;Putative eukaryotic translation initiation factor 2 subunit 3-like protein | 0.817699 | 1.762592535 | 0.006045 |
| PDLIM5 | PDZ and LIM domain protein 5 | 0.817567 | 1.762431273 | 0.000269 |
| EIF3J | Eukaryotic translation initiation factor 3 subunit J | 0.815133 | 1.759460347 | 0.008331 |
| RPS27A;  UBA52;  UBB;  UBC | Ubiquitin-40S ribosomal protein S27a;Ubiquitin;40S ribosomal protein S27a;Ubiquitin-60S ribosomal protein L40;Ubiquitin;60S ribosomal protein L40;Polyubiquitin-B;Ubiquitin;Polyubiquitin-C;Ubiquitin | 0.812 | 1.755643595 | 0.007609 |
| CAP1 | Adenylyl cyclase-associated protein 1 | 0.809133 | 1.752158152 | 0.027747 |
| DHX9 | ATP-dependent RNA helicase A | 0.809 | 1.75199663 | 0.000601 |
| HNRNPC | Heterogeneous nuclear ribonucleoproteins C1/C2 | 0.807867 | 1.750621265 | 0.008613 |
| NACA | Nascent polypeptide-associated complex subunit alpha;Nascent polypeptide-associated complex subunit alpha, muscle-specific form | 0.806833 | 1.749367019 | 0.005977 |
| TUBB | Tubulin beta chain | 0.805133 | 1.747306866 | 0.010828 |
| SRP9 | Signal recognition particle 9 kDa protein | 0.799867 | 1.740940624 | 0.031681 |
| PPP5C | Serine/threonine-protein phosphatase 5 | 0.796874 | 1.737332631 | 0.032748 |
| PFN1 | Profilin-1 | 0.793299 | 1.733032848 | 0.006539 |
| NSFL1C | NSFL1 cofactor p47 | 0.792066 | 1.731552344 | 0.003916 |
| NAP1L4 | Nucleosome assembly protein 1-like 4 | 0.7907 | 1.729913618 | 0.007026 |
| NAPA | Alpha-soluble NSF attachment protein | 0.787666 | 1.726279418 | 0.001159 |
| EIF2S1 | Eukaryotic translation initiation factor 2 subunit 1 | 0.784234 | 1.722177685 | 0.017319 |
| CAST | Calpastatin | 0.780634 | 1.717885641 | 0.013793 |
| ADH5 | Alcohol dehydrogenase class-3 | 0.780466 | 1.717685607 | 0.010177 |
| NTM | Neurotrimin | 0.776334 | 1.712773049 | 0.023469 |
| ZNF207 | BUB3-interacting and GLEBS motif-containing protein ZNF207 | 0.776 | 1.712376569 | 0.006756 |
| CPPED1 | Serine/threonine-protein phosphatase CPPED1 | 0.771533 | 1.707082757 | 0.018554 |
|  | Galectin-1 | 0.770966 | 1.706411981 | 0.003160 |
| FIS1 | Mitochondrial fission 1 protein | 0.768666 | 1.70369372 | 0.035823 |
| PTPRG | Receptor-type tyrosine-protein phosphatase gamma | 0.768508 | 1.703507147 | 0.028014 |
| RPS17 | 40S ribosomal protein S17 | 0.765967 | 1.700509422 | 0.039257 |
| YWHAE | 14-3-3 protein epsilon | 0.765333 | 1.699762289 | 0.003719 |
| PFN2 | Profilin-2 | 0.7568 | 1.689738505 | 0.007178 |
| PPP1R18 | Phostensin | 0.756533 | 1.689425814 | 0.033692 |
| RPL27 | 60S ribosomal protein L27 | 0.7549 | 1.687514619 | 0.000329 |
| CIRBP | Cold-inducible RNA-binding protein | 0.7549 | 1.687514619 | 0.017113 |
| BOLA2 | BolA-like protein 2 | 0.752701 | 1.684944416 | 0.046628 |
| SYNCRIP | Heterogeneous nuclear ribonucleoprotein Q | 0.751033 | 1.682997461 | 0.008379 |
| SFN | 14-3-3 protein sigma | 0.749033 | 1.680665947 | 0.018182 |
| UBA1 | Ubiquitin-like modifier-activating enzyme 1 | 0.747833 | 1.67926859 | 0.003162 |
| ACTG1 | Actin, cytoplasmic 2;Actin, cytoplasmic 2, N-terminally processed | 0.7472 | 1.678531952 | 0.003990 |
| NUDC | Nuclear migration protein nudC | 0.741667 | 1.672106803 | 0.010553 |
| GNS | N-acetylglucosamine-6-sulfatase | 0.737334 | 1.66709232 | 0.014007 |
| CTSB | Cathepsin B;Cathepsin B light chain;Cathepsin B heavy chain | 0.7344 | 1.66370541 | 0.012825 |
| PSMD9 | 26S proteasome non-ATPase regulatory subunit 9 | 0.733068 | 1.662170066 | 0.024721 |
| YWHAZ | 14-3-3 protein zeta/delta | 0.7286 | 1.657030319 | 0.004020 |
| SPP1 | Osteopontin | 0.727767 | 1.65607384 | 0.008341 |
| YWHAQ | 14-3-3 protein theta | 0.726433 | 1.654543245 | 0.023603 |
| CDC37 | Hsp90 co-chaperone Cdc37;Hsp90 co-chaperone Cdc37, N-terminally processed | 0.723066 | 1.65068633 | 0.014052 |
| PSMD14 | 26S proteasome non-ATPase regulatory subunit 14 | 0.721299 | 1.648665822 | 0.029581 |
| MYL12A;  MYL12B | Myosin regulatory light chain 12A;Myosin regulatory light chain 12B | 0.721266 | 1.648628111 | 0.037802 |
| RBM3 | RNA-binding protein 3 | 0.7203 | 1.647524592 | 0.010447 |
| RPL30 | 60S ribosomal protein L30 | 0.720133 | 1.647333893 | 0.000471 |
| HNRNPL | Heterogeneous nuclear ribonucleoprotein L | 0.7199 | 1.647067865 | 0.019681 |
| CAPRIN1 | Caprin-1 | 0.716967 | 1.643722776 | 0.027192 |
| LRRC47 | Leucine-rich repeat-containing protein 47 | 0.716544 | 1.643240905 | 0.045311 |
| PPA1 | Inorganic pyrophosphatase | 0.715599 | 1.642164895 | 0.015830 |
| PPA2 | Inorganic pyrophosphatase 2, mitochondrial | 0.714966 | 1.641444533 | 0.016350 |
| FKBP1A | Peptidyl-prolyl cis-trans isomerase FKBP1A | 0.714833 | 1.641293218 | 0.013758 |
| HSPB11 | Intraflagellar transport protein 25 homolog | 0.714799 | 1.641254538 | 0.001926 |
| RAB21 | Ras-related protein Rab-21 | 0.713921 | 1.640256002 | 0.004590 |
| SAFB | Scaffold attachment factor B1 | 0.713867 | 1.640194608 | 0.036718 |
| PABPC1;  PABPC3 | Polyadenylate-binding protein 1;Polyadenylate-binding protein 3 | 0.712867 | 1.639058106 | 0.009617 |
| SDC4 | Syndecan-4 | 0.710534 | 1.636409707 | 0.023111 |
| PHPT1 | 14 kDa phosphohistidine phosphatase | 0.709566 | 1.635312099 | 0.045627 |
| PURB | Transcriptional activator protein Pur-beta | 0.709467 | 1.635199885 | 0.022742 |
| DBNL | Drebrin-like protein | 0.704733 | 1.629843001 | 0.007330 |
| COPS4 | COP9 signalosome complex subunit 4 | 0.7038 | 1.628789312 | 0.035852 |
| TGM2 | Protein-glutamine gamma-glutamyltransferase 2 | 0.702833 | 1.627697944 | 0.008974 |
| GNPNAT1 | Glucosamine 6-phosphate N-acetyltransferase | 0.7017 | 1.626420157 | 0.038922 |
| EPRS | Bifunctional glutamate/proline--tRNA ligase;Glutamate--tRNA ligase;Proline--tRNA ligase | 0.700333 | 1.624879801 | 0.041415 |
| HNRNPD | Heterogeneous nuclear ribonucleoprotein D0 | 0.695766 | 1.619744209 | 0.041303 |
| NPC2 | Epididymal secretory protein E1 | 0.694866 | 1.618734075 | 0.045732 |
| RBM8A | RNA-binding protein 8A | 0.694733 | 1.618584853 | 0.048008 |
| HEBP2 | Heme-binding protein 2 | 0.693867 | 1.617613564 | 0.005644 |
| PITPNA | Phosphatidylinositol transfer protein alpha isoform | 0.69386 | 1.617605716 | 0.001179 |
| DDX6 | Probable ATP-dependent RNA helicase DDX6 | 0.693033 | 1.616678717 | 0.020006 |
| HSP90AB1 | Heat shock protein HSP 90-beta | 0.690834 | 1.614216403 | 0.026848 |
| CFL1 | Cofilin-1 | 0.6901 | 1.613395347 | 0.008829 |
| DNAJC8 | DnaJ homolog subfamily C member 8 | 0.6856 | 1.608370745 | 0.036470 |
| GSTO1 | Glutathione S-transferase omega-1 | 0.6847 | 1.607367704 | 0.001215 |
| LMNA | Prelamin-A/C;Lamin-A/C | 0.682433 | 1.604843927 | 0.005611 |
| YWHAB | 14-3-3 protein beta/alpha;14-3-3 protein beta/alpha, N-terminally processed | 0.681167 | 1.603436255 | 0.013082 |
| EIF3E | Eukaryotic translation initiation factor 3 subunit E | 0.6811 | 1.603361792 | 0.047876 |
| EIF5A;  EIF5AL1 | Eukaryotic translation initiation factor 5A-1;Eukaryotic translation initiation factor 5A-1-like | 0.677066 | 1.598884804 | 0.006306 |
| PCNA | Proliferating cell nuclear antigen | 0.675933 | 1.597629636 | 0.020091 |
| TPT1 | Translationally-controlled tumor protein | 0.675067 | 1.596670922 | 0.014227 |
| PCMT1 | Protein-L-isoaspartate(D-aspartate) O-methyltransferase | 0.666234 | 1.586925059 | 0.003094 |
| RPLP2 | 60S acidic ribosomal protein P2 | 0.661535 | 1.581764696 | 0.028435 |
| RAB6A;  RAB6B;  RAB39A | Ras-related protein Rab-6A;Ras-related protein Rab-6B;Ras-related protein Rab-39A | 0.661434 | 1.581653964 | 0.004956 |
| PRKAR1A | cAMP-dependent protein kinase type I-alpha regulatory subunit;cAMP-dependent protein kinase type I-alpha regulatory subunit, N-terminally processed | 0.658667 | 1.578623357 | 0.022140 |
| TBCA | Tubulin-specific chaperone A | 0.6552 | 1.574834256 | 0.027430 |
| ACP1 | Low molecular weight phosphotyrosine protein phosphatase | 0.651566 | 1.570872403 | 0.028431 |
| LRP1 | Prolow-density lipoprotein receptor-related protein 1;Low-density lipoprotein receptor-related protein 1 85 kDa subunit;Low-density lipoprotein receptor-related protein 1 515 kDa subunit;Low-density lipoprotein receptor-related protein 1 intracellular domain | 0.648901 | 1.567973308 | 0.000131 |
| ERP29 | Endoplasmic reticulum resident protein 29 | 0.6468 | 1.565691527 | 0.048035 |
| GLO1 | Lactoylglutathione lyase | 0.646434 | 1.565294374 | 0.040725 |
| RPS11 | 40S ribosomal protein S11 | 0.639233 | 1.557500903 | 0.006848 |
| MRC2 | C-type mannose receptor 2 | 0.636634 | 1.554697608 | 0.004398 |
| PLBD2 | Putative phospholipase B-like 2;Putative phospholipase B-like 2 32 kDa form;Putative phospholipase B-like 2 45 kDa form | 0.6359 | 1.553906825 | 0.049618 |
| CALU | Calumenin | 0.630133 | 1.547707668 | 0.023500 |
| MANF | Mesencephalic astrocyte-derived neurotrophic factor | 0.6277 | 1.545099771 | 0.040740 |
| CAPG | Macrophage-capping protein | 0.624333 | 1.541497981 | 0.039750 |
| TKT | Transketolase | 0.623833 | 1.540963831 | 0.020321 |
| PSME2 | Proteasome activator complex subunit 2 | 0.620166 | 1.537052028 | 0.021015 |
| NAP1L1 | Nucleosome assembly protein 1-like 1 | 0.619233 | 1.536058328 | 0.012472 |
| TPM3 | Tropomyosin alpha-3 chain | 0.6181 | 1.53485248 | 0.007297 |
| PEA15 | Astrocytic phosphoprotein PEA-15 | 0.608201 | 1.524357193 | 0.014923 |
| CASP7 | Caspase 7 | 0.6071 | 1.52319432 | 0.014661 |
| IGF2R | Cation-independent mannose-6-phosphate receptor | 0.6059 | 1.521927885 | 0.005008 |
| PPP2R1A | Serine/threonine-protein phosphatase 2A 65 kDa regulatory subunit A alpha isoform | 0.603366 | 1.519257064 | 0.013635 |
| SRM | Spermidine synthase | 0.602966 | 1.518835895 | 0.001424 |

**Supplemental Table 3.** 62 proteins were increased in Untreated CM by ≥1.5-fold (Log2Fold change≤0.6, *p<0.05).

| **Gene symbol** | **Protein name** | **Log2Fold change** | **Fold change** | **p-value** |
| --- | --- | --- | --- | --- |
| AGRN | Agrin;Agrin N-terminal 110 kDa subunit;Agrin C-terminal 110 kDa subunit;Agrin C-terminal 90 kDa fragment;Agrin C-terminal 22 kDa fragment | -2.6325 | 6.200996177 | 0.014063 |
| SSC5D | Soluble scavenger receptor cysteine-rich domain-containing protein SSC5D | -2.32474 | 5.009754824 | 0.000069 |
| C1QTNF3 | Complement C1q tumor necrosis factor-related protein 3 | -2.28693 | 4.880165248 | 0.039778 |
| FBLN1 | Fibulin-1 | -2.1929 | 4.572236407 | 0.028822 |
| FST | Follistatin | -2.04517 | 4.127219019 | 0.000713 |
| PKP1 | Plakophilin-1 | -1.99096 | 3.975014161 | 0.046450 |
| VWF | von Willebrand factor;von Willebrand antigen 2 | -1.96173 | 3.895288004 | 0.039374 |
| THBS3 | Thrombospondin-3 | -1.7683 | 3.406523124 | 0.024269 |
| DKK1 | Dickkopf-related protein 1 | -1.72917 | 3.315370262 | 0.002113 |
| COPS2 | COP9 signalosome complex subunit 2 | -1.637 | 3.110184144 | 0.030784 |
| ICOSLG | ICOS ligand | -1.58347 | 2.996898037 | 0.007743 |
| ADAMTS5 | A disintegrin and metalloproteinase with thrombospondin motifs 5 | -1.57173 | 2.972609594 | 0.002505 |
| MAP2K1 | Dual specificity mitogen-activated protein kinase kinase 1 | -1.33283 | 2.51896312 | 0.048653 |
| CXCL1 | Growth-regulated alpha protein;GRO-alpha(4-73);GRO-alpha(5-73);GRO-alpha(6-73) | -1.32053 | 2.49757846 | 0.036996 |
| CKM;CKB | Creatine kinase M-type;Creatine kinase M-type, N-terminally processed;Creatine kinase B-type | -1.31245 | 2.483629547 | 0.027547 |
| APMAP | Adipocyte plasma membrane-associated protein | -1.2935 | 2.451220045 | 0.038350 |
| HSPG2 | Basement membrane-specific heparan sulfate proteoglycan core protein;Endorepellin;LG3 peptide | -1.2776 | 2.424353371 | 0.020818 |
| CTGF | Connective tissue growth factor | -1.27723 | 2.423731691 | 0.001903 |
| TNFAIP6 | Tumor necrosis factor-inducible gene 6 protein | -1.27137 | 2.413906841 | 0.005161 |
| PTX3 | Pentraxin-related protein PTX3 | -1.2619 | 2.398113597 | 0.000407 |
| S100A7;S100A7A | Protein S100-A7;Protein S100-A7A | -1.21159 | 2.315927355 | 0.032932 |
| MASP1 | Mannan-binding lectin serine protease 1;Mannan-binding lectin serine protease 1 heavy chain;Mannan-binding lectin serine protease 1 light chain | -1.19943 | 2.296489202 | 0.026894 |
| EPG5 | Ectopic P granules protein 5 homolog | -1.15073 | 2.220262107 | 0.012804 |
| ATP6V1F | V-type proton ATPase subunit F | -1.12775 | 2.185176783 | 0.005966 |
| FAM20C | Extracellular serine/threonine protein kinase FAM20C | -1.0814 | 2.11608855 | 0.003315 |
| PAMR1 | Inactive serine protease PAMR1 | -1.03927 | 2.055187471 | 0.016977 |
| EGFR | Epidermal growth factor receptor | -1.02173 | 2.030352186 | 0.011327 |
| IGFBP4 | Insulin-like growth factor-binding protein 4 | -1.00587 | 2.008154125 | 0.000279 |
| RARRES1 | Retinoic acid receptor responder protein 1 | -0.998167 | 1.997460536 | 0.008823 |
| NID2 | Nidogen-2 | -0.973867 | 1.964098115 | 0.002277 |
| VEGFC | Vascular endothelial growth factor C | -0.953133 | 1.936072529 | 0.000131 |
| PLAU | Urokinase-type plasminogen activator;Urokinase-type plasminogen activator long chain A;Urokinase-type plasminogen activator short chain A;Urokinase-type plasminogen activator chain B | -0.951633 | 1.934060601 | 0.012639 |
| CXCL12 | Stromal cell-derived factor 1;SDF-1-beta(3-72);SDF-1-alpha(3-67) | -0.947533 | 1.92857199 | 0.006962 |
| SERPINF1 | Pigment epithelium-derived factor | -0.9386 | 1.91666739 | 0.017087 |
| COL1A2 | Collagen alpha-2(I) chain | -0.934334 | 1.911008241 | 0.022485 |
| ADAMTS1 | A disintegrin and metalloproteinase with thrombospondin motifs 1 | -0.912999 | 1.882955624 | 0.006625 |
| C1R | Complement C1r subcomponent;Complement C1r subcomponent heavy chain;Complement C1r subcomponent light chain | -0.894667 | 1.859180703 | 0.000602 |
| PAPPA | Pappalysin-1 | -0.8686 | 1.825890186 | 0.022378 |
| COL3A1 | Collagen alpha-1(III) chain | -0.848733 | 1.800918632 | 0.028065 |
| DCN | Decorin | -0.842267 | 1.792865175 | 0.003426 |
| DKK3 | Dickkopf-related protein 3 | -0.834867 | 1.783692595 | 0.005767 |
| COL6A1 | Collagen alpha-1(VI) chain | -0.821301 | 1.766998726 | 0.012321 |
| ANXA2;ANXA2P2 | Annexin A2;Putative annexin A2-like protein | -0.8117 | 1.755278557 | 0.007948 |
| BTD | Biotinidase | -0.809999 | 1.753210227 | 0.009083 |
| GAS6 | Growth arrest-specific protein 6 | -0.798733 | 1.739572732 | 0.032614 |
| GREM1 | Gremlin-1 | -0.763333 | 1.69740755 | 0.000059 |
| PLAT | Tissue-type plasminogen activator;Tissue-type plasminogen activator chain A;Tissue-type plasminogen activator chain B | -0.755067 | 1.687709969 | 0.013992 |
| TIMP2 | Metalloproteinase inhibitor 2 | -0.7425 | 1.673072542 | 0.006959 |
| FBLN1 | Fibulin-1 | -0.736233 | 1.665820556 | 0.027716 |
| TF | Serotransferrin | -0.719699 | 1.646838407 | 0.042409 |
| COL1A1 | Collagen alpha-1(I) chain | -0.713066 | 1.639284207 | 0.022281 |
| C1S | Complement C1s subcomponent;Complement C1s subcomponent heavy chain;Complement C1s subcomponent light chain | -0.707633 | 1.633122487 | 0.034550 |
| SERPING1 | Plasma protease C1 inhibitor | -0.700699 | 1.625292072 | 0.008512 |
| THBS2 | Thrombospondin-2 | -0.687267 | 1.610230253 | 0.034056 |
| ANXA1 | Annexin A1 | -0.6845 | 1.607144891 | 0.011063 |
| LAMB1 | Laminin subunit beta-1 | -0.643499 | 1.562113196 | 0.019528 |
| COL6A2 | Collagen alpha-2(VI) chain | -0.639134 | 1.557394029 | 0.036355 |
| SRPX | Sushi repeat-containing protein SRPX | -0.634567 | 1.552471732 | 0.028024 |
| LUM | Lumican | -0.622334 | 1.539363559 | 0.005990 |
| LAMC1 | Laminin subunit gamma-1 | -0.621967 | 1.538972018 | 0.040519 |
| ADAM9 | Disintegrin and metalloproteinase domain-containing protein 9 | -0.614766 | 1.531309604 | 0.004887 |
| LAMA4 | Laminin subunit alpha-4 | -0.606698 | 1.522769944 | 0.002987 |
