# Supplementary material for: Discovery and validation of islet regenerative proteins secreted by human multipotent stromal cells
Source: Stem Cells Transl Med. 2026 Apr 29;15(5):szag022. doi: 10.1093/stcltm/szag022 (PMC13124281; doi:10.1093/stcltm/szag022)
Supplement: szag022_Supplementary_Data [file szag022_supplementary_data.zip › Xie et al_SCTM_SupMethods.docx]

**Supplementary Methods**

**Generation of MSC CM**

Mononuclear cells (MNC) were isolated from BM aspirates following centrifugation on a Hypaque Ficoll gradient and erythrocyte lysis with ammonium chloride solution (StemCell Technologies). To establish MSC colonies adherent to plastic, MNC were plated at 270,000 cells/cm^2^ in AmnioMAX^TM^ C-100 Complete Media (Thermo Fisher Scientific) that includes a supplement containing fetal bovine serum, gentamicin, and L-glutamine. Human BM-MSC were expanded until 80% confluency at passage 4. AmnioMAX^TM^ C-100 Complete Media was then removed, MSC were washed three times with phosphate-buffered saline to remove residual proteins and growth factors and replaced with supplement-free AmnioMAX^TM^ C-100 Basal Media. BM-MSC were treated with either 1μM CHIR99021 (AbMole Biosciences), a selective GSK3 inhibitor that prevents beta-catenin degradation and activates the canonical Wnt pathway; or dimethyl sulfoxide (DMSO) as vehicle control.

**Mass spectrometry workflow**

Conditioned media (CM) was lyophilized overnight and reconstituted in 8M urea, 50mM ammonium bicarbonate, 10mM dithiothreitol (DTT), and 2% SDS. Protein concentration was quantified using the Pierce 660 nm assay (Thermo Fisher Scientific). Samples were reduced with 10mM DTT and alkylated with 100mM iodoacetamide for 30 min at room temperature in the dark. Briefly, 25μg of protein per sample were precipitated by chloroform/methanol, air-dried, and digested on-pellet with 100μL of 50mM ABC (pH 8.0) containing LysC (Wako) at a 1:100 enzyme-to-protein ratio for 4 hours at 37°C with agitation (1000 RPM), followed by overnight digestion with trypsin/LysC (Promega) at a 1:50 ratio at 37°C in a water bath shaker (400 RPM). A final aliquot of trypsin (1:100) was added for an additional 4 hours before acidifying samples with 10% formic acid (FA, pH 3–4). Approximately 1μg of peptides was injected into a Waters M-Class nanoAcquity HPLC coupled to a Q Exactive Plus Orbitrap mass spectrometer. Samples were trapped on a Symmetry BEH C18 column (5μm, 180μm × 20mm) with 99% Buffer A (0.1% FA in water) and 1% Buffer B (0.1% FA in acetonitrile), then separated on a Peptide BEH C18 column (130 Å, 1.7 μm, 75 μm × 250 mm) using a non-linear gradient at 300nL/min and 35°C. Raw data were processed in MaxQuant (v1.5.8.30) using the Human Uniprot database (20,264 entries), with parameters including up to 3 missed cleavages, carbamidomethylation as a fixed modification, and oxidation, N-terminal acetylation, and deamidation as variable modifications (max 5 per peptide). Mass deviations were set at 20ppm (first search), 4.5ppm (main search), and 20ppm (fragment ions), with a 1% FDR and "match between runs" enabled. Bioinformatics analysis was performed using Perseus (v1.5.8.5), filtering for ≥1 unique peptide in at least 2 of 3 replicates, excluding reverse hits.

**Immunohistochemistry analyses of pancreas tissue**

Antibody concentrations and reagents are detailed in ESM Table 2. Cryopreserved pancreas sections were fixed with 10% buffered formalin for 15 min, followed by blocking with 1% peroxidase block for 5 min and 5% horse serum (MJS Biolynx) for 1 hour. Sections were incubated with mouse anti-insulin primary antibody (Sigma-Aldrich, 1/333) for 1 hour, followed with peroxidase anti-mouse secondary antibody for 30 minutes (MJS Biolynx, 1/250). ImmPACT™ DAB (Vector Laboratories) staining was performed to detect antibody binding, followed by a hematoxylin counterstain (Thermo Fisher Scientific) and slide mounting with VectaMount^TM^ (Vector Laboratories). Slides were scanned for islet quantification at the London Regional Tissue Pathology Facility (Robarts Research Institute, London, ON) using an 20X Aperio AT2 Digital Slide Scanner (Leica Biosystems). Beta cell mass, islet size, and islet number were quantified using Aperio ImageScope software version 12.4.6 (Leica Biosystems) counting all insulin+ regions on 3 different sections per mouse. Beta cell mass, was calculated by: beta cell area $\div$ total section area $\times$ pancreas weight. The circumference of each insulin+ region with > 10 insulin+ cells, , was used to quantity islet circumfe nrence or size. Islet number/mm^2^ of total section area was also quantified by detection of insulin+ regions.

**Immunofluorescent analyses of pancreas tissue**

Cryopreserved pancreas sections were fixed with 10% buffered formalin for 15 minutes, and permeabilized with 1% Triton X-100 (Thermo Fisher Scientific) for 20 minutes, followed by blocking with 5% horse serum (MJS Biolynx) for 1 hour. Sections were incubated with mouse anti-glucagon primary antibody (Abcam, 1/500) for 1 hour, followed with horse anti-mouse fluorescein secondary antibody (MJS Biolynx, 1/200) for 30 minutes while protecting from light. After blocking with 5% goat serum (MJS Biolynx), sections were incubated again with rabbit anti-insulin primary antibody (Abcam, 1/1000) for 1 hour, followed with goat anti-rabbit Texas red secondary antibody (MJS Biolynx, 1/200) for 30 minutes. Tissues were incubated with DAPI solution (Thermo Fisher Scientific) to detect nuclei. Slides were mounted with VectaMount^TM^ (MJS Biolynx). To assess islet cell proliferation, tissue samples were fixed and permeabilized as previously mentioned. Samples were subsequently incubated for 30 minutes in an EdU reaction mixture prepared according to the manufacturer’s instructions for the Click-iT™ EdU Cell Proliferation Kit for Imaging, Alexa Fluor™ 488 dye (Thermo Fisher Scientific). Following the EdU reaction, samples were blocked in horse serum (MJS Biolynx) for 1 hour. Primary staining was performed with mouse anti-mouse glucagon antibody (Abcam, 1/500) for 1 hour. Secondary staining was conducted with horse anti-mouse Texas Red antibody (MJS Biolynx, 1/200) for 30 minutes. After another set of two PBS washes, samples were blocked in goat serum (MJS Biolynx) for 1 hour before incubation with rabbit anti-mouse insulin antibody (Abcam, 1/1000) for 1 hour. Next, goat anti-rabbit Cy5 antibody (Thermo Fisher Scientific, 1/200) was applied for 30 minutes, and samples were stained with DAPI (Thermo Fisher Scientific) for 5 minutes. Final rinsing was performed in water before mounting in VectaMount^TM^ (MJS Biolynx).

To quantify the beta and alpha cell area per islet, the total islet area, insulin+ area and glucagon+ area were manually delineated for all islets identified across three pancreatic sections per mouse. The percentage of beta and alpha cell area was calculated by dividing each respective cell type area by the total islet area. The beta to alpha cell ratio per islet was determined by dividing the measured beta cell area by the corresponding alpha cell area within each islet. Mean value for each variable was then obtained by averaging individual values from all detected islets for each mouse.

Pancreas sections were co-stained with EdU (Thermo Fisher Scientific) to analyze cell proliferation. The number of DAPI+ nuclei was automatically quantified using the StarDist plugin in ImageJ to ensure consistency. For each islet, both the total islet area and the insulin+ region were manually delineated, and the number of EdU+ nuclei within these areas was quantified. To assess islet proliferation, the percentage of islets containing proliferating cells was calculated by dividing the number of islets containing at least one EdU+ cell by the total number of islets for each mouse. Extra-islet proliferation, representing proliferating cells located outside of islet boundaries, was quantified and expressed as a percentage of extra-islet EdU+ cells divided by total DAPI+ nuclei in the surrounding exocrine tissue. Intra-islet proliferation was quantified as the percentage of EdU+ cells within islets relative to the total number of intra-islet DAPI+ nuclei. To specifically assess beta cell proliferation, the number of insulin+ EdU+ double-positive cells was expressed as a percentage of total insulin+ cells within each islet. All quantifications were performed on all islets within the three pancreatic sections per mouse. Mean value for each variable was then obtained by averaging individual values from all detected islets for each mouse.
