# Supplementary material for: Discovery and validation of islet regenerative proteins secreted by human multipotent stromal cells
Source: Stem Cells Transl Med. 2026 Apr 29;15(5):szag022. doi: 10.1093/stcltm/szag022 (PMC13124281; doi:10.1093/stcltm/szag022)
Supplement: szag022_Supplementary_Data [file szag022_supplementary_data.zip › Xie et al_SCTM_SupFiles.docx]

**Supplementary Information**

**Title: Discovery and validation of islet regenerative proteins secreted by human multipotent stromal cells**

**Running Head: Islet regenerative proteins secreted by human MSC**

**Xin Y. Xie^1,2^, Nouran N. Al-Banaa^1,2^, Yina Tian^1,2^, Gillian I. Bell^1,2^, Miljan Kuljanin^3^, Tyler T. Cooper^3^, Caleb J. Podgers^2^, Ajaya Sharma^1,2^, Anargyros Xenocostas^4^, Gilles A. Lajoie^3^, David A. Hess^1,2^.**

**^1^ Department of Physiology and Pharmacology, Schulich School of Medicine and Dentistry, Western University, London, ON, Canada**

**^2^ Molecular Medicine Research Laboratories, Robarts Research Institute, London, ON, Canada**

**^3^ Department of Biochemistry, Western University, London, ON, Canada**

**^4^ Department of Haematology, London Health Sciences Centre, London, ON, Canada**

**Author Contributions:**

Xin Y. Xie: Concept and design, collection and/or assembly of data, data analysis and interpretation, manuscript writing, final approval of manuscript

Nouran N. Al-Banaa: Assembly of data, data analysis and interpretation, manuscript writing, final approval of manuscript.

Yina Tian: Collection and/or assembly of data, data analysis and interpretation, final approval of manuscript

Gillian I. Bell: Concept and design, collection and/or assembly of data, data analysis and interpretation, final approval or manuscript.

Miljan Kuljanin: Concept and design, collection and/or assembly of data, final approval of manuscript.

Tyler T. Cooper: Collection and/or assembly of data, data analysis and interpretation, manuscript writing, final approval of manuscript.

Caleb J. Podgers: Collection of data, final approval of manuscript.

Ajaya Sharma: Collection of data, final approval of manuscript.

Anargyros Xenocostas: Provision of study materials or patients, final approval of manuscript.

Gilles A. Lajoie: Concept and design, financial support, data analysis and interpretation, final approval of manuscript.

David A. Hess: Concept and design, financial support, data analysis and interpretation, manuscript writing, final approval of manuscript.

**Corresponding Author: David A. Hess, Sheldon H. Weinstein Chair in Diabetes Research, Schulich School of Medicine & Dentistry, Professor, Department of Physiology and Pharmacology, University of Western Ontario, Scientist, Robarts Research Institute. 1151 Richmond Street, London, ON, Canada, N6A 5B7. email:** [dhess3@uwo.ca](mailto:dhess3@uwo.ca)

**Disclaimers:** The authors have no conflicts of interest to declare.

**Funding acknowledgement:** This work was funded by a project grant from the Canadian Institutes of Health Research (CIHR MOP# 378189) and by the Sheldon H. Weinstein Chair in Diabetes Research at the Schulich School of Medicine and Dentistry, Western University.

**Keywords:** diabetes mellitus, multipotent stromal cells, stem cells, islet regeneration, proteomics

**Supplementary Methods**

**Generation of MSC CM**

Mononuclear cells (MNC) were isolated from BM aspirates following centrifugation on a Hypaque Ficoll gradient and erythrocyte lysis with ammonium chloride solution (StemCell Technologies). To establish MSC colonies adherent to plastic, MNC were plated at 270,000 cells/cm^2^ in AmnioMAX^TM^ C-100 Complete Media (Thermo Fisher Scientific) that includes a supplement containing fetal bovine serum, gentamicin, and L-glutamine. Human BM-MSC were expanded until 80% confluency at passage 4. AmnioMAX^TM^ C-100 Complete Media was then removed, MSC were washed three times with phosphate-buffered saline to remove residual proteins and growth factors and replaced with supplement-free AmnioMAX^TM^ C-100 Basal Media. BM-MSC were treated with either 1μM CHIR99021 (AbMole Biosciences), a selective GSK3 inhibitor that prevents beta-catenin degradation and activates the canonical Wnt pathway; or dimethyl sulfoxide (DMSO) as vehicle control.

**Mass spectrometry workflow**

Conditioned media (CM) was lyophilized overnight and reconstituted in 8M urea, 50mM ammonium bicarbonate, 10mM dithiothreitol (DTT), and 2% SDS. Protein concentration was quantified using the Pierce 660 nm assay (Thermo Fisher Scientific). Samples were reduced with 10mM DTT and alkylated with 100mM iodoacetamide for 30 min at room temperature in the dark. Briefly, 25μg of protein per sample were precipitated by chloroform/methanol, air-dried, and digested on-pellet with 100μL of 50mM ABC (pH 8.0) containing LysC (Wako) at a 1:100 enzyme-to-protein ratio for 4 hours at 37°C with agitation (1000 RPM), followed by overnight digestion with trypsin/LysC (Promega) at a 1:50 ratio at 37°C in a water bath shaker (400 RPM). A final aliquot of trypsin (1:100) was added for an additional 4 hours before acidifying samples with 10% formic acid (FA, pH 3–4). Approximately 1μg of peptides was injected into a Waters M-Class nanoAcquity HPLC coupled to a Q Exactive Plus Orbitrap mass spectrometer. Samples were trapped on a Symmetry BEH C18 column (5μm, 180μm × 20mm) with 99% Buffer A (0.1% FA in water) and 1% Buffer B (0.1% FA in acetonitrile), then separated on a Peptide BEH C18 column (130 Å, 1.7 μm, 75 μm × 250 mm) using a non-linear gradient at 300nL/min and 35°C. Raw data were processed in MaxQuant (v1.5.8.30) using the Human Uniprot database (20,264 entries), with parameters including up to 3 missed cleavages, carbamidomethylation as a fixed modification, and oxidation, N-terminal acetylation, and deamidation as variable modifications (max 5 per peptide). Mass deviations were set at 20ppm (first search), 4.5ppm (main search), and 20ppm (fragment ions), with a 1% FDR and "match between runs" enabled. Bioinformatics analysis was performed using Perseus (v1.5.8.5), filtering for ≥1 unique peptide in at least 2 of 3 replicates, excluding reverse hits.

**Immunohistochemistry analyses of pancreas tissue**

Antibody concentrations and reagents are detailed in ESM Table 2. Cryopreserved pancreas sections were fixed with 10% buffered formalin for 15 min, followed by blocking with 1% peroxidase block for 5 min and 5% horse serum (MJS Biolynx) for 1 hour. Sections were incubated with mouse anti-insulin primary antibody (Sigma-Aldrich, 1/333) for 1 hour, followed with peroxidase anti-mouse secondary antibody for 30 minutes (MJS Biolynx, 1/250). ImmPACT™ DAB (Vector Laboratories) staining was performed to detect antibody binding, followed by a hematoxylin counterstain (Thermo Fisher Scientific) and slide mounting with VectaMount^TM^ (Vector Laboratories). Slides were scanned for islet quantification at the London Regional Tissue Pathology Facility (Robarts Research Institute, London, ON) using an 20X Aperio AT2 Digital Slide Scanner (Leica Biosystems). Beta cell mass, islet size, and islet number were quantified using Aperio ImageScope software version 12.4.6 (Leica Biosystems) counting all insulin+ regions on 3 different sections per mouse. Beta cell mass, was calculated by: beta cell area $\div$ total section area $\times$ pancreas weight. The circumference of each insulin+ region with > 10 insulin+ cells, , was used to quantity islet circumfe nrence or size. Islet number/mm^2^ of total section area was also quantified by detection of insulin+ regions.

**Immunofluorescent analyses of pancreas tissue**

Cryopreserved pancreas sections were fixed with 10% buffered formalin for 15 minutes, and permeabilized with 1% Triton X-100 (Thermo Fisher Scientific) for 20 minutes, followed by blocking with 5% horse serum (MJS Biolynx) for 1 hour. Sections were incubated with mouse anti-glucagon primary antibody (Abcam, 1/500) for 1 hour, followed with horse anti-mouse fluorescein secondary antibody (MJS Biolynx, 1/200) for 30 minutes while protecting from light. After blocking with 5% goat serum (MJS Biolynx), sections were incubated again with rabbit anti-insulin primary antibody (Abcam, 1/1000) for 1 hour, followed with goat anti-rabbit Texas red secondary antibody (MJS Biolynx, 1/200) for 30 minutes. Tissues were incubated with DAPI solution (Thermo Fisher Scientific) to detect nuclei. Slides were mounted with VectaMount^TM^ (MJS Biolynx). To assess islet cell proliferation, tissue samples were fixed and permeabilized as previously mentioned. Samples were subsequently incubated for 30 minutes in an EdU reaction mixture prepared according to the manufacturer’s instructions for the Click-iT™ EdU Cell Proliferation Kit for Imaging, Alexa Fluor™ 488 dye (Thermo Fisher Scientific). Following the EdU reaction, samples were blocked in horse serum (MJS Biolynx) for 1 hour. Primary staining was performed with mouse anti-mouse glucagon antibody (Abcam, 1/500) for 1 hour. Secondary staining was conducted with horse anti-mouse Texas Red antibody (MJS Biolynx, 1/200) for 30 minutes. After another set of two PBS washes, samples were blocked in goat serum (MJS Biolynx) for 1 hour before incubation with rabbit anti-mouse insulin antibody (Abcam, 1/1000) for 1 hour. Next, goat anti-rabbit Cy5 antibody (Thermo Fisher Scientific, 1/200) was applied for 30 minutes, and samples were stained with DAPI (Thermo Fisher Scientific) for 5 minutes. Final rinsing was performed in water before mounting in VectaMount^TM^ (MJS Biolynx).

To quantify the beta and alpha cell area per islet, the total islet area, insulin+ area and glucagon+ area were manually delineated for all islets identified across three pancreatic sections per mouse. The percentage of beta and alpha cell area was calculated by dividing each respective cell type area by the total islet area. The beta to alpha cell ratio per islet was determined by dividing the measured beta cell area by the corresponding alpha cell area within each islet. Mean value for each variable was then obtained by averaging individual values from all detected islets for each mouse.

Pancreas sections were co-stained with EdU (Thermo Fisher Scientific) to analyze cell proliferation. The number of DAPI+ nuclei was automatically quantified using the StarDist plugin in ImageJ to ensure consistency. For each islet, both the total islet area and the insulin+ region were manually delineated, and the number of EdU+ nuclei within these areas was quantified. To assess islet proliferation, the percentage of islets containing proliferating cells was calculated by dividing the number of islets containing at least one EdU+ cell by the total number of islets for each mouse. Extra-islet proliferation, representing proliferating cells located outside of islet boundaries, was quantified and expressed as a percentage of extra-islet EdU+ cells divided by total DAPI+ nuclei in the surrounding exocrine tissue. Intra-islet proliferation was quantified as the percentage of EdU+ cells within islets relative to the total number of intra-islet DAPI+ nuclei. To specifically assess beta cell proliferation, the number of insulin+ EdU+ double-positive cells was expressed as a percentage of total insulin+ cells within each islet. All quantifications were performed on all islets within the three pancreatic sections per mouse. Mean value for each variable was then obtained by averaging individual values from all detected islets for each mouse.

**Supplemental Fig. 1. Treatment with CHIR99021 consistently elevated intracellular beta-catenin in human BM-MSC.** Intracellular beta-catenin was quantified by flow cytometry in 7 independent BM-MSC samples (N=7). (**a**) Representative flow cytometry plots of beta-catenin mean fluorescence intensity (MFI) in unstained MSC, untreated MSC, and Wnt+ MSC treated with 10 μM CHIR99021. (**b, c**) Wnt+ MSC showed increased intracellular beta-catenin levels compared to untreated MSC (paired Student’s t-test, **p<0.01). Data represent mean ± SEM.


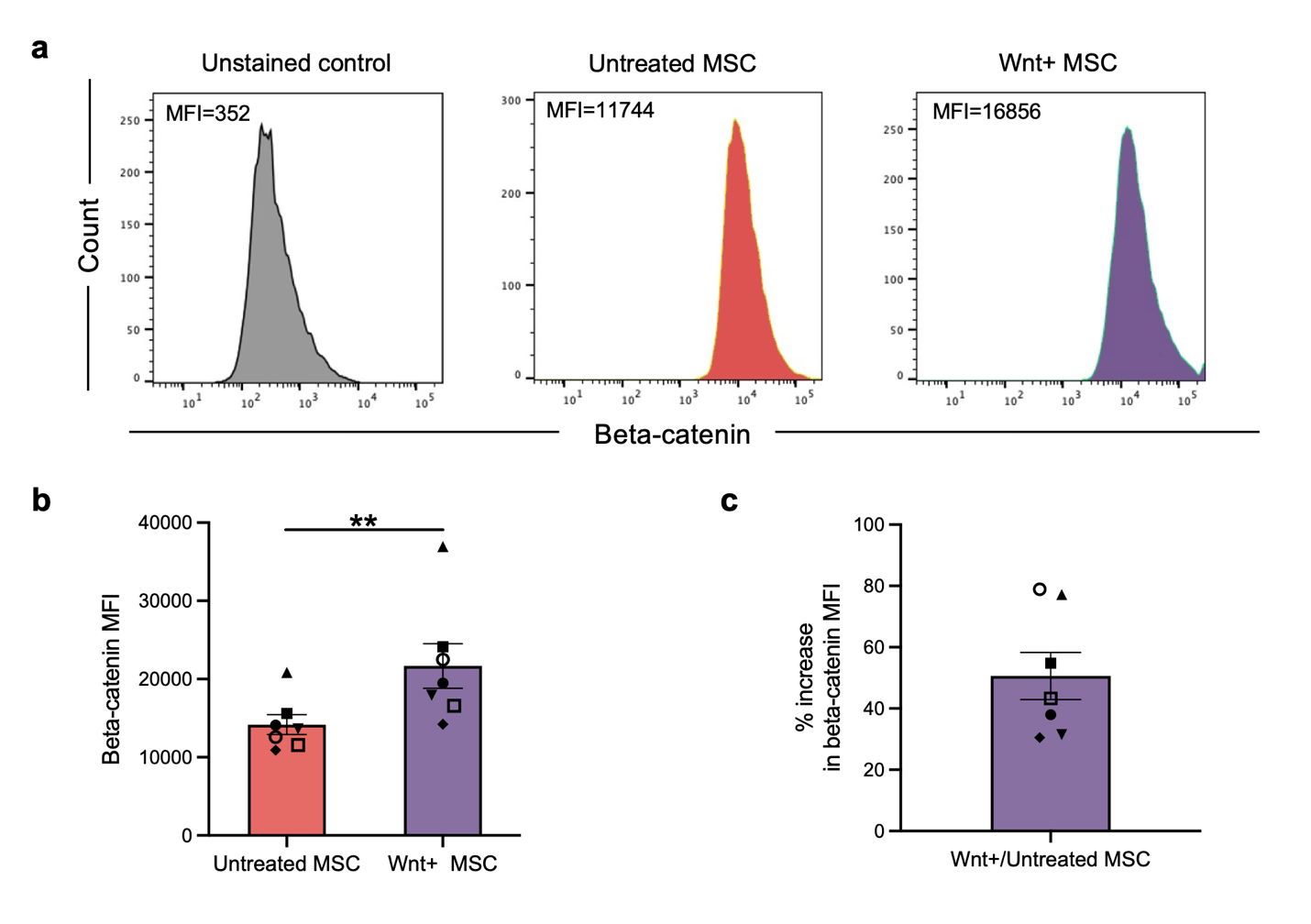


**Supplemental Table 1.** Summary of antibodies, reagents, suppliers, and experimental concentrations for immunohistochemical and immunofluorescent staining.

| **Antibody/Reagent** | **Company** | **Catalog #** | **Concentration** |
| --- | --- | --- | --- |
| Monoclonal mouse anti-insulin | Sigma-Aldrich | I2018 | 1/333 |
| Peroxidase labelled horse anti-mouse IgG | MJS Biolynx | VECTSK4105 | 1/250 |
| Monoclonal mouse anti-glucagon | Abcam | AB10988 | 1/500 |
| Monoclonal rabbit anti-insulin | Abcam | AB181547 | 1/1000 |
| Fluorescein labelled horse anti-mouse IgG | MJS Biolynx | VECTFI2000 | 1/200 |
| Texas Red labelled goat anti-rabbit IgG | MJS Biolynx | VECTTI1000 | 1/200 |
| Texas Red labelled horse anti-mouse IgG | MJS Biolynx | VECTTI2000 | 1/200 |
| Cy5 labelled goat anti-rabbit IgG | Thermo Fisher | A10523 | 1/200 |
| DAPI solution | Thermo Fisher | 62248 | 1/1000 |
| Click-iT™ EdU Cell Proliferation Kit for Imaging, Alexa Fluor™ 488 dye | Thermo Fisher | C10337 | Per manufacturer's instructions |

**Supplemental Table 2. 453 proteins were increased in Wnt+ CM by ≥1.5-fold (Log2Fold change≥0.6, *p<0.05).**

| **Gene symbol** | **Protein name** | **Log2Fold change** | **Fold change** | **P-value** |
| --- | --- | --- | --- | --- |
| *AP3B1* | AP-3 complex subunit beta-1 | 3.78788 | 13.81228402 | 0.000029 |
| *RAB10* | Ras-related protein Rab-10 | 3.69632 | 12.96293053 | 0.035906 |
| *SLC3A2* | 4F2 cell-surface antigen heavy chain | 3.693 | 12.9331339 | 0.000777 |
| *RPL32* | 60S ribosomal protein L32 | 3.63945 | 12.46188151 | 0.001207 |
| *CCT6B* | T-complex protein 1 subunit zeta-2 | 3.63683 | 12.43927071 | 0.015915 |
| *SELM* | Selenoprotein M | 3.61605 | 12.26138462 | 0.000404 |
| *PUF60* | Poly(U)-binding-splicing factor PUF60 | 3.58252 | 11.97970104 | 0.009332 |
| *COX5B* | Cytochrome c oxidase subunit 5B, mitochondrial | 3.40497 | 10.59249102 | 0.021540 |
| *ATP5J* | ATP synthase-coupling factor 6, mitochondrial | 3.10458 | 8.601450689 | 0.020230 |
| *KHSRP* | Far upstream element-binding protein 2 | 3.09913 | 8.569018698 | 0.003395 |
| *GCSH* | Glycine cleavage system H protein, mitochondrial | 3.07149 | 8.406411039 | 0.000272 |
| *TNKS1BP1* | 182 kDa tankyrase-1-binding protein | 2.94399 | 7.695366297 | 0.021971 |
| *ISG15* | Ubiquitin-like protein ISG15 | 2.8576 | 7.248085631 | 0.006718 |
| *PDLIM1* | PDZ and LIM domain protein 1 | 2.80492 | 6.988195649 | 0.028582 |
| *CLSTN3* | Calsyntenin-3 | 2.78087 | 6.872666722 | 0.003571 |
| *CSTF2* | Cleavage stimulation factor subunit 2 | 2.71822 | 6.580603965 | 0.015229 |
| *SH3GL1* | Endophilin-A2 | 2.70348 | 6.513712302 | 0.009920 |
| *PDCD6* | Programmed cell death protein 6 | 2.68022 | 6.409536352 | 0.039013 |
| *AGFG1* | Arf-GAP domain and FG repeat-containing protein 1 | 2.67425 | 6.383067924 | 0.044576 |
| *COX5A* | Cytochrome c oxidase subunit 5A, mitochondrial | 2.65721 | 6.308119541 | 0.005013 |
| *ABRACL* | Costars family protein ABRACL | 2.59428 | 6.038875793 | 0.010424 |
| *CNPY3* | Protein canopy homolog 3 | 2.52874 | 5.770674673 | 0.005517 |
| *LIF* | Leukemia inhibitory factor | 2.5191 | 5.732243917 | 0.011716 |
| *SMAP* | Small acidic protein | 2.51533 | 5.717284173 | 0.000039 |
| *RPL9* | 60S ribosomal protein L9 | 2.48067 | 5.581566191 | 0.000143 |
| *FEN1* | Flap endonuclease 1 | 2.41895 | 5.347816637 | 0.041634 |
| *ARPP19* | cAMP-regulated phosphoprotein 19 | 2.37923 | 5.202589937 | 0.029651 |
| *CRISPLD2* | Cysteine-rich secretory protein LCCL domain-containing 2 | 2.3723 | 5.17765917 | 0.023031 |
| *UBE2M* | NEDD8-conjugating enzyme Ubc12 | 2.36119 | 5.137939848 | 0.006222 |
| *HNRNPU* | Heterogeneous nuclear ribonucleoprotein U | 2.3563 | 5.120554334 | 0.009499 |
| *HNRNPF* | Heterogeneous nuclear ribonucleoprotein F;Heterogeneous nuclear ribonucleoprotein F, N-terminally processed | 2.31207 | 4.96595091 | 0.009942 |
| *H2AFV;H2AFZ* | Histone H2A.V;Histone H2A.Z | 2.30376 | 4.937428992 | 0.017978 |
| *PSMD5* | 26S proteasome non-ATPase regulatory subunit 5 | 2.30117 | 4.928573018 | 0.000299 |
| *NDUFS6* | NADH dehydrogenase [ubiquinone] iron-sulfur protein 6, mitochondrial | 2.26767 | 4.81544792 | 0.001718 |
| *VDAC2* | Voltage-dependent anion-selective channel protein 2 | 2.26505 | 4.806710783 | 0.019554 |
| *RPL3* | 60S ribosomal protein L3 | 2.24427 | 4.737973075 | 0.004180 |
| *RBM12* | RNA-binding protein 12 | 2.24169 | 4.729507636 | 0.007581 |
| *CCDC6* | Coiled-coil domain-containing protein 6 | 2.20595 | 4.613782516 | 0.005274 |
| *TSKU* | Tsukushin | 2.19927 | 4.592469054 | 0.000162 |
| *CMBL* | Carboxymethylenebutenolidase homolog | 2.19162 | 4.568181588 | 0.034417 |
| *NNMT* | Nicotinamide N-methyltransferase | 2.19017 | 4.563592583 | 0.026315 |
| *EIF4E* | Eukaryotic translation initiation factor 4E | 2.14626 | 4.426787128 | 0.023627 |
| *PPID* | Peptidyl-prolyl cis-trans isomerase D | 2.14249 | 4.415234305 | 0.023534 |
| *NIT2* | Omega-amidase NIT2 | 2.14017 | 4.408139866 | 0.000319 |
| *IFI16* | Gamma-interferon-inducible protein 16 | 2.12814 | 4.371535152 | 0.040593 |
| *TOM1* | Target of Myb protein 1 | 2.11394 | 4.328718548 | 0.000969 |
| *TCEA1* | Transcription elongation factor A protein 1 | 2.08998 | 4.257421709 | 0.049961 |
| *ACOX1* | Peroxisomal acyl-coenzyme A oxidase 1 | 2.08927 | 4.255327 | 0.040285 |
| *RPL11* | 60S ribosomal protein L11 | 2.08099 | 4.230974527 | 0.040636 |
| *YBX3* | Y-box-binding protein 3 | 2.07895 | 4.224996072 | 0.011228 |
| *CANX* | Calnexin | 2.07749 | 4.22072256 | 0.044809 |
| *LAMP2* | Lysosome-associated membrane glycoprotein 2 | 2.06276 | 4.177847986 | 0.013563 |
| *PXN* | Paxillin | 2.04772 | 4.134520434 | 0.006555 |
| *GRHPR* | Glyoxylate reductase/hydroxypyruvate reductase | 2.00997 | 4.027738445 | 0.001433 |
| *GMPR2;*  *GMPR* | GMP reductase 2;GMP reductase 1 | 2.00673 | 4.018703112 | 0.020021 |
| *PFKP* | ATP-dependent 6-phosphofructokinase, platelet type | 2.00597 | 4.01658665 | 0.000448 |
| *FKBP3* | Peptidyl-prolyl cis-trans isomerase FKBP3 | 2.00503 | 4.013970461 | 0.041278 |
| *STXBP1* | Syntaxin-binding protein 1 | 1.99621 | 3.989505679 | 0.011030 |
| *C12orf57* | Protein C10 | 1.9944 | 3.984503598 | 0.013766 |
| *SRRT* | Serrate RNA effector molecule homolog | 1.97518 | 3.931772917 | 0.015424 |
| *HDLBP* | Vigilin | 1.95593 | 3.879659395 | 0.003855 |
| *CSTB* | Cystatin-B | 1.94957 | 3.862593885 | 0.032403 |
| *ABCF1* | ATP-binding cassette sub-family F member 1 | 1.9353 | 3.824576486 | 0.012555 |
| *FAM195B* | Protein FAM195B | 1.92639 | 3.801028915 | 0.037247 |
| *RPL23A* | 60S ribosomal protein L23a | 1.87073 | 3.657175854 | 0.005763 |
| *NOP56* | Nucleolar protein 56 | 1.86761 | 3.649275322 | 0.019250 |
| *OXSR1* | Serine/threonine-protein kinase OSR1 | 1.86657 | 3.646645605 | 0.017831 |
| *RAB11B;*  *RAB11A* | Ras-related protein Rab-11B;Ras-related protein Rab-11A | 1.85157 | 3.608927097 | 0.028827 |
| *UFD1L* | Ubiquitin fusion degradation protein 1 homolog | 1.84926 | 3.603153215 | 0.021055 |
| *MYOF* | Myoferlin | 1.82236 | 3.536592511 | 0.015733 |
| *SH3KBP1* | SH3 domain-containing kinase-binding protein 1 | 1.81787 | 3.525602928 | 0.020660 |
| *LYPLA1* | Acyl-protein thioesterase 1 | 1.7921 | 3.463186304 | 0.046809 |
| *HIST1H2AJ;*  *HIST1H2AH;*  *H2AFJ;*  *HIST2H2AC;*  *HIST1H2AC;*  *HIST3H2A;*  *HIST2H2AA3;*  *HIST1H2AD;*  *HIST1H2AG;*  *HIST1H2AB;*  *HIST1H2AA;*  *H2AFX* | Histone H2A type 1-J;Histone H2A type 1-H;Histone H2A.J;Histone H2A type 2-C;Histone H2A type 1-C;Histone H2A type 3;Histone H2A type 2-A;Histone H2A type 1-D;Histone H2A type 1;Histone H2A type 1-B/E;Histone H2A type 1-A;Histone H2AX | 1.78863 | 3.454866585 | 0.002783 |
| *RANGAP1* | Ran GTPase-activating protein 1 | 1.77566 | 3.423946122 | 0.028219 |
| *LPP* | Lipoma-preferred partner | 1.77207 | 3.415436572 | 0.010607 |
| *TXLNA* | Alpha-taxilin | 1.76159 | 3.390716113 | 0.035147 |
| *HIST2H3A;*  *HIST3H3;*  *H3F3A;*  *HIST1H3A;*  *H3F3C* | Histone H3.2;Histone H3.1t;Histone H3.3;Histone H3.1;Histone H3.3C | 1.75307 | 3.370750864 | 0.000551 |
| *HIST1H4A* | Histone H4 | 1.75303 | 3.370657408 | 0.015687 |
| *HMGB3* | High mobility group protein B3 | 1.74817 | 3.359321794 | 0.001411 |
| *BRK1* | Protein BRICK1 | 1.72717 | 3.310777367 | 0.020078 |
| *LRRC59* | Leucine-rich repeat-containing protein 59 | 1.71123 | 3.274398702 | 0.000580 |
| *EWSR1* | RNA-binding protein EWS | 1.70367 | 3.2572851 | 0.008547 |
| *PSMC3* | 26S protease regulatory subunit 6A | 1.69665 | 3.241473998 | 0.006998 |
| *HIST2H2BE;*  *HIST1H2BB;*  *HIST1H2BO;*  *HIST1H2BJ;*  *HIST3H2BB* | Histone H2B type 2-E;Histone H2B type 1-B;Histone H2B type 1-O;Histone H2B type 1-J;Histone H2B type 3-B | 1.69057 | 3.227842086 | 0.003103 |
| *CHCHD2* | Coiled-coil-helix-coiled-coil-helix domain-containing protein 2 | 1.68442 | 3.214111549 | 0.034290 |
| *DYNC1H1* | Cytoplasmic dynein 1 heavy chain 1 | 1.67497 | 3.193127146 | 0.001914 |
| *VTA1* | Vacuolar protein sorting-associated protein VTA1 homolog | 1.67455 | 3.192197692 | 0.041291 |
| *LGMN* | Legumain | 1.67313 | 3.189057256 | 0.003961 |
| *AARS* | Alanine--tRNA ligase, cytoplasmic | 1.67313 | 3.189057256 | 0.026355 |
| *KHDRBS1* | KH domain-containing, RNA-binding, signal transduction-associated protein 1 | 1.66637 | 3.174149325 | 0.003741 |
| *EIF5* | Eukaryotic translation initiation factor 5 | 1.65503 | 3.149297393 | 0.011786 |
| *REXO2* | Oligoribonuclease, mitochondrial | 1.62427 | 3.082861336 | 0.000440 |
| *GORASP2* | Golgi reassembly-stacking protein 2 | 1.61367 | 3.060293452 | 0.049902 |
| *NQO1* | NAD(P)H dehydrogenase [quinone] 1 | 1.61333 | 3.059572318 | 0.000645 |
| *ENY2* | Transcription and mRNA export factor ENY2 | 1.61045 | 3.053470696 | 0.027438 |
| *GCLM* | Glutamate--cysteine ligase regulatory subunit | 1.60437 | 3.040629426 | 0.042992 |
| *STAU1* | Double-stranded RNA-binding protein Staufen homolog 1 | 1.60424 | 3.04035545 | 0.013407 |
| *WDR44* | WD repeat-containing protein 44 | 1.60046 | 3.032399853 | 0.019388 |
| *DR1* | Protein Dr1 | 1.5619 | 2.952424158 | 0.006780 |
| *DTYMK* | Thymidylate kinase | 1.55791 | 2.944270045 | 0.033981 |
| *RPL7A* | 60S ribosomal protein L7a | 1.54013 | 2.908207079 | 0.005547 |
| *NDUFA8* | NADH dehydrogenase [ubiquinone] 1 alpha subcomplex subunit 8 | 1.53851 | 2.90494329 | 0.030632 |
| *SCP2* | Non-specific lipid-transfer protein | 1.53467 | 2.897221527 | 0.038036 |
| *ARF4* | ADP-ribosylation factor 4 | 1.53213 | 2.892125184 | 0.006406 |
| *TCEB2* | Transcription elongation factor B polypeptide 2 | 1.52353 | 2.874936319 | 0.002757 |
| *RPS28* | 40S ribosomal protein S28 | 1.51753 | 2.863004623 | 0.018841 |
| *CHMP1B* | Charged multivesicular body protein 1b | 1.49962 | 2.827682227 | 0.020576 |
| *PDCD5* | Programmed cell death protein 5 | 1.49577 | 2.820146287 | 0.014633 |
| *RPS25* | 40S ribosomal protein S25 | 1.4956 | 2.819813994 | 0.000758 |
| *CLIP1* | CAP-Gly domain-containing linker protein 1 | 1.48587 | 2.800860247 | 0.001244 |
| *ACTB* | Actin, cytoplasmic 1;Actin, cytoplasmic 1, N-terminally processed | 1.48463 | 2.798453935 | 0.000637 |
| *PYCARD* | Apoptosis-associated speck-like protein containing a CARD | 1.48037 | 2.790202829 | 0.005002 |
| *TGFB2* | Transforming growth factor beta-2;Latency-associated peptide | 1.47817 | 2.785951225 | 0.008966 |
| *LRRFIP2* | Leucine-rich repeat flightless-interacting protein 2 | 1.47415 | 2.778199112 | 0.047938 |
| *RPL27A* | 60S ribosomal protein L27a | 1.4712 | 2.772524098 | 0.006068 |
| *S100A11* | Protein S100-A11;Protein S100-A11, N-terminally processed | 1.46263 | 2.756103373 | 0.026840 |
| *HSP90AB4P* | Putative heat shock protein HSP 90-beta 4 | 1.44973 | 2.731569253 | 0.004987 |
| *MATR3* | Matrin-3 | 1.42857 | 2.69179772 | 0.000621 |
| *ATP5A1* | ATP synthase subunit alpha, mitochondrial | 1.42093 | 2.677580594 | 0.008745 |
| *TMED10* | Transmembrane emp24 domain-containing protein 10 | 1.40113 | 2.641083657 | 0.040524 |
| *GLRX5* | Glutaredoxin-related protein 5, mitochondrial | 1.38226 | 2.606764043 | 0.030954 |
| *UBQLN2* | Ubiquilin-2 | 1.37573 | 2.594991836 | 0.003545 |
| *CCAR2* | Cell cycle and apoptosis regulator protein 2 | 1.36726 | 2.579801387 | 0.032004 |
| *SERBP1* | Plasminogen activator inhibitor 1 RNA-binding protein | 1.36593 | 2.577424201 | 0.030810 |
| *EIF2S2* | Eukaryotic translation initiation factor 2 subunit 2 | 1.35963 | 2.566193573 | 0.031876 |
| *EIF5B* | Eukaryotic translation initiation factor 5B | 1.34953 | 2.548290939 | 0.030319 |
| *RPS24* | 40S ribosomal protein S24 | 1.34943 | 2.548114311 | 0.003008 |
| *BCAT1* | Branched-chain-amino-acid aminotransferase, cytosolic | 1.34773 | 2.545113509 | 0.031125 |
| *RPL18* | 60S ribosomal protein L18 | 1.34253 | 2.535956502 | 0.006116 |
| *RPL38* | 60S ribosomal protein L38 | 1.33697 | 2.526201992 | 0.033426 |
| *CCDC50* | Coiled-coil domain-containing protein 50 | 1.33573 | 2.524031648 | 0.046594 |
| *RAN* | GTP-binding nuclear protein Ran | 1.32687 | 2.508578353 | 0.000175 |
| *AHSA1* | Activator of 90 kDa heat shock protein ATPase homolog 1 | 1.32683 | 2.508508801 | 0.046684 |
| *HMGA1* | High mobility group protein HMG-I/HMG-Y | 1.32377 | 2.503193817 | 0.001664 |
| *RPS5* | 40S ribosomal protein S5;40S ribosomal protein S5, N-terminally processed | 1.31337 | 2.485213851 | 0.004248 |
| *RPL23* | 60S ribosomal protein L23 | 1.308 | 2.475980582 | 0.006854 |
| *DNASE2* | Deoxyribonuclease-2-alpha | 1.30653 | 2.473459025 | 0.020291 |
| *PSMF1* | Proteasome inhibitor PI31 subunit | 1.30153 | 2.464901507 | 0.027229 |
| *S100A6* | Protein S100-A6 | 1.29913 | 2.46080442 | 0.012489 |
| *DDX42* | ATP-dependent RNA helicase DDX42 | 1.29784 | 2.458605051 | 0.041691 |
| *HNRNPA0* | Heterogeneous nuclear ribonucleoprotein A0 | 1.296 | 2.455471368 | 0.016709 |
| *CRIP2* | Cysteine-rich protein 2 | 1.29033 | 2.445839949 | 0.001337 |
| *RPS13* | 40S ribosomal protein S13 | 1.28137 | 2.430696891 | 0.000448 |
| *SERBP1* | Plasminogen activator inhibitor 1 RNA-binding protein | 1.27873 | 2.426253005 | 0.004973 |
| *RPL4* | 60S ribosomal protein L4 | 1.27433 | 2.418864576 | 0.003546 |
| *VDAC1* | Voltage-dependent anion-selective channel protein 1 | 1.27135 | 2.413873378 | 0.003782 |
| *BUB3* | Mitotic checkpoint protein BUB3 | 1.27081 | 2.412970035 | 0.006060 |
| *CRKL* | Crk-like protein | 1.25927 | 2.393745873 | 0.016121 |
| *CNBP* | Cellular nucleic acid-binding protein | 1.2592 | 2.393629731 | 0.000902 |
| *RBMX;*  *RBMXL1* | RNA-binding motif protein, X chromosome;RNA-binding motif protein, X chromosome, N-terminally processed;RNA binding motif protein, X-linked-like-1 | 1.25127 | 2.380508863 | 0.000978 |
| *RPS7* | 40S ribosomal protein S7 | 1.24967 | 2.377870257 | 0.000531 |
| *RPL18A* | 60S ribosomal protein L18a | 1.24513 | 2.370399127 | 0.009109 |
| *RPS14* | 40S ribosomal protein S14 | 1.2405 | 2.362804066 | 0.001188 |
| *SSB* | Lupus La protein | 1.2374 | 2.357732427 | 0.003016 |
| *DUT* | Deoxyuridine 5-triphosphate nucleotidohydrolase, mitochondrial | 1.22853 | 2.343281051 | 0.001152 |
| *RPS15* | 40S ribosomal protein S15 | 1.22607 | 2.339288828 | 0.001476 |
| *HMGB1;*  *HMGB1P1* | High mobility group protein B1;Putative high mobility group protein B1-like 1 | 1.22573 | 2.338737593 | 0.004388 |
| *DAZAP1* | DAZ-associated protein 1 | 1.22443 | 2.336631126 | 0.009331 |
| *HSBP1* | Heat shock factor-binding protein 1 | 1.2178 | 2.32591762 | 0.001491 |
| *RANBP1* | Ran-specific GTPase-activating protein | 1.21747 | 2.325385654 | 0.022061 |
| *TES* | Testin | 1.21667 | 2.324096544 | 0.045961 |
| *CLIC1* | Chloride intracellular channel protein 1 | 1.2166 | 2.323983781 | 0.003586 |
| *AP3D1* | AP-3 complex subunit delta-1 | 1.2135 | 2.318995467 | 0.006058 |
| *FAM3C* | Protein FAM3C | 1.2133 | 2.318674008 | 0.001963 |
| *S100A13* | Protein S100-A13 | 1.2113 | 2.315461871 | 0.009307 |
| *YKT6* | Synaptobrevin homolog YKT6 | 1.2066 | 2.307930852 | 0.020647 |
| *PHF5A* | PHD finger-like domain-containing protein 5A | 1.20397 | 2.30372738 | 0.034717 |
| *RPS8* | 40S ribosomal protein S8 | 1.2025 | 2.301381246 | 0.002043 |
| *RPL6* | 60S ribosomal protein L6 | 1.20153 | 2.299834426 | 0.005993 |
| *SRI* | Sorcin | 1.19153 | 2.28394831 | 0.005843 |
| *GAPDH* | Glyceraldehyde-3-phosphate dehydrogenase | 1.18553 | 2.27446936 | 0.002549 |
| *RAD23B* | UV excision repair protein RAD23 homolog B | 1.18303 | 2.270531418 | 0.003528 |
| *FUBP1* | Far upstream element-binding protein 1 | 1.1813 | 2.267810354 | 0.003306 |
| *ALYREF* | THO complex subunit 4 | 1.1792 | 2.26451171 | 0.022692 |
| *U2AF2* | Splicing factor U2AF 65 kDa subunit | 1.1742 | 2.256677095 | 0.002011 |
| *ZYX* | Zyxin | 1.17123 | 2.252036172 | 0.007179 |
| *EDF1* | Endothelial differentiation-related factor 1 | 1.17067 | 2.251162186 | 0.004609 |
| *ARF1;*  *ARF3* | ADP-ribosylation factor 1;ADP-ribosylation factor 3 | 1.1658 | 2.243575914 | 0.013137 |
| *MMP14* | Matrix metalloproteinase-14 | 1.16573 | 2.243467057 | 0.011818 |
| *RPS16* | 40S ribosomal protein S16 | 1.16363 | 2.240203822 | 0.001389 |
| *RPS19* | 40S ribosomal protein S19 | 1.15127 | 2.221093305 | 0.002109 |
| *GABARAPL2* | Gamma-aminobutyric acid receptor-associated protein-like 2 | 1.15031 | 2.219615834 | 0.028455 |
| *TXN* | Thioredoxin | 1.14747 | 2.215250734 | 0.000047 |
| *BANF1* | Barrier-to-autointegration factor;Barrier-to-autointegration factor, N-terminally processed | 1.14547 | 2.212181872 | 0.000799 |
| *ARL3* | ADP-ribosylation factor-like protein 3 | 1.14477 | 2.211108775 | 0.004571 |
| *RPS4X;*  *RPS4Y2* | 40S ribosomal protein S4, X isoform;40S ribosomal protein S4, Y isoform 2 | 1.14463 | 2.210894218 | 0.000323 |
| *CHORDC1* | Cysteine and histidine-rich domain-containing protein 1 | 1.14147 | 2.206056896 | 0.015962 |
| *RPS15A* | 40S ribosomal protein S15a | 1.1404 | 2.204421342 | 0.012580 |
| *RPL8* | 60S ribosomal protein L8 | 1.13947 | 2.203000771 | 0.002005 |
| *PCBP2;*  *PCBP3* | Poly(rC)-binding protein 2;Poly(rC)-binding protein 3 | 1.13773 | 2.200345386 | 0.006965 |
| *LDLR* | Low-density lipoprotein receptor | 1.13587 | 2.19751041 | 0.001313 |
| *TUBA1A;*  *TUBA3C;*  *TUBA3E* | Tubulin alpha-1A chain;Tubulin alpha-3C/D chain;Tubulin alpha-3E chain | 1.1357 | 2.197251482 | 0.004938 |
| *PDAP1* | 28 kDa heat- and acid-stable phosphoprotein | 1.1329 | 2.192991165 | 0.015895 |
| *AK2* | Adenylate kinase 2, mitochondrial;Adenylate kinase 2, mitochondrial, N-terminally processed | 1.13033 | 2.189088074 | 0.030065 |
|  |  | 1.1297 | 2.188132346 | 0.015974 |
| *SKP1* | S-phase kinase-associated protein 1 | 1.12597 | 2.18248237 | 0.002929 |
| *TUBB3* | Tubulin beta-3 chain | 1.12593 | 2.18242186 | 0.002774 |
| *RAB7A* | Ras-related protein Rab-7a | 1.12347 | 2.178703691 | 0.002774 |
| *AHNAK* | Neuroblast differentiation-associated protein AHNAK | 1.1164 | 2.168052962 | 0.014236 |
| *RPS20* | 40S ribosomal protein S20 | 1.11633 | 2.16794777 | 0.005017 |
| *CCDC58* | Coiled-coil domain-containing protein 58 | 1.11437 | 2.165004465 | 0.020213 |
| *AK1* | Adenylate kinase isoenzyme 1 | 1.1116 | 2.160851606 | 0.001688 |
| *ERH* | Enhancer of rudimentary homolog | 1.11007 | 2.158561205 | 0.001219 |
| *EIF1AX;*  *EIF1AY* | Eukaryotic translation initiation factor 1A, X-chromosomal;Eukaryotic translation initiation factor 1A, Y-chromosomal | 1.10763 | 2.15491356 | 0.006976 |
| *TMOD3* | Tropomodulin-3 | 1.1071 | 2.15412206 | 0.008963 |
| *MOB1A;*  *MOB1B* | MOB kinase activator 1A;MOB kinase activator 1B | 1.10237 | 2.14707115 | 0.012715 |
| *BPNT1* | 3(2),5-bisphosphate nucleotidase 1 | 1.09267 | 2.132683679 | 0.011129 |
| *PFDN5* | Prefoldin subunit 5 | 1.08853 | 2.12657244 | 0.037542 |
| *EPS15L1* | Epidermal growth factor receptor substrate 15-like 1 | 1.08759 | 2.125187305 | 0.030899 |
| *TMA7* | Translation machinery-associated protein 7 | 1.08503 | 2.121419596 | 0.016510 |
| *UFM1* | Ubiquitin-fold modifier 1 | 1.08457 | 2.120743294 | 0.000880 |
| *RPS18* | 40S ribosomal protein S18 | 1.07407 | 2.10536446 | 0.002584 |
| *RPL14* | 60S ribosomal protein L14 | 1.0712 | 2.101180353 | 0.001857 |
| *NCL* | Nucleolin | 1.0705 | 2.100161102 | 0.002173 |
| *GLRX3* | Glutaredoxin-3 | 1.06963 | 2.098895006 | 0.002325 |
| *EIF1;*  *EIF1B* | Eukaryotic translation initiation factor 1;Eukaryotic translation initiation factor 1b | 1.06853 | 2.097295289 | 0.020704 |
| *DNAJC9* | DnaJ homolog subfamily C member 9 | 1.06783 | 2.096277922 | 0.000098 |
| *PCBP1* | Poly(rC)-binding protein 1 | 1.06697 | 2.095028689 | 0.014176 |
| *HSPA9* | Stress-70 protein, mitochondrial | 1.06573 | 2.09322878 | 0.010928 |
| *SEC22B* | Vesicle-trafficking protein SEC22b | 1.06513 | 2.092358412 | 0.001333 |
| *SARS* | Serine--tRNA ligase, cytoplasmic | 1.064 | 2.0907202 | 0.023718 |
| *RPS21* | 40S ribosomal protein S21 | 1.06067 | 2.085900007 | 0.018810 |
| *UBE2V1* | Ubiquitin-conjugating enzyme E2 variant 1 | 1.05827 | 2.082432886 | 0.013733 |
| *RPL24* | 60S ribosomal protein L24 | 1.05793 | 2.081942176 | 0.005031 |
| *SBDS* | Ribosome maturation protein SBDS | 1.05677 | 2.080268862 | 0.005773 |
| *IP* | Hepatoma-derived growth factor | 1.056 | 2.07915887 | 0.004185 |
| *UBE2N* | Ubiquitin-conjugating enzyme E2 N | 1.04223 | 2.05940846 | 0.002186 |
| *RPS2* | 40S ribosomal protein S2 | 1.04107 | 2.057753256 | 0.000780 |
| *HIST1H1B* | Histone H1.5 | 1.03887 | 2.054617731 | 0.004704 |
| *RPL5* | 60S ribosomal protein L5 | 1.0365 | 2.05124526 | 0.004615 |
| *GNPTG* | N-acetylglucosamine-1-phosphotransferase subunit gamma | 1.03647 | 2.051202606 | 0.024495 |
| *STRAP* | Serine-threonine kinase receptor-associated protein | 1.03257 | 2.045665131 | 0.048096 |
| *OLFML2B* | Olfactomedin-like protein 2B | 1.0316 | 2.044290185 | 0.020496 |
| *YBX1* | Nuclease-sensitive element-binding protein 1 | 1.03033 | 2.042491395 | 0.000063 |
| *SH3BGRL3* | SH3 domain-binding glutamic acid-rich-like protein 3 | 1.0281 | 2.039336717 | 0.015157 |
| *LIMA1* | LIM domain and actin-binding protein 1 | 1.02573 | 2.035989329 | 0.038784 |
| *TAGLN* | Transgelin | 1.02293 | 2.032041688 | 0.001537 |
| *PSAP* | Prosaposin;Saposin-A;Saposin-B-Val;Saposin-B;Saposin-C;Saposin-D | 1.0197 | 2.02749731 | 0.013163 |
| *CHMP2B* | Charged multivesicular body protein 2b | 1.01651 | 2.023019183 | 0.007882 |
| *RPS3A* | 40S ribosomal protein S3a | 1.01567 | 2.021841636 | 0.001087 |
| *HNRNPH1* | Heterogeneous nuclear ribonucleoprotein H;Heterogeneous nuclear ribonucleoprotein H, N-terminally processed | 1.01553 | 2.021645444 | 0.008411 |
| *DYNLL1* | Dynein light chain 1, cytoplasmic | 1.00963 | 2.01339467 | 0.010495 |
| *GNB2L1* | Guanine nucleotide-binding protein subunit beta-2-like 1;Guanine nucleotide-binding protein subunit beta-2-like 1, N-terminally processed | 1.00953 | 2.013255117 | 0.001925 |
| *BTF3L4* | Transcription factor BTF3 homolog 4 | 1.00867 | 2.01205536 | 0.027518 |
| *STIP1* | Stress-induced-phosphoprotein 1 | 1.00697 | 2.00968585 | 0.007596 |
| *DYNLRB1* | Dynein light chain roadblock-type 1 | 1.00597 | 2.008293325 | 0.013883 |
| *EIF3G* | Eukaryotic translation initiation factor 3 subunit G | 1.0055 | 2.007639171 | 0.009228 |
| *CSRP1* | Cysteine and glycine-rich protein 1 | 1.0034 | 2.004718959 | 0.018185 |
| *HMOX1* | Heme oxygenase 1 | 1.0008 | 2.001109343 | 0.003096 |
| *SGTA* | Small glutamine-rich tetratricopeptide repeat-containing protein alpha | 1.0005 | 2.000693267 | 0.006615 |
| *RTN4* | Reticulon-4 | 1.00007 | 2.000097043 | 0.026857 |
| *CFL2* | Cofilin-2 | 0.999299 | 1.999028444 | 0.008904 |
| *SEC13* | Protein SEC13 homolog | 0.9982 | 1.997506226 | 0.011458 |
| *RPS10* | 40S ribosomal protein S10 | 0.995767 | 1.994140416 | 0.030001 |
| *HSPE1* | 10 kDa heat shock protein, mitochondrial | 0.994233 | 1.992021198 | 0.007225 |
| *PPP1CA* | Serine/threonine-protein phosphatase PP1-alpha catalytic subunit | 0.993033 | 1.99036497 | 0.005407 |
| *RPS9* | 40S ribosomal protein S9 | 0.991266 | 1.987928681 | 0.000652 |
| *PPM1G* | Protein phosphatase 1G | 0.9887 | 1.984396063 | 0.002929 |
| *CTSC* | Dipeptidyl peptidase 1;Dipeptidyl peptidase 1 exclusion domain chain;Dipeptidyl peptidase 1 heavy chain;Dipeptidyl peptidase 1 light chain | 0.988033 | 1.983478831 | 0.014797 |
| *MTPN* | Myotrophin | 0.9831 | 1.976708313 | 0.009659 |
| *FAT1* | Protocadherin Fat 1;Protocadherin Fat 1, nuclear form | 0.979 | 1.971098674 | 0.030361 |
| *FKBP4* | Peptidyl-prolyl cis-trans isomerase FKBP4;Peptidyl-prolyl cis-trans isomerase FKBP4, N-terminally processed | 0.977934 | 1.969642777 | 0.002935 |
| *SFPQ* | Splicing factor, proline- and glutamine-rich | 0.977701 | 1.969324699 | 0.001692 |
| *NPM1* | Nucleophosmin | 0.976467 | 1.96764097 | 0.000247 |
| *CACYBP* | Calcyclin-binding protein | 0.973867 | 1.964098115 | 0.000276 |
| *RPS6* | 40S ribosomal protein S6 | 0.972599 | 1.962372606 | 0.012417 |
| *CHMP4B* | Charged multivesicular body protein 4b | 0.972033 | 1.961602877 | 0.029168 |
| *RPSA* | 40S ribosomal protein SA | 0.9695 | 1.95816183 | 0.005057 |
| *DDX1* | ATP-dependent RNA helicase DDX1 | 0.9689 | 1.957347623 | 0.000825 |
| *RPL7* | 60S ribosomal protein L7 | 0.968701 | 1.957077652 | 0.000551 |
| *RPS26;RPS26P11* | 40S ribosomal protein S26;Putative 40S ribosomal protein S26-like 1 | 0.9668 | 1.954500563 | 0.000231 |
| *UGDH* | UDP-glucose 6-dehydrogenase | 0.966101 | 1.953553817 | 0.001459 |
| *FAHD1* | Acylpyruvase FAHD1, mitochondrial | 0.965033 | 1.952108173 | 0.004866 |
| *TCEAL3;*  *TCEAL6* | Transcription elongation factor A protein-like 3;Transcription elongation factor A protein-like 6 | 0.964867 | 1.951883572 | 0.031473 |
| *SOD1* | Superoxide dismutase [Cu-Zn] | 0.964699 | 1.951656291 | 0.011010 |
| *ANP32A* | Acidic leucine-rich nuclear phosphoprotein 32 family member A | 0.963633 | 1.950214755 | 0.004524 |
| *CDV3* | Protein CDV3 homolog | 0.963367 | 1.949855213 | 0.010019 |
| *PFDN1* | Prefoldin subunit 1 | 0.9615 | 1.947333526 | 0.034173 |
| *UBXN1* | UBX domain-containing protein 1 | 0.960766 | 1.946343033 | 0.009716 |
| *HMGN1* | Non-histone chromosomal protein HMG-14 | 0.9607 | 1.946253994 | 0.011434 |
| *FABP5* | Fatty acid-binding protein, epidermal | 0.960433 | 1.945893834 | 0.002433 |
| *PDLIM4* | PDZ and LIM domain protein 4 | 0.958766 | 1.943646698 | 0.032536 |
| *IST1* | IST1 homolog | 0.9567 | 1.940865306 | 0.008590 |
| *PDXK* | Pyridoxal kinase | 0.954533 | 1.937952218 | 0.010026 |
| *HN1* | Hematological and neurological expressed 1 protein;Hematological and neurological expressed 1 protein, N-terminally processed | 0.950667 | 1.932766025 | 0.027963 |
| *CYCS* | Cytochrome c | 0.950066 | 1.931961039 | 0.004090 |
| *UBE2L3* | Ubiquitin-conjugating enzyme E2 L3 | 0.9496 | 1.931337103 | 0.001681 |
| *C14orf166* | UPF0568 protein C14orf166 | 0.9489 | 1.93040024 | 0.031400 |
| *NASP* | Nuclear autoantigenic sperm protein | 0.9485 | 1.929865094 | 0.003967 |
| *PPIH* | Peptidyl-prolyl cis-trans isomerase H | 0.944134 | 1.924033609 | 0.017302 |
| *CALD1* | Caldesmon | 0.943967 | 1.923810904 | 0.013273 |
| *LASP1* | LIM and SH3 domain protein 1 | 0.941267 | 1.920213864 | 0.018566 |
| *RPS3* | 40S ribosomal protein S3 | 0.936667 | 1.914101056 | 0.003920 |
| *EFHD2* | EF-hand domain-containing protein D2 | 0.935467 | 1.912509614 | 0.004407 |
| *RPS12* | 40S ribosomal protein S12 | 0.935433 | 1.912464542 | 0.025289 |
| *EIF4B* | Eukaryotic translation initiation factor 4B | 0.935401 | 1.912422123 | 0.005600 |
| *BASP1* | Brain acid soluble protein 1 | 0.934266 | 1.91091817 | 0.017899 |
| *STMN1* | Stathmin | 0.933102 | 1.909377018 | 0.005648 |
| *ST13;*  *ST13P5* | Hsc70-interacting protein;Putative protein FAM10A5 | 0.9305 | 1.905936429 | 0.011703 |
| *SUMO4* | Small ubiquitin-related modifier 4 | 0.9289 | 1.903823849 | 0.006610 |
| *SEC31A* | Protein transport protein Sec31A | 0.928834 | 1.903736756 | 0.007273 |
| *CNN2* | Calponin-2 | 0.928367 | 1.903120616 | 0.000889 |
| *PSME1* | Proteasome activator complex subunit 1 | 0.9241 | 1.89750015 | 0.017087 |
| *PCNP* | PEST proteolytic signal-containing nuclear protein | 0.922334 | 1.895178845 | 0.026410 |
| *EEF1G* | Elongation factor 1-gamma | 0.917 | 1.888184838 | 0.004409 |
| *LSM8* | U6 snRNA-associated Sm-like protein LSm8 | 0.916866 | 1.888009469 | 0.021988 |
| *CBR1* | Carbonyl reductase [NADPH] 1 | 0.914334 | 1.884698826 | 0.006130 |
| *BAG3* | BAG family molecular chaperone regulator 3 | 0.913701 | 1.883872073 | 0.031172 |
| *TAGLN2* | Transgelin-2 | 0.912067 | 1.881739603 | 0.003396 |
| *HLA-C* | HLA class I histocompatibility antigen, Cw-12 alpha chain;HLA class I histocompatibility antigen, Cw-16 alpha chain;HLA class I histocompatibility antigen, Cw-14 alpha chain | 0.910233 | 1.879348995 | 0.005664 |
| *PTBP1* | Polypyrimidine tract-binding protein 1 | 0.9102 | 1.879306008 | 0.018043 |
| *TBCB* | Tubulin-folding cofactor B | 0.905134 | 1.872718428 | 0.033045 |
| *ANP32B* | Acidic leucine-rich nuclear phosphoprotein 32 family member B | 0.900934 | 1.867274464 | 0.007626 |
| *FHL2* | Four and a half LIM domains protein 2 | 0.899833 | 1.865849988 | 0.013225 |
| *MARCKS* | Myristoylated alanine-rich C-kinase substrate | 0.899133 | 1.864944892 | 0.007466 |
| *RPL28* | 60S ribosomal protein L28 | 0.896967 | 1.862147045 | 0.009679 |
| *SUGT1* | Suppressor of G2 allele of SKP1 homolog | 0.895667 | 1.860469836 | 0.033618 |
| *MAP4* | Microtubule-associated protein 4 | 0.893666 | 1.857891176 | 0.011879 |
| *AAMDC* | Mth938 domain-containing protein | 0.889766 | 1.85287557 | 0.034810 |
| *PSMD1* | 26S proteasome non-ATPase regulatory subunit 1 | 0.888534 | 1.851293968 | 0.046579 |
| *TPM4* | Tropomyosin alpha-4 chain | 0.884134 | 1.845656405 | 0.004109 |
| *PTMA* | Prothymosin alpha;Prothymosin alpha, N-terminally processed;Thymosin alpha-1 | 0.8838 | 1.845229164 | 0.038956 |
| *SH3BGRL* | SH3 domain-binding glutamic acid-rich-like protein | 0.878532 | 1.838503598 | 0.005438 |
| *MYL6* | Myosin light polypeptide 6 | 0.871667 | 1.829775942 | 0.003869 |
| *EEF1B2* | Elongation factor 1-beta | 0.870466 | 1.828253343 | 0.018915 |
| *ENO1* | Alpha-enolase | 0.868132 | 1.825297976 | 0.002099 |
| *COL8A1* | Collagen alpha-1(VIII) chain;Vastatin | 0.862067 | 1.817640644 | 0.002820 |
| *ADI1* | 1,2-dihydroxy-3-keto-5-methylthiopentene dioxygenase | 0.8555 | 1.809385732 | 0.034154 |
| *LSM3* | U6 snRNA-associated Sm-like protein LSm3 | 0.853367 | 1.806712562 | 0.008965 |
| *CRK* | Adapter molecule crk | 0.853132 | 1.806418292 | 0.020905 |
| *SMPD1* | Sphingomyelin phosphodiesterase | 0.851366 | 1.804208412 | 0.021395 |
| *EEF1A1P5;*  *EEF1A1* | Putative elongation factor 1-alpha-like 3;Elongation factor 1-alpha 1 | 0.847733 | 1.799670763 | 0.021545 |
| *HLA-B* | HLA class I histocompatibility antigen, B-42 alpha chain;HLA class I histocompatibility antigen, B-8 alpha chain | 0.847532 | 1.799420046 | 0.013403 |
| *DUSP3* | Dual specificity protein phosphatase 3 | 0.847527 | 1.79941381 | 0.028592 |
| *ARHGDIA* | Rho GDP-dissociation inhibitor 1 | 0.847466 | 1.799337728 | 0.012526 |
| *TXNDC12* | Thioredoxin domain-containing protein 12 | 0.846668 | 1.798342733 | 0.016866 |
| *SET;*  *SETSIP* | Protein SET;Protein SETSIP | 0.841733 | 1.792201685 | 0.003666 |
| *DDX17* | Probable ATP-dependent RNA helicase DDX17 | 0.839833 | 1.789842946 | 0.032415 |
| *RAB2A* | Ras-related protein Rab-2A | 0.8378 | 1.787322532 | 0.000264 |
| *PFDN6* | Prefoldin subunit 6 | 0.836967 | 1.786290845 | 0.038136 |
| *EIF4H* | Eukaryotic translation initiation factor 4H | 0.834233 | 1.782908914 | 0.009906 |
| *ENSA* | Alpha-endosulfine | 0.833101 | 1.781510517 | 0.024614 |
| *PPIA* | Peptidyl-prolyl cis-trans isomerase A;Peptidyl-prolyl cis-trans isomerase A, N-terminally processed | 0.828167 | 1.775428178 | 0.003304 |
| *LRRFIP1* | Leucine-rich repeat flightless-interacting protein 1 | 0.8277 | 1.774853566 | 0.002367 |
| *USO1* | General vesicular transport factor p115 | 0.825801 | 1.772518887 | 0.033180 |
| *MAPRE1* | Microtubule-associated protein RP/EB family member 1 | 0.824834 | 1.771331213 | 0.012926 |
| *PGLS* | 6-phosphogluconolactonase | 0.821367 | 1.767079564 | 0.038456 |
| *TPD52L2* | Tumor protein D54 | 0.818 | 1.762960316 | 0.023146 |
| *EIF2S3;*  *EIF2S3L* | Eukaryotic translation initiation factor 2 subunit 3;Putative eukaryotic translation initiation factor 2 subunit 3-like protein | 0.817699 | 1.762592535 | 0.006045 |
| *PDLIM5* | PDZ and LIM domain protein 5 | 0.817567 | 1.762431273 | 0.000269 |
| *EIF3J* | Eukaryotic translation initiation factor 3 subunit J | 0.815133 | 1.759460347 | 0.008331 |
| *RPS27A;*  *UBA52;*  *UBB;*  *UBC* | Ubiquitin-40S ribosomal protein S27a;Ubiquitin;40S ribosomal protein S27a;Ubiquitin-60S ribosomal protein L40;Ubiquitin;60S ribosomal protein L40;Polyubiquitin-B;Ubiquitin;Polyubiquitin-C;Ubiquitin | 0.812 | 1.755643595 | 0.007609 |
| *CAP1* | Adenylyl cyclase-associated protein 1 | 0.809133 | 1.752158152 | 0.027747 |
| *DHX9* | ATP-dependent RNA helicase A | 0.809 | 1.75199663 | 0.000601 |
| *HNRNPC* | Heterogeneous nuclear ribonucleoproteins C1/C2 | 0.807867 | 1.750621265 | 0.008613 |
| *NACA* | Nascent polypeptide-associated complex subunit alpha;Nascent polypeptide-associated complex subunit alpha, muscle-specific form | 0.806833 | 1.749367019 | 0.005977 |
| *TUBB* | Tubulin beta chain | 0.805133 | 1.747306866 | 0.010828 |
| *SRP9* | Signal recognition particle 9 kDa protein | 0.799867 | 1.740940624 | 0.031681 |
| *PPP5C* | Serine/threonine-protein phosphatase 5 | 0.796874 | 1.737332631 | 0.032748 |
| *PFN1* | Profilin-1 | 0.793299 | 1.733032848 | 0.006539 |
| *NSFL1C* | NSFL1 cofactor p47 | 0.792066 | 1.731552344 | 0.003916 |
| *NAP1L4* | Nucleosome assembly protein 1-like 4 | 0.7907 | 1.729913618 | 0.007026 |
| *NAPA* | Alpha-soluble NSF attachment protein | 0.787666 | 1.726279418 | 0.001159 |
| *EIF2S1* | Eukaryotic translation initiation factor 2 subunit 1 | 0.784234 | 1.722177685 | 0.017319 |
| *CAST* | Calpastatin | 0.780634 | 1.717885641 | 0.013793 |
| *ADH5* | Alcohol dehydrogenase class-3 | 0.780466 | 1.717685607 | 0.010177 |
| *NTM* | Neurotrimin | 0.776334 | 1.712773049 | 0.023469 |
| *ZNF207* | BUB3-interacting and GLEBS motif-containing protein ZNF207 | 0.776 | 1.712376569 | 0.006756 |
| *CPPED1* | Serine/threonine-protein phosphatase CPPED1 | 0.771533 | 1.707082757 | 0.018554 |
|  | Galectin-1 | 0.770966 | 1.706411981 | 0.003160 |
| *FIS1* | Mitochondrial fission 1 protein | 0.768666 | 1.70369372 | 0.035823 |
| *PTPRG* | Receptor-type tyrosine-protein phosphatase gamma | 0.768508 | 1.703507147 | 0.028014 |
| *RPS17* | 40S ribosomal protein S17 | 0.765967 | 1.700509422 | 0.039257 |
| *YWHAE* | 14-3-3 protein epsilon | 0.765333 | 1.699762289 | 0.003719 |
| *PFN2* | Profilin-2 | 0.7568 | 1.689738505 | 0.007178 |
| *PPP1R18* | Phostensin | 0.756533 | 1.689425814 | 0.033692 |
| *RPL27* | 60S ribosomal protein L27 | 0.7549 | 1.687514619 | 0.000329 |
| *CIRBP* | Cold-inducible RNA-binding protein | 0.7549 | 1.687514619 | 0.017113 |
| *BOLA2* | BolA-like protein 2 | 0.752701 | 1.684944416 | 0.046628 |
| *SYNCRIP* | Heterogeneous nuclear ribonucleoprotein Q | 0.751033 | 1.682997461 | 0.008379 |
| *SFN* | 14-3-3 protein sigma | 0.749033 | 1.680665947 | 0.018182 |
| *UBA1* | Ubiquitin-like modifier-activating enzyme 1 | 0.747833 | 1.67926859 | 0.003162 |
| *ACTG1* | Actin, cytoplasmic 2;Actin, cytoplasmic 2, N-terminally processed | 0.7472 | 1.678531952 | 0.003990 |
| *NUDC* | Nuclear migration protein nudC | 0.741667 | 1.672106803 | 0.010553 |
| *GNS* | N-acetylglucosamine-6-sulfatase | 0.737334 | 1.66709232 | 0.014007 |
| *CTSB* | Cathepsin B;Cathepsin B light chain;Cathepsin B heavy chain | 0.7344 | 1.66370541 | 0.012825 |
| *PSMD9* | 26S proteasome non-ATPase regulatory subunit 9 | 0.733068 | 1.662170066 | 0.024721 |
| *YWHAZ* | 14-3-3 protein zeta/delta | 0.7286 | 1.657030319 | 0.004020 |
| *SPP1* | Osteopontin | 0.727767 | 1.65607384 | 0.008341 |
| *YWHAQ* | 14-3-3 protein theta | 0.726433 | 1.654543245 | 0.023603 |
| *CDC37* | Hsp90 co-chaperone Cdc37;Hsp90 co-chaperone Cdc37, N-terminally processed | 0.723066 | 1.65068633 | 0.014052 |
| *PSMD14* | 26S proteasome non-ATPase regulatory subunit 14 | 0.721299 | 1.648665822 | 0.029581 |
| *MYL12A;*  *MYL12B* | Myosin regulatory light chain 12A;Myosin regulatory light chain 12B | 0.721266 | 1.648628111 | 0.037802 |
| *RBM3* | RNA-binding protein 3 | 0.7203 | 1.647524592 | 0.010447 |
| *RPL30* | 60S ribosomal protein L30 | 0.720133 | 1.647333893 | 0.000471 |
| *HNRNPL* | Heterogeneous nuclear ribonucleoprotein L | 0.7199 | 1.647067865 | 0.019681 |
| *CAPRIN1* | Caprin-1 | 0.716967 | 1.643722776 | 0.027192 |
| *LRRC47* | Leucine-rich repeat-containing protein 47 | 0.716544 | 1.643240905 | 0.045311 |
| *PPA1* | Inorganic pyrophosphatase | 0.715599 | 1.642164895 | 0.015830 |
| *PPA2* | Inorganic pyrophosphatase 2, mitochondrial | 0.714966 | 1.641444533 | 0.016350 |
| *FKBP1A* | Peptidyl-prolyl cis-trans isomerase FKBP1A | 0.714833 | 1.641293218 | 0.013758 |
| *HSPB11* | Intraflagellar transport protein 25 homolog | 0.714799 | 1.641254538 | 0.001926 |
| *RAB21* | Ras-related protein Rab-21 | 0.713921 | 1.640256002 | 0.004590 |
| *SAFB* | Scaffold attachment factor B1 | 0.713867 | 1.640194608 | 0.036718 |
| *PABPC1;*  *PABPC3* | Polyadenylate-binding protein 1;Polyadenylate-binding protein 3 | 0.712867 | 1.639058106 | 0.009617 |
| *SDC4* | Syndecan-4 | 0.710534 | 1.636409707 | 0.023111 |
| *PHPT1* | 14 kDa phosphohistidine phosphatase | 0.709566 | 1.635312099 | 0.045627 |
| *PURB* | Transcriptional activator protein Pur-beta | 0.709467 | 1.635199885 | 0.022742 |
| *DBNL* | Drebrin-like protein | 0.704733 | 1.629843001 | 0.007330 |
| *COPS4* | COP9 signalosome complex subunit 4 | 0.7038 | 1.628789312 | 0.035852 |
| *TGM2* | Protein-glutamine gamma-glutamyltransferase 2 | 0.702833 | 1.627697944 | 0.008974 |
| *GNPNAT1* | Glucosamine 6-phosphate N-acetyltransferase | 0.7017 | 1.626420157 | 0.038922 |
| *EPRS* | Bifunctional glutamate/proline--tRNA ligase;Glutamate--tRNA ligase;Proline--tRNA ligase | 0.700333 | 1.624879801 | 0.041415 |
| *HNRNPD* | Heterogeneous nuclear ribonucleoprotein D0 | 0.695766 | 1.619744209 | 0.041303 |
| *NPC2* | Epididymal secretory protein E1 | 0.694866 | 1.618734075 | 0.045732 |
| *RBM8A* | RNA-binding protein 8A | 0.694733 | 1.618584853 | 0.048008 |
| *HEBP2* | Heme-binding protein 2 | 0.693867 | 1.617613564 | 0.005644 |
| *PITPNA* | Phosphatidylinositol transfer protein alpha isoform | 0.69386 | 1.617605716 | 0.001179 |
| *DDX6* | Probable ATP-dependent RNA helicase DDX6 | 0.693033 | 1.616678717 | 0.020006 |
| *HSP90AB1* | Heat shock protein HSP 90-beta | 0.690834 | 1.614216403 | 0.026848 |
| *CFL1* | Cofilin-1 | 0.6901 | 1.613395347 | 0.008829 |
| *DNAJC8* | DnaJ homolog subfamily C member 8 | 0.6856 | 1.608370745 | 0.036470 |
| *GSTO1* | Glutathione S-transferase omega-1 | 0.6847 | 1.607367704 | 0.001215 |
| *LMNA* | Prelamin-A/C;Lamin-A/C | 0.682433 | 1.604843927 | 0.005611 |
| *YWHAB* | 14-3-3 protein beta/alpha;14-3-3 protein beta/alpha, N-terminally processed | 0.681167 | 1.603436255 | 0.013082 |
| *EIF3E* | Eukaryotic translation initiation factor 3 subunit E | 0.6811 | 1.603361792 | 0.047876 |
| *EIF5A;*  *EIF5AL1* | Eukaryotic translation initiation factor 5A-1;Eukaryotic translation initiation factor 5A-1-like | 0.677066 | 1.598884804 | 0.006306 |
| *PCNA* | Proliferating cell nuclear antigen | 0.675933 | 1.597629636 | 0.020091 |
| *TPT1* | Translationally-controlled tumor protein | 0.675067 | 1.596670922 | 0.014227 |
| *PCMT1* | Protein-L-isoaspartate(D-aspartate) O-methyltransferase | 0.666234 | 1.586925059 | 0.003094 |
| *RPLP2* | 60S acidic ribosomal protein P2 | 0.661535 | 1.581764696 | 0.028435 |
| *RAB6A;*  *RAB6B;*  *RAB39A* | Ras-related protein Rab-6A;Ras-related protein Rab-6B;Ras-related protein Rab-39A | 0.661434 | 1.581653964 | 0.004956 |
| *PRKAR1A* | cAMP-dependent protein kinase type I-alpha regulatory subunit;cAMP-dependent protein kinase type I-alpha regulatory subunit, N-terminally processed | 0.658667 | 1.578623357 | 0.022140 |
| *TBCA* | Tubulin-specific chaperone A | 0.6552 | 1.574834256 | 0.027430 |
| *ACP1* | Low molecular weight phosphotyrosine protein phosphatase | 0.651566 | 1.570872403 | 0.028431 |
| *LRP1* | Prolow-density lipoprotein receptor-related protein 1;Low-density lipoprotein receptor-related protein 1 85 kDa subunit;Low-density lipoprotein receptor-related protein 1 515 kDa subunit;Low-density lipoprotein receptor-related protein 1 intracellular domain | 0.648901 | 1.567973308 | 0.000131 |
| *ERP29* | Endoplasmic reticulum resident protein 29 | 0.6468 | 1.565691527 | 0.048035 |
| *GLO1* | Lactoylglutathione lyase | 0.646434 | 1.565294374 | 0.040725 |
| *RPS11* | 40S ribosomal protein S11 | 0.639233 | 1.557500903 | 0.006848 |
| *MRC2* | C-type mannose receptor 2 | 0.636634 | 1.554697608 | 0.004398 |
| *PLBD2* | Putative phospholipase B-like 2;Putative phospholipase B-like 2 32 kDa form;Putative phospholipase B-like 2 45 kDa form | 0.6359 | 1.553906825 | 0.049618 |
| *CALU* | Calumenin | 0.630133 | 1.547707668 | 0.023500 |
| *MANF* | Mesencephalic astrocyte-derived neurotrophic factor | 0.6277 | 1.545099771 | 0.040740 |
| *CAPG* | Macrophage-capping protein | 0.624333 | 1.541497981 | 0.039750 |
| *TKT* | Transketolase | 0.623833 | 1.540963831 | 0.020321 |
| *PSME2* | Proteasome activator complex subunit 2 | 0.620166 | 1.537052028 | 0.021015 |
| *NAP1L1* | Nucleosome assembly protein 1-like 1 | 0.619233 | 1.536058328 | 0.012472 |
| *TPM3* | Tropomyosin alpha-3 chain | 0.6181 | 1.53485248 | 0.007297 |
| *PEA15* | Astrocytic phosphoprotein PEA-15 | 0.608201 | 1.524357193 | 0.014923 |
| *CASP7* | Caspase 7 | 0.6071 | 1.52319432 | 0.014661 |
| *IGF2R* | Cation-independent mannose-6-phosphate receptor | 0.6059 | 1.521927885 | 0.005008 |
| *PPP2R1A* | Serine/threonine-protein phosphatase 2A 65 kDa regulatory subunit A alpha isoform | 0.603366 | 1.519257064 | 0.013635 |
| *SRM* | Spermidine synthase | 0.602966 | 1.518835895 | 0.001424 |

**Supplemental Table 3.** 62 proteins were increased in Untreated CM by ≥1.5-fold (Log2Fold change≤0.6, *p<0.05).

| **Gene symbol** | **Protein name** | **Log2Fold change** | **Fold change** | **p-value** |
| --- | --- | --- | --- | --- |
| *AGRN* | Agrin;Agrin N-terminal 110 kDa subunit;Agrin C-terminal 110 kDa subunit;Agrin C-terminal 90 kDa fragment;Agrin C-terminal 22 kDa fragment | -2.6325 | 6.200996177 | 0.014063 |
| *SSC5D* | Soluble scavenger receptor cysteine-rich domain-containing protein SSC5D | -2.32474 | 5.009754824 | 0.000069 |
| *C1QTNF3* | Complement C1q tumor necrosis factor-related protein 3 | -2.28693 | 4.880165248 | 0.039778 |
| *FBLN1* | Fibulin-1 | -2.1929 | 4.572236407 | 0.028822 |
| *FST* | Follistatin | -2.04517 | 4.127219019 | 0.000713 |
| *PKP1* | Plakophilin-1 | -1.99096 | 3.975014161 | 0.046450 |
| *VWF* | von Willebrand factor;von Willebrand antigen 2 | -1.96173 | 3.895288004 | 0.039374 |
| *THBS3* | Thrombospondin-3 | -1.7683 | 3.406523124 | 0.024269 |
| *DKK1* | Dickkopf-related protein 1 | -1.72917 | 3.315370262 | 0.002113 |
| *COPS2* | COP9 signalosome complex subunit 2 | -1.637 | 3.110184144 | 0.030784 |
| *ICOSLG* | ICOS ligand | -1.58347 | 2.996898037 | 0.007743 |
| *ADAMTS5* | A disintegrin and metalloproteinase with thrombospondin motifs 5 | -1.57173 | 2.972609594 | 0.002505 |
| *MAP2K1* | Dual specificity mitogen-activated protein kinase kinase 1 | -1.33283 | 2.51896312 | 0.048653 |
| *CXCL1* | Growth-regulated alpha protein;GRO-alpha(4-73);GRO-alpha(5-73);GRO-alpha(6-73) | -1.32053 | 2.49757846 | 0.036996 |
| *CKM;CKB* | Creatine kinase M-type;Creatine kinase M-type, N-terminally processed;Creatine kinase B-type | -1.31245 | 2.483629547 | 0.027547 |
| *APMAP* | Adipocyte plasma membrane-associated protein | -1.2935 | 2.451220045 | 0.038350 |
| *HSPG2* | Basement membrane-specific heparan sulfate proteoglycan core protein;Endorepellin;LG3 peptide | -1.2776 | 2.424353371 | 0.020818 |
| *CTGF* | Connective tissue growth factor | -1.27723 | 2.423731691 | 0.001903 |
| *TNFAIP6* | Tumor necrosis factor-inducible gene 6 protein | -1.27137 | 2.413906841 | 0.005161 |
| *PTX3* | Pentraxin-related protein PTX3 | -1.2619 | 2.398113597 | 0.000407 |
| *S100A7;S100A7A* | Protein S100-A7;Protein S100-A7A | -1.21159 | 2.315927355 | 0.032932 |
| *MASP1* | Mannan-binding lectin serine protease 1;Mannan-binding lectin serine protease 1 heavy chain;Mannan-binding lectin serine protease 1 light chain | -1.19943 | 2.296489202 | 0.026894 |
| *EPG5* | Ectopic P granules protein 5 homolog | -1.15073 | 2.220262107 | 0.012804 |
| *ATP6V1F* | V-type proton ATPase subunit F | -1.12775 | 2.185176783 | 0.005966 |
| *FAM20C* | Extracellular serine/threonine protein kinase FAM20C | -1.0814 | 2.11608855 | 0.003315 |
| *PAMR1* | Inactive serine protease PAMR1 | -1.03927 | 2.055187471 | 0.016977 |
| *EGFR* | Epidermal growth factor receptor | -1.02173 | 2.030352186 | 0.011327 |
| *IGFBP4* | Insulin-like growth factor-binding protein 4 | -1.00587 | 2.008154125 | 0.000279 |
| *RARRES1* | Retinoic acid receptor responder protein 1 | -0.998167 | 1.997460536 | 0.008823 |
| *NID2* | Nidogen-2 | -0.973867 | 1.964098115 | 0.002277 |
| *VEGFC* | Vascular endothelial growth factor C | -0.953133 | 1.936072529 | 0.000131 |
| *PLAU* | Urokinase-type plasminogen activator;Urokinase-type plasminogen activator long chain A;Urokinase-type plasminogen activator short chain A;Urokinase-type plasminogen activator chain B | -0.951633 | 1.934060601 | 0.012639 |
| *CXCL12* | Stromal cell-derived factor 1;SDF-1-beta(3-72);SDF-1-alpha(3-67) | -0.947533 | 1.92857199 | 0.006962 |
| *SERPINF1* | Pigment epithelium-derived factor | -0.9386 | 1.91666739 | 0.017087 |
| *COL1A2* | Collagen alpha-2(I) chain | -0.934334 | 1.911008241 | 0.022485 |
| *ADAMTS1* | A disintegrin and metalloproteinase with thrombospondin motifs 1 | -0.912999 | 1.882955624 | 0.006625 |
| *C1R* | Complement C1r subcomponent;Complement C1r subcomponent heavy chain;Complement C1r subcomponent light chain | -0.894667 | 1.859180703 | 0.000602 |
| *PAPPA* | Pappalysin-1 | -0.8686 | 1.825890186 | 0.022378 |
| *COL3A1* | Collagen alpha-1(III) chain | -0.848733 | 1.800918632 | 0.028065 |
| *DCN* | Decorin | -0.842267 | 1.792865175 | 0.003426 |
| *DKK3* | Dickkopf-related protein 3 | -0.834867 | 1.783692595 | 0.005767 |
| *COL6A1* | Collagen alpha-1(VI) chain | -0.821301 | 1.766998726 | 0.012321 |
| *ANXA2;ANXA2P2* | Annexin A2;Putative annexin A2-like protein | -0.8117 | 1.755278557 | 0.007948 |
| *BTD* | Biotinidase | -0.809999 | 1.753210227 | 0.009083 |
| *GAS6* | Growth arrest-specific protein 6 | -0.798733 | 1.739572732 | 0.032614 |
| *GREM1* | Gremlin-1 | -0.763333 | 1.69740755 | 0.000059 |
| *PLAT* | Tissue-type plasminogen activator;Tissue-type plasminogen activator chain A;Tissue-type plasminogen activator chain B | -0.755067 | 1.687709969 | 0.013992 |
| *TIMP2* | Metalloproteinase inhibitor 2 | -0.7425 | 1.673072542 | 0.006959 |
| *FBLN1* | Fibulin-1 | -0.736233 | 1.665820556 | 0.027716 |
| *TF* | Serotransferrin | -0.719699 | 1.646838407 | 0.042409 |
| *COL1A1* | Collagen alpha-1(I) chain | -0.713066 | 1.639284207 | 0.022281 |
| *C1S* | Complement C1s subcomponent;Complement C1s subcomponent heavy chain;Complement C1s subcomponent light chain | -0.707633 | 1.633122487 | 0.034550 |
| *SERPING1* | Plasma protease C1 inhibitor | -0.700699 | 1.625292072 | 0.008512 |
| *THBS2* | Thrombospondin-2 | -0.687267 | 1.610230253 | 0.034056 |
| *ANXA1* | Annexin A1 | -0.6845 | 1.607144891 | 0.011063 |
| *LAMB1* | Laminin subunit beta-1 | -0.643499 | 1.562113196 | 0.019528 |
| *COL6A2* | Collagen alpha-2(VI) chain | -0.639134 | 1.557394029 | 0.036355 |
| *SRPX* | Sushi repeat-containing protein SRPX | -0.634567 | 1.552471732 | 0.028024 |
| *LUM* | Lumican | -0.622334 | 1.539363559 | 0.005990 |
| *LAMC1* | Laminin subunit gamma-1 | -0.621967 | 1.538972018 | 0.040519 |
| *ADAM9* | Disintegrin and metalloproteinase domain-containing protein 9 | -0.614766 | 1.531309604 | 0.004887 |
| *LAMA4* | Laminin subunit alpha-4 | -0.606698 | 1.522769944 | 0.002987 |
