# Supplementary material for: Discovery and validation of islet regenerative proteins secreted by human multipotent stromal cells
Source: Stem Cells Transl Med. 2026 Apr 29;15(5):szag022. doi: 10.1093/stcltm/szag022 (PMC13124281; doi:10.1093/stcltm/szag022)

**Supplemental Fig. 1. Treatment with CHIR99021 consistently elevated intracellular beta-catenin in human BM-MSC.** Intracellular beta-catenin was quantified by flow cytometry in 7 independent BM-MSC samples (N=7). (**a**) Representative flow cytometry plots of beta-catenin mean fluorescence intensity (MFI) in unstained MSC, untreated MSC, and Wnt+ MSC treated with 10 μM CHIR99021. (**b, c**) Wnt+ MSC showed increased intracellular beta-catenin levels compared to untreated MSC (paired Student’s t-test, **p<0.01). Data represent mean ± SEM.


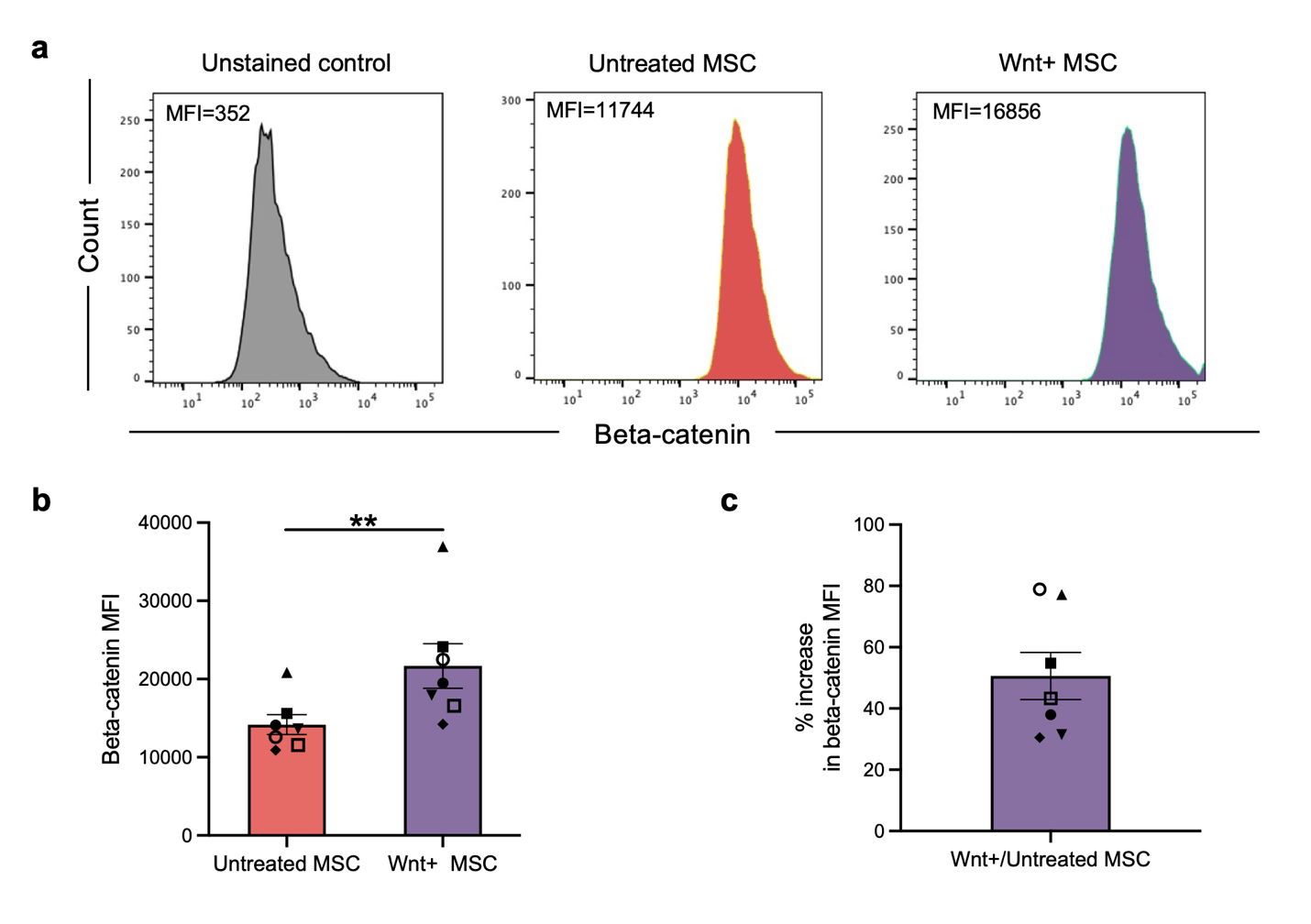

Supplement: szag022_Supplementary_Data [file szag022_supplementary_data.zip › Xie et al_SCTM_SupFig.docx]
